# Longitudinal Study of Frailty Phenotype in Relation to Chronic Kidney Disease Incidence

**DOI:** 10.1002/jcsm.70287

**Published:** 2026-04-21

**Authors:** Yong‐Xiang Ruan, Da‐Chuan Guo, Wen‐Hao Liu, Jia‐Man Ou, Qi Guo, Jing‐Wei Gao, Yang‐Wei Cai, Mao‐Xiong Wu, Xiao‐Tian Liang, Jie‐Wen Cai, Pin‐Ming Liu, Jing‐Feng Wang, Hai‐Feng Zhang, Yang‐Xin Chen

**Affiliations:** ^1^ Department of Cardiology Sun Yat‐sen Memorial Hospital of Sun Yat‐sen University Guangzhou China; ^2^ Guangzhou Key Laboratory of Molecular Mechanisms of Major Cardiovascular Disease Sun Yat‐sen Memorial Hospital of Sun Yat‐sen University Guangzhou China; ^3^ Guangdong Provincial Key Laboratory of Arrhythmia and Electrophysiology Sun Yat‐sen Memorial Hospital of Sun Yat‐sen University Guangzhou China; ^4^ Department of Cardiology Yangjiang Hospital of Guangdong Medical University Yangjiang Guangdong People's Republic of China; ^5^ Department of Cardiology, Beijing Hospital, National Center of Gerontology, Institute of Geriatric Medicine Chinese Academy of Medical Sciences Beijing China; ^6^ Graduate School Chinese Academy of Medical Sciences and Peking Union Medical College Beijing China

**Keywords:** chronic kidney disease, frailty phenotype, genetic predisposition, predictive model

## Abstract

**Background:**

The longitudinal relationship between frailty phenotype and CKD development, and the modifying role of genetic CKD risk in this association, remains unclear. Research on simple, noninvasive and quantifiable CKD prediction models incorporating frailty is limited.

**Methods:**

We analysed 214 502 CKD‐free participants from the UK Biobank cohort. Frailty was assessed using a modified phenotype with five components: weight loss, exhaustion, low grip strength, low physical activity and slow gait speed, to match UK Biobank data. Self‐reported walking pace and weight change served as proxies where objective measures were unavailable. A polygenic risk score for CKD was calculated based on 258 single nucleotide polymorphisms. Cox proportional hazards models were used to assess the association between the frailty phenotype and new‐onset CKD. The interaction between frailty and genetic risk on CKD outcomes was also examined. A noninvasive CKD prediction model, integrating frailty, age, gender, diabetes, hypertension, BMI and smoking status, was developed and validated internally using the UK Biobank and externally using CHARLS cohorts.

**Results:**

Among the 214 502 CKD‐free participants with a median age of 57 (49–62) years, 50.3% were female. A total of 109 290 individuals (51.0%) were classified as non‐frail, 96 941 (45.2%) as pre‐frail and 8271 (3.9%) as frail. Over the course of a median follow‐up period of 12.9 years, we documented 8079 (3.8%) cases of CKD. Compared with non‐frailty, the hazard ratio (HR) for new‐onset CKD in prefrailty and frailty was 1.143 (95% CI, 1.090–1.199, *p* < 0.001) and 1.606 (95% CI, 1.474–1.749, *p* < 0.001) in the multivariate model, respectively. Each one‐point increase in frailty score was associated with a higher risk of CKD (HR = 1.142; 95% CI, 1.116–1.170, *p* < 0.001) in the multivariable model. Participants with frailty and high genetic risk had the greatest risk of CKD (HR = 1.981; 95% CI, 1.733–2.266, *p* < 0.001) compared with those without frailty and low genetic risk. In the simple CKD prediction model incorporating frailty, it demonstrated an AUC of 0.734 at 5 years, 0.745 at 8 years and 0.749 at 10 years in internal testing. External validation also showed consistent discrimination and calibration with an AUC of 0.740.

**Conclusions:**

Pre‐frail and frail phenotypes were associated with a higher risk of developing CKD, showing a dose–response relationship. A noninvasive prediction model incorporating frailty and clinical parameters exhibited stable discriminative performance over a decade in both European and Asian cohorts.

**Abbreviations**
AUC: area under the curve
BMI: body mass index
BP: blood pressure
CI: confidence interval
CKD: chronic kidney disease
CRP: C‐reactive protein
DBP: diastolic blood pressure
eGFR: estimated glomerular filtration rate
HbA1c: haemoglobin A1c
HDL: high‐density lipoprotein
HR: hazard ratio
LDL: low‐density lipoprotein
Non‐HDL‐C: non‐high‐density lipoprotein cholesterol
NSAIDS: nonsteroidal anti‐inflammatory drugs
PRS: polygenic risk score
SBP: systolic blood pressure
SNP: single‐nucleotide polymorphism

## 1 Introduction

Chronic kidney disease (CKD) is characterised by a glomerular filtration rate (GFR) below 60 mL/min/1.73 m^2^, albuminuria exceeding 30 mg/24 h, or sustained indicators of kidney impairment lasting more than 3 months [1]. Epidemiological studies highlight the correlation between reduced eGFR and increased risks of mortality, cardiovascular events, hospitalisations and significant economic burden [2]. Despite the efforts to minimise the influence of some important established diseases, such as diabetes and hypertension, on the development of CKD, the prevalence of CKD is still high, affecting about 9.1% of the global population [3]. Because CKD is often irreversible [4], it is crucial to explore new factors related to its incidence to identify at‐risk populations and enable early prevention.

Frailty, a nuanced manifestation of an individual's overall condition, is a complex medical syndrome marked by diminished strength, endurance and compromised physiological functions, resulting in heightened sensitivity to stressors and potentially leading to institutionalisation, an elevation in morbidity rates and an escalation in mortality rates [5]. Among the myriad methods employed to assess frailty, frailty phenotype emerges as a leading conceptual model [6]. It is described by Fried and colleagues, who define frailty as the presence of three or more out of five indicators: weakness, slowness, weight loss, low physical activity and exhaustion [7].

Previous cross‐sectional studies investigated whether frailty phenotype was common in patients with CKD [8]. However, limited longitudinal evidence exists regarding the association between frailty phenotype and the onset of new CKD cases. Secondly, the occurrence and development of CKD are influenced by genetic factors [9], and the modifying role of genetic predisposition in the association between frailty and CKD risk remains unknown. Thirdly, the stealthy nature of CKD symptoms frequently results in underdiagnosis until identified via screening modalities such as urinalysis or blood tests [10]. Although previous predictive models for new‐onset CKD demonstrate decent discrimination and calibration, their practical application in daily life may present challenges due to the need for laboratory testing or even invasive procedures [11, 12]. The frailty phenotype is a noninvasive and easy‐to‐administer tool. Combining it with other self‐reported and widely accepted risk factors such as BMI, age, sex, smoking, diabetes, hypertension and sleep could improve screening rates for people at high risk of CKD, even before test results are available.

To illuminate gaps in current research, our study firstly harnessed data from the UK Biobank to perform Cox proportional hazards regression and competing risk analyses, estimating the association between frailty and the incidence of CKD, followed by a range of sensitivity analyses to test result robustness. Then, we applied PRS to explore whether the genetic risk of CKD modified the association between frailty and CKD. Finally, we further developed and assessed a widely acceptable, noninvasive and easily promotable risk prediction model for CKD, incorporating frailty phenotype, leveraging prospective cohort data from the UKB and externally validated this risk model using data from CHARLS.

## 2 Material and Methods

### 2.1 Data Source and Study Design

The UK Biobank is an ongoing population‐based prospective cohort. It was approved by the North West Research Ethics Committee and all participants signed informed consent [13]. The CHARLS, established in 2011, is a widely recognised longitudinal study that focuses on middle‐aged and older individuals (≥ 45 years) [14]. The details of the study design are clearly presented in Figures S1 and S2.

### 2.2 Assessment of Frailty Phenotype

The frailty phenotype contains five criteria, including weight loss, exhaustion, low physical activity, slow walking speed and low grip strength, which was first described by Fried et al. [7]. Given the difference in questions and measurements regarding frailty indicators in Fried's study, we modified definitions of several criteria to adapt the data for use in UK Biobank and CHARLS, as previously reported [15]. Detailed definitions are provided in Table S1, methodological details in the Supplementary Methods and additional supporting references in the Supplementary References. Specifically, self‐reported walking pace (‘How would you describe your usual walking pace?’) was used as a proxy for objectively measured gait speed, and self‐reported weight change compared with 1 year ago was used to approximate unintentional weight loss. These adaptations were made because objective assessments of gait speed and weight change were not uniformly collected in these cohorts. Importantly, while definitions of certain components were adapted to available data, the classification thresholds for frailty (frail ≥ 3, prefrail 1–2, non‐frail 0) were strictly retained as in Fried's original phenotype [7]. While this approach enables consistent assessment of frailty in large‐scale datasets, it may not fully replicate the original Fried phenotype and should be interpreted as a methodological adaptation. This limitation is further discussed in the ‘Discussion’ section.

### 2.3 Chronic Kidney Disease Assessment

Baseline CKD was identified as follows: in the UK Biobank [16]: (1) cases were obtained from records linked to inpatient services, death registries and primary care, and classified using the International Classification of Diseases (Table S2) [17]; (2) eGFR < 60 mL/min/1.73 m^2^ based on eGFR‐creatinine as appropriate (Supplementary Methods); (3) ACR in one single‐time urine sample > 30 mg/g. Incident CKD was identified through the International Classification of Diseases. The follow‐up period for each participant was determined from the date of their initial assessment, concluding at the earliest of the following events: the first occurrence of a new‐onset CKD diagnosis, the date of death or the conclusion of the follow‐up period, whichever came first.

In CHARLS, CKD was ascertained through eGFR, which was estimated using the CKD‐EPI creatinine equation [18]. If eGFR < 60 mL/min/1.73 m^2^, then the individual would be categorised as experiencing the first‐time CKD in his or her life [18].

### 2.4 Covariates Assessment

Information about covariates was collected through a touch‐screen questionnaire. The detailed description of them was available in Supplementary Methods and Tables S3 and S4. We further considered the number of long‐term morbidities (0, 1, 2, 3, 4 and 5 or more), which comprised 42 types of major chronic diseases through the verbal interview in the assessment centres by a physician and a trained nurse. The long‐term morbidities were initially developed for a large cross‐sectional study in Scotland and subsequently adapted for the UK Biobank [19]. We considered the number of long‐term morbidities because the index was associated with frailty and lifespan [6], and the detailed definitions were provided in Table S5. The detailed definitions of prevalent diabetes and hypertension at baseline and the calculation of the genetic risk score were provided in the Supplementary Methods.

### 2.5 Calculation of the Genetic Risk Score

UK Biobank genotyping, quality control and imputation procedures were run by the UK Biobank professional team and have previously been described in detail [20]. A total of 258 independent single nucleotide polymorphisms (SNPs) were selected, which were identified from the most recent GWAS and were significantly associated with CKD [21]; detailed information on the selected SNPs was provided in Table S6. The Polygenic Risk Score (PRS) for CKD was calculated by a method that has been described elsewhere: PRS = (β1 × SNP1 + β2 × SNP2 + … + β241 × SNP258) with each SNP coded 0, 1 or 2, according to the number of risk alleles. The β coefficient was obtained from the reported GWAS meta‐analysis. PRS was classified as high (quintile 3), intermediate (quintile 2) or low (quintile 1).

### 2.6 Statistical Analysis

Baseline characteristics were presented as mean (*SD*) or median (Q25, Q75) for continuous variables or proportions for categorical variables by frailty phenotype categories. ANOVA tests or Kruskal–Wallis tests were used for comparison of continuous variables based on the distribution, and the chi‐square test was conducted to analyse categorical variables.

The cumulative incidence of new‐onset CKD was calculated via the Kaplan–Meier method. The relationships of frailty phenotype with new‐onset CKD were estimated using Cox proportional hazards models. The linear trend test was performed by using the median of each quartile of the frailty phenotype score as a continuous variable in the regression model. The multivariate model adjusted for multiple covariates including age, sex, ethnicity, education, Townsend deprivation index, average household income, BMI, smoking status, alcohol frequency intake, sleep time, C‐reactive protein, albumin, high‐density lipoprotein, low‐density lipoprotein, triglycerides, total cholesterol, HbA1c, the number of long‐term morbidities, history of medication for cholesterol‐lowering, insulin, antihypertensive and NSAIDs and PRS. Nonlinear association between the frailty phenotype score and outcome was investigated using restricted cubic splines fitted into Cox proportional hazard models. We also examined the association between five components of frailty phenotype and their risk of CKD individually and mutually. Concurrently, the exploration of potential synergy between the frailty phenotype in the genesis of new‐onset CKD and the historical presence of diabetes and hypertension adhered to the methodologies previously elucidated. Subgroup analyses were conducted, wherein we examined the impact of age, sex, ethnicity, education, household income, smoking status and number of long‐term morbidities on the occurrence of new‐onset CKD through stratified analyses and interaction testing. Several sensitivity analyses were conducted. Firstly, we performed analyses using a 2‐year landmark analysis, excluding participants who experienced events within the initial 2 years of follow‐up. Secondly, we conducted analyses without imputing missing covariate data. Thirdly, we employed the Fine–Gray model to account for the competing risk of mortality. Finally, we conducted analyses in a population excluding individuals with baseline CKD defined by ICD codes and those with a baseline eGFR < 75 mL/min/1.73 m^2^. Missing data of covariates were imputed using multiple imputations by random forest with ‘mice’ of R. The code script and R packages used are detailed in the Supplementary Methods.

In delving into the potential synergistic intricacies of the frailty phenotype and the genetic predisposition to the onset of CKD, we probed for interaction by incorporating a cross‐product term of frailty phenotype and PRS into our analytical framework. Details of PRS calculation and analysis are provided in Supplementary Methods.

For the model development, the UKB data were internally validated by using the set seed function to divide the study population into training (70%) and testing (30%) cohorts [22]. Cox regression and LASSO regression were used to select the independent variables. The same method of variable analysis and model performance was applied to external validation within the CHARLS. The discriminative ability of the model was determined by the area under the curve (AUC) at 5, 8 and 10 year. Consistency between the predicted and actual risk of CKD was compared by drawing calibration curves, which were obtained from the bootstrapping method (500 replications) and Hosmer–Lemeshow goodness of fit test. Details of model development and validation are shown in Supplementary Methods.

All statistical analyses were performed using the R software version 4.2.3 (R Foundation for Statistical Computing). All the statistical tests were two‐sided, and *p* < 0.05 was considered statistically significant.

## 3 Results

### 3.1 Baseline Characteristics

Among the 214 502 participants, the mean age was 55.8 years, 50.3% were women and 95.7% were White. A detailed exposition of baseline characteristics is presented in Table 1. Within this diverse group, 8271 met the criteria for frailty (3.9%), 96 941 for prefrailty (45.2%) and 109 290 for non‐frailty (51.0%). People with prefrailty and frailty were characterised by an older age demographic and female, more deprived, lower education level and current smokers, and had higher BMI, more long‐term morbidities, higher PRS than those with non‐frailty. A comprehensive breakdown of characteristics of frailty is available in the Figures S3 and S4.

**TABLE 1:** Baseline characteristics by frailty category.

	Frailty phenotype			
Baseline characteristics	Non‐frailty	Prefrailty	Frailty	*p*
Number of participants, (%)	109 290	96 941	8271	
Age, years	56 (49 to 62)	57 (49 to 63)	58 (52 to 63)	< 0.001
Sex, *n* (%)				< 0.001
Male	56 944 (52.1)	46 480 (47.9)	3278 (39.6)	
Female	52 346 (47.9)	50 461 (52.1)	4993 (60.4)	
Ethnicity, *n* (%)				< 0.001
White	105 723 (96.7)	91 958 (94.9)	7533 (91.1)	
Others	3567 (3.3)	4983 (5.1)	738 (8.9)	
Education, *n* (%)				< 0.001
College/university degree	44 953 (41.1)	35 658 (36.8)	2121 (25.6)	
Others	64 337 (58.9)	61 283 (63.2)	6150 (74.4)	
Household income, *n* (%), £				< 0.001
Greater than 100 000	8061 (7.4)	5602 (5.8)	184 (2.2)	
52 000 to 100 000	26 923 (24.6)	21 051 (21.7)	954 (11.5)	
31 000 to 51 999	30 710 (28.1)	25 565 (26.4)	1551 (18.8)	
18 000 to 30 999	26 257 (24.0)	23 975 (24.7)	2108 (25.5)	
Less than 18 000	17 339 (15.9)	20 748 (21.4)	3474 (42.0)	
TDI	−2.4 (−3.8 to −0.1)	−2.2 (−3.7 to 0.4)	−0.9 (−3.1 to 2.2)	< 0.001
BMI, kg/m^2^	25.9 (23.6 to 28.4)	27.2 (24.5 to 30.1)	30.0 (26.4 to 34.6)	< 0.001
Smoking status, *n* (%)				< 0.001
Current	9868 (9.0)	10 447 (10.8)	1334 (16.1)	
Previous	38 174 (34.9)	34 165 (35.2)	2966 (35.9)	
Never	61 248 (56)	52 329 (54.0)	3971 (48)	
Alcohol intake, *n* (%)				< 0.001
Daily or almost daily	26 806 (24.5)	20 013 (20.6)	1140 (13.8)	
Once or twice a week	27 926 (25.6)	25 108 (25.9)	1878 (22.7)	
Three or four times a week	30 067 (27.5)	22 322 (23.0)	1090 (13.2)	
One to three times a month	10 645 (9.7)	11 368 (11.7)	1110 (13.4)	
Special occasions only	8444 (7.7)	10 865 (11.2)	1698 (20.5)	
Never	5402 (4.9)	7265 (7.5)	1355 (16.4)	
Sleep duration, *n* (%)	7 (7 to 8)	7 (6 to 8)	7 (6 to 8)	< 0.001
Laboratory results				
Albumin, g/L	45.5 (43.8 to 47.1)	45.1 (43.5 to 46.9)	44.4 (42.6 to 46.3)	< 0.001
C‐reactive protein, mg/L	1.1 (0.6 to 2.1)	1.4 (0.7 to 2.8)	2.4 (1.1 to 5.2)	< 0.001
eGFR, mL/min/1.73 m^2^	96.3 (86.9 to 108.8)	96.8 (87.4 to 107.1)	98.1 (87.8 to 110.5)	< 0.001
Serum total cholesterol, mmol/L	5.7 (5.0 to 6.4)	5.6 (4.9 to 6.4)	5.4 (4.5 to 6.2)	< 0.001
Triglycerides, mmol/L	1.4 (1.0 to 2.0)	1.5 (1.1 to 2.2)	1.7 (1.2 to 2.4)	< 0.001
HDL cholesterol, mmol/L	1.4 (1.2 to 1.7)	1.4 (1.1 to 1.6)	1.3 (1.1 to 1.5)	< 0.001
LDL cholesterol, mmol/L	3.5 (3.0 to 4.1)	3.5 (3.0 to 4.1)	3.4 (2.7 to 4.0)	< 0.001
HbA1c, mmol/mol	34.7 (32.4 to 37.1)	35.1 (32.7 to 37.8)	36.5 (33.5 to 40.2)	< 0.001
Number of long‐term morbidities				< 0.001
0	44 135 (40.4)	29 376 (30.3)	946 (11.4)	
1	36 523 (33.4)	31 783 (32.8)	1845 (22.3)	
2	18 307 (16.8)	20 097 (20.7)	2005 (24.2)	
3	6956 (6.4)	9689 (10.0)	1641 (19.8)	
4	2234 (2.0)	3816 (3.9)	965 (11.7)	
≥ 5	1135 (1.0)	2180 (2.2)	869 (10.5)	
PRS	−0.015978 (0.032578)	−0.015495 (0.032567)	0.01592 (0.032666)	0.008
Medication, *n* (%)				< 0.001
Blood pressure medication	8840 (8.1)	8653 (8.9)	861 (10.4)	
Cholesterol lowering	20 432 (18.7)	21 109 (21.8)	2398 (29.0)	
Insulin	178 (0.2)	172 (0.2)	26 (0.3)	
None of the above	79 840 (73.1)	67 007 (69.1)	4986 (60.3)	
NSAIDS, *n* (%)				< 0.001
Yes	26 247 (24.0)	27 603 (28.5)	3101 (37.5)	
No	83 043 (76.0)	69 338 (71.5)	5170 (62.5)	

*Note:* Data are mean (*SD*), median (IQR) or *n* (%).

Abbreviations: BMI, body mass index; eGFR, estimated glomerular filtration rate; HbA1c, glycated haemoglobin; HDL, high‐density lipoprotein; IQR, interquartile range; NSAIDS, nonsteroidal anti‐inflammatory drugs; PRS, Polygenic Risk Score; SD, standard deviation; TDI, Townsend deprivation index.

### 3.2 Frailty Is Associated With a Higher Risk of Incident CKD

**FIGURE 1:**
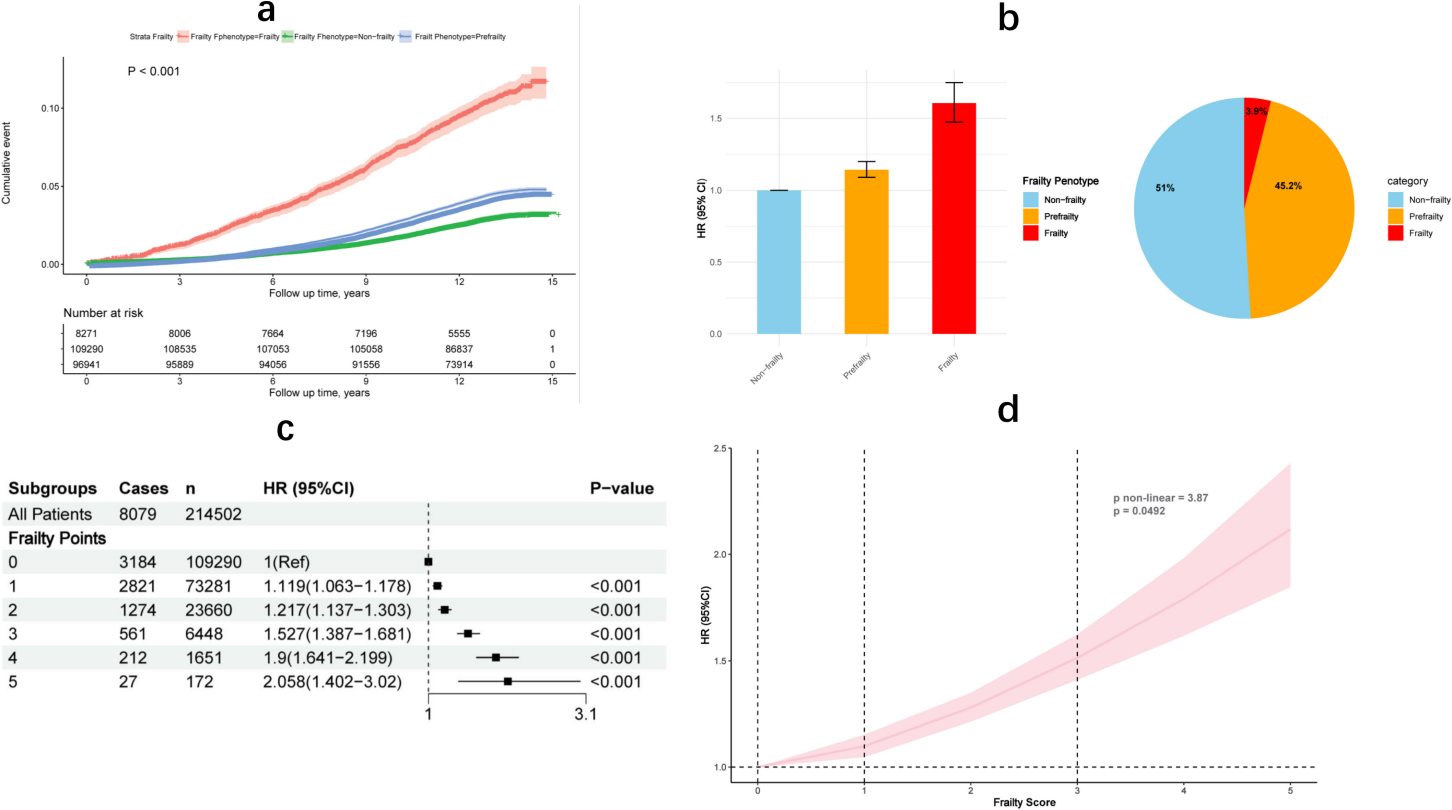
(a) The Kaplan–Meier curve of cumulative incidence of CKD by frailty phenotype. (b) Prospective association between frailty and incident CKD. (c) Prospective association between frailty points and incident CKD. (d) Nonlinear associations between number of individual components of the frailty phenotype and CKD. *p* non‐linear = 3.87, *p* = 0.049. The multivariate model adjusted for age, sex, ethnicity, education, Townsend deprivation index, average household income, BMI, smoking status, alcohol frequency intake, sleep time, eGFR, C‐reaction protein, albumin, high density lipoprotein, low density lipoprotein, triglyceride, total cholesterol, HbA1c, the number of long‐term morbidities, history of medication for cholesterol lowering, insulin, antihypertensive and NSAIDS and PRS.

Over the course of a median follow‐up period spanning 12.9 years, a total of 8079 cases of CKD (3.8%) were documented. The cumulative incidence graphically depicted graded relationships in alignment with the frailty phenotype throughout the observational period, as illustrated in Figure 1a (log‐rank test, *p* < 0.001).

Compared with non‐frailty participants, those with prefrailty (HR = 1.143; 95% CI, 1.090–1.199) and frailty (HR = 1.606; 95% CI, 1.474–1.749) exhibited an elevated risk of CKD in the multivariate model (Table 2; Figure 1b). This highlights a statistically significant graded relationship between frailty phenotype and CKD risk (*p* for trend < 0.001), even after adjusting for various covariates. The risk of developing CKD increases with higher frailty scores (Figure 1c). For each one‐point increase in frailty phenotype score, the risk of CKD escalated by 14.2% in the multivariate model (HR = 1.142; 95% CI, 1.116–1.170). All five components utilised to define frailty were independently associated with the risk of CKD (Table 3).

**TABLE 2:** Association between frailty phenotype and incident CKD.

	Non‐frailty	Prefrailty	Frailty	*p* for trend	Per 1‐point increase
Total case	3184	4095	800		
Total simple size	109 290	96 941	8271		
Unadjusted model, HR (95%CI)	1.00 (Ref.)	1.480 (1.413–1.550)	3.667 (3.393–3.962)	<0.001	1.455 (1.424–1.486)
Multivariate model, HR (95%CI)	1.00 (Ref.)	1.143 (1.090–1.199)	1.606 (1.474–1.749)	<0.001	1.142 (1.116–1.170)

*Note:* The multivariate model adjusted for age, sex, ethnicity, education, Townsend deprivation index, average household income, BMI, smoking status, alcohol frequency intake, sleep time, eGFR, C‐reaction protein, albumin, high density lipoprotein, low density lipoprotein, triglyceride, total cholesterol, HbA1c, the number of long‐term morbidities, history of medication for cholesterol lowering, insulin, antihypertensive and NSAIDS and PRS. Ref indicates reference.

**TABLE 3:** Individual components of frailty phenotype and their association with incident CKD.

Characteristic	HR (95%CI)	*p*	HR (95%CI)	*p*
Frailty component	Unadjusted model		Multivariate model	
Weight loss	1.197 (1.130–1.267)	< 0.001	1.101 (1.039–1.167)	0.001
Exhaustion	1.486 (1.398–1.578)	< 0.001	1.176 (1.102–1.255)	< 0.001
Low physical activity	1.259 (1.196–1.325)	< 0.001	1.061 (1.006–1.119)	0.031
Slow gait speed	3.147 (2.962–3.343)	< 0.001	1.333 (1.244–1.429)	< 0.001
Low grip strength	2.014 (1.918–2.115)	< 0.001	1.101 (1.045–1.160)	< 0.001

*Note:* The multivariate model adjusted for age, sex, ethnicity, education, Townsend deprivation index, average household income, BMI, smoking status, alcohol frequency intake, sleep time, eGFR, C‐reaction protein, albumin, high density lipoprotein, low density lipoprotein, triglyceride, total cholesterol, HbA1c, the number of long‐term morbidities, history of medication for cholesterol lowering, insulin, antihypertensive and NSAIDS, PRS and five individual components when these were not the exposure.

In addition, the graphical representation of the association between the frailty phenotype score and the incidence of CKD is shown in Figure 1d. We observed a positive nonlinear association between the cumulative number of frailty components and the incidence of CKD (*p* for nonlinearity = 0.049).

### 3.3 Integrated Analysis of Frailty Phenotype, Diabetes and Hypertension History

Results of joint analysis of frailty phenotype and histories of diabetes and hypertension on new‐onset CKD were provided in Figures S5 and S6. In contrast to individuals with a history of diabetes yet residing in a non‐frail state, those without a history of diabetes but in a pre‐frailty condition exhibited no discernible difference in their predisposition to CKD (HR = 0.898; 95% CI, 0.792–1.019). Similarly, when juxtaposed with non‐frail counterparts with hypertension, individuals devoid of a hypertensive past and entangled in frailty demonstrated no marked variance in CKD risk (HR = 1.125; 95% CI, 0.967–1.308). The consolidated findings, encompassing diverse categorisations such as frailty phenotype, hypertension and diabetes, have been delineated in Tables S7–10.

### 3.4 Subgroup Analysis and Sensitivity Analysis

**FIGURE 2:**
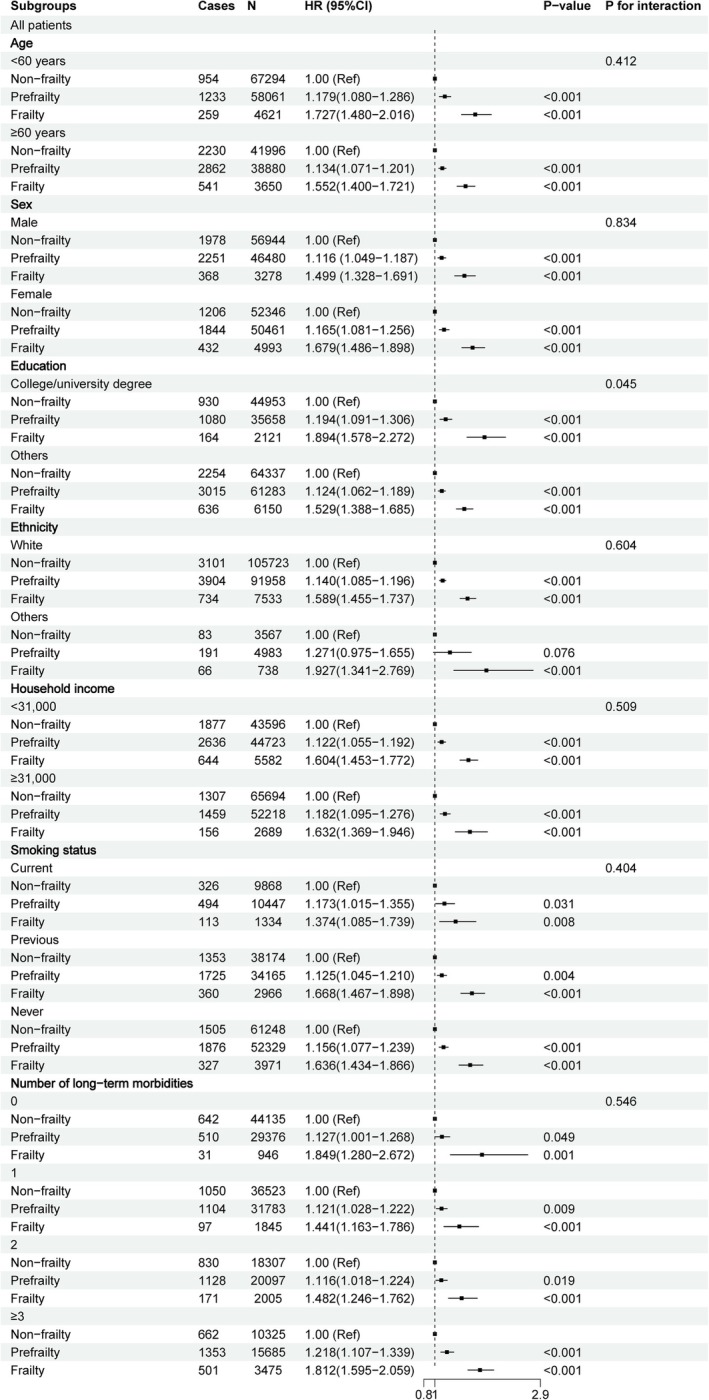
Subgroup analysis for the association between frailty phenotype and CKD. The multivariate model adjusted for age, sex, ethnicity, education, Townsend deprivation index, average household income, BMI, smoking status, alcohol frequency intake, sleep time, C‐reaction protein, eGFR, albumin, high density lipoprotein, low density lipoprotein, triglyceride, total cholesterol, HbA1c, the number of long‐term morbidities, history of medication for cholesterol lowering, insulin, antihypertensive and NSAIDS. For analyses on these factors, other factors were further adjusted.

Results of the subgroup analysis were provided in Figure 2. In sensitivity analyses, the integrity of our findings remained undisturbed when the analysis was reiterated without resorting to multiple imputation of covariates (Table S11). Furthermore, the exclusion of individuals who developed incident CKD within the initial 2 years of follow‐up and application of the Fine–Gray model did not substantively alter the outcomes (Figure S7; Tables S12 and S13). In a population where baseline CKD was excluded based on ICD codes, the outcome remained unchanged (Figure S8; Tables S14 and S15). The results were generally consistent after excluding those with a baseline eGFR < 75 mL/min/1.73 m^2^ (Figure S9; Tables S16 and S17).

### 3.5 Joint Analysis of Frailty Phenotype and PRS

**FIGURE 3:**
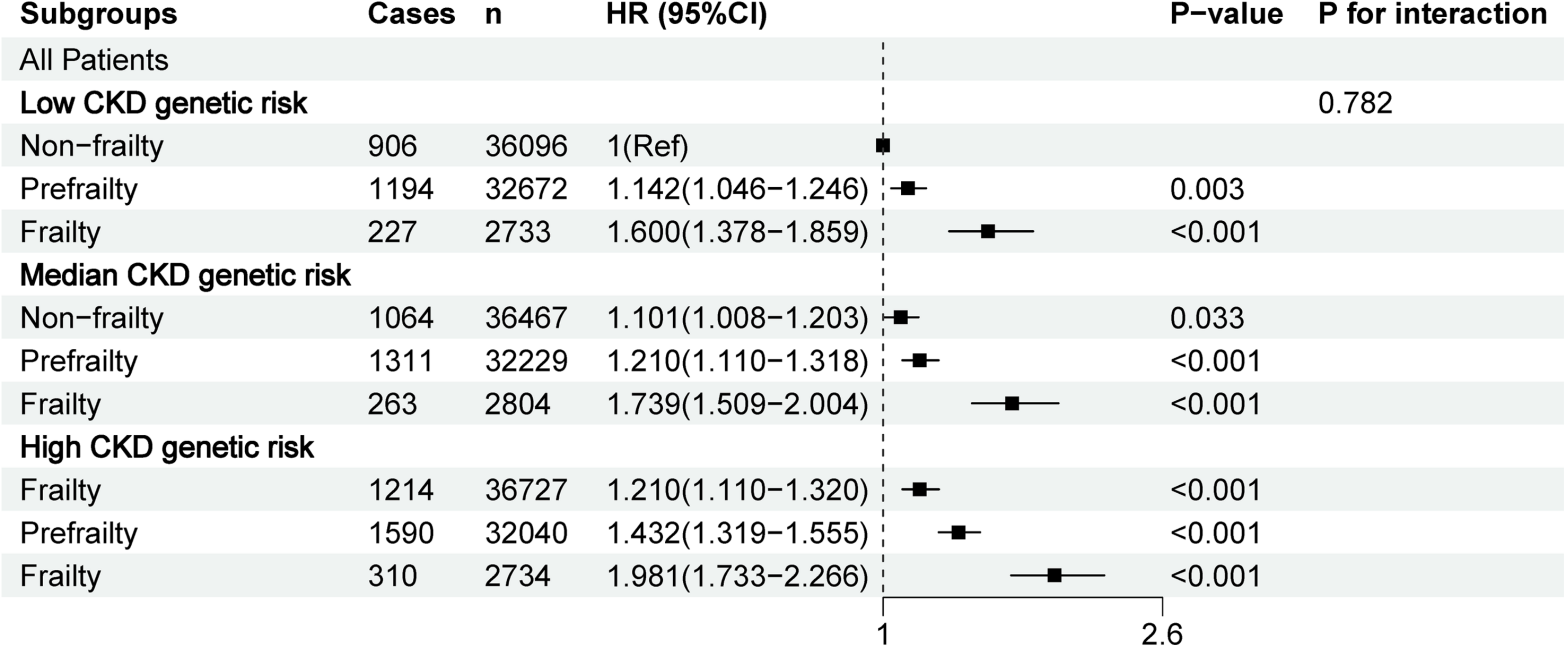
Joint association of frailty phenotype and PRS with CKD among the study population. The multivariate model adjusted for age, sex, ethnicity, education, Townsend deprivation index, average household income, BMI, smoking status, alcohol frequency intake, sleep time, C‐reaction protein, eGFR, albumin, high density lipoprotein, low density lipoprotein, triglyceride, total cholesterol, HbA1c, the number of long‐term morbidities, history of medication for cholesterol lowering, insulin, antihypertensive and NSAIDS.

Figure 3 illustrates the risk of incident CKD based on the combined categories of genetic risk and frailty phenotype. Participants with frailty and the highest tertile of PRS had the highest risk of CKD (HR = 1.981; 95% CI, 1.733–2.266) compared with those with non‐frailty and the lowest tertile of PRS. Using non‐frailty as a reference, participants with frailty had a high risk of incident CKD by 65.7% (HR = 1.657; 95% CI, 1.413–1.944), 53.1% (HR = 1.531; 95% CI, 1.319–1.777) and 65.3% (HR = 1.653; 95% CI, 1.439–1.898) in low‐, intermediate‐ and high‐PRS groups, respectively (Table S18). The associations between the PRS and the incidence of CKD in different strata of frailty phenotype are depicted in Table S19.

### 3.6 New‐Onset CKD Model Development and Validation

#### 3.6.1 Factor Selection

Sleep time was not associated with CKD development in the UKB training group (Tables S20 and S21). Other variables, including age, sex, BMI, smoking, frailty, diabetes and hypertension, remained independent predictors of incident CKD (all *p* < 0.001). On cubic spline regression, there was an escalation in CKD risk with increasing age, frailty and BMI (Figure S10). The correlation among these variables was evaluated prior to conducting LASSO regression. LASSO regression (Figures S11–S13) was used to develop the initial CKD risk model comprising seven predictive variables (four categorical: sex, smoking, hypertension and diabetes, and three continuous: age, BMI and frailty). A weight‐adjusted model was developed, expressed as the score (range: 0–14.5 points; Table S22).

#### 3.6.2 Patient Characteristics of the UK Biobank Cohort

Study characteristics are detailed in Table S23. Participants who developed CKD over the study period were predominantly male (54.5%), of older age [63 (IQR 58–66) years], with prevalent hypertension (65.5%), diabetes (16.2%), smoking habit (11.0%) and frailty (5.2%). The characteristics exhibited numerical similarity between the training group and the internal validation group within the UKB (Table S24).

#### 3.6.3 Internal Validation

**FIGURE 4:**
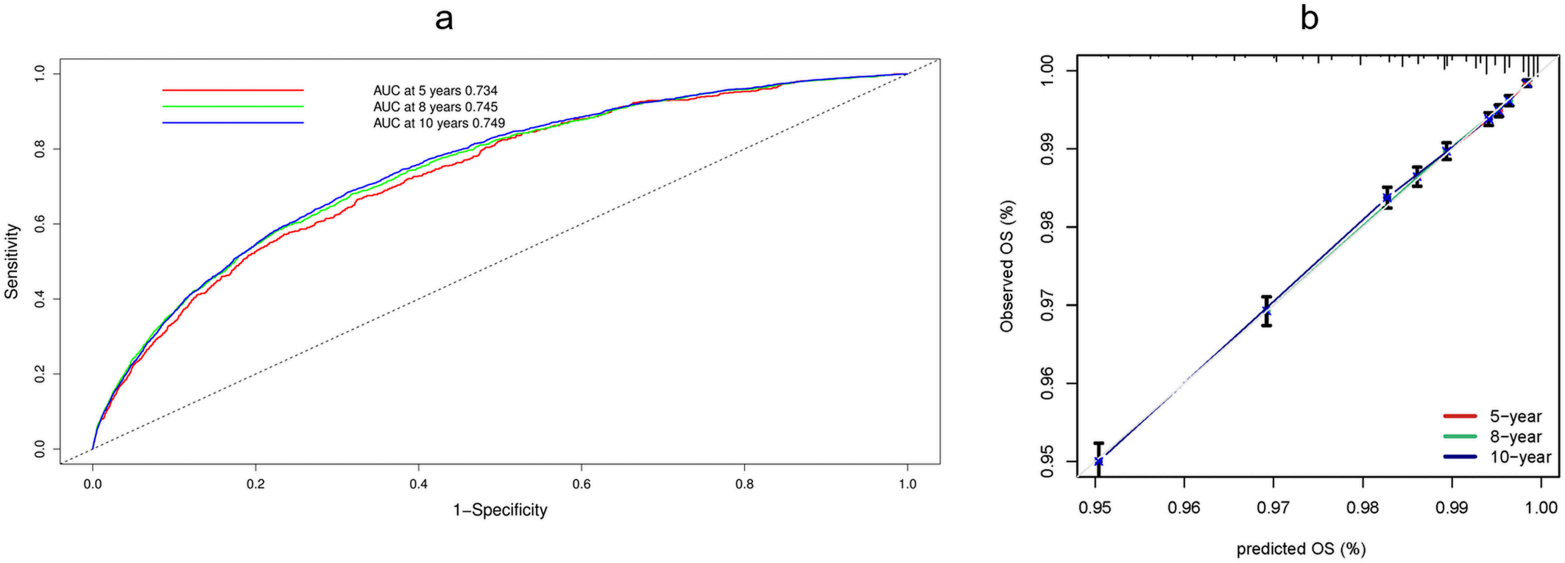
(a) Performance of the CKD model in predicting the risk of CKD in the UKB internal validation cohort at 5, 8 and 10 years. AUC, area under the curve. (b) Calibration plots for observed and risk of CKD in UKB internal validation cohort.

Our simple prediction model had an AUC of 0.740, 0.744 and 0.747 at 5, 8 and 10 years respectively in training cohort (Figure S14). The model had an AUC of 0.734, 0.745 and 0.749 at 5, 8 and 10 years respectively in internal testing cohort (Figure 4a). Calibration of the risk model is presented in Figure 4b. The AUC and calibration curves of the reconstructed prediction model after standardising the baseline and outcome definitions of CKD using ICD codes are presented in Figures S15 and S16. After excluding individuals with a baseline eGFR < 75 mL/min/1.73 m^2^, the AUC and calibration plots of the prediction model are shown in Figures S17 and S18.

The simplified score model maintained good predictive performance (Figure S19) with an AUC of 0.731 in the UKB internal validation cohort in 10 years and AUC of 0.727 in the UKB training cohort at 10 years. Five‐ and eight‐year risk model performance is presented in Figure S20.

Within the predefined internal validation cohort of the UKB, the Kaplan–Meier estimate of CKD risk demonstrated a linear relationship between rising risk score and CKD development (Figure S21). In the UKB internal validation cohort, an elevated score exhibited a positive correlation with escalated susceptibility to CKD (score 5–10: HR 4.170, 95% CI 3.851–4.517; score 10–14.5: HR 14.556, 95% CI 12.356–17.146).

#### 3.6.4 External Validation

CHARLS demographic characteristics are presented in Table S25. Disparities in trait distribution emerged between the UKB and CHARLS cohorts. Notably, the external validation cohort, characterised by a marginally advanced mean age of 58 years, boasted a higher representation of female participants at 54.9%. Furthermore, 15.6% of the cohort had a diagnosis of diabetes, while 25.2% presented with hypertension, and 19.1% exhibited frailty. Additionally, 38.1% of individuals reported a history of smoking. A comprehensive synopsis of baseline descriptive statistics is delineated in Table 4.

**TABLE 4:** Baseline characteristics of training and validation cohorts.

	Training group	Internal validation group	External validation group	*p*
	246 542	105 661	6139	
Age, years	57 (49–63)	57 (49–63)	58 (52–64)	< 0.001
Sex				0.002
Female, *n* (%)	129 870 (52.7)	55 535 (52.6)	3371 (54.9)	
BMI, kg/m^2^	26.5 (24–29.52)	27.17 (24.05–29.55)	23.28 (21.06–25.95)	< 0.001
Smoking, *n* (%)				< 0.001
Current smoking	24 411 (9.9)	10 434 (9.9)	2339 (38.1)	
Frailty phenotype, *n* (%)				< 0.001
Non‐frailty	143 101 (58)	60 819 (57.6)	437 (7.1)	
Prefrailty	98 079 (39.8)	42 555 (40.3)	4531 (73.8)	
Frailty	5362 (2.2)	2287 (2.2)	1171 (19.1)	
Diabetes, *n* (%)	14 660 (5.9)	6325 (6.0)	958 (15.6)	< 0.001
Hypertension, *n* (%)	111 788 (45.3)	47 859 (45.3)	1547 (25.2)	< 0.001

*Note:* Data are mean (*SD*), median (IQR) or *n* (%).

Among the CHARLS participants, the model had an AUC of 0.740 (Figure S22) and the score maintained its predictive performance with AUC of 0.710 (Figure S23). In CHARLS, there was a 30% heightened CKD risk for every 1‐point increase in the score overall (HR 1.371, 95% CI 1.296–1.452) and an incremental risk of CKD per risk category with a comparable time to CKD. Calibration of the risk model is presented in the CHARLS cohorts (Figures S24 and S25).

## 4 Discussion

Our study presents several valuable insights. Firstly, we identified frailty as a significant factor associated with an increased incidence of CKD, even after adjusting for various potential confounders, and confirmed the robustness of our findings through subgroup and sensitivity analyses. Secondly, we explored the interaction between the frailty phenotype and PRS in relation to CKD onset, providing new insights into this relationship. Thirdly, we developed, for the first time, a simple, user‐friendly and noninvasive CKD risk model that incorporates frailty phenotype, and both internal and external validations demonstrated its favourable discrimination and calibration.

### 4.1 Comparison With Other Studies

Firstly, although previous studies have established an association between frailty and CKD, our study extends this evidence by incorporating frailty, along with traditional CKD risk factors, into a predictive model. The model is relatively simple, noninvasive and based on readily available clinical and demographic variables. It showed acceptable performance in both internal and external validations.

Secondly, we constructed a more comprehensive PRS, including 258 SNPs—substantially more than the 27 SNPs used in the previous study [23]. Importantly, our PRS was developed using genetic and phenotypic data from European populations, thereby improving its accuracy and applicability to the study cohort. In contrast, prior research estimated PRS based on Asian genetic data and applied it to European samples, which may compromise the precision of risk estimation.

Thirdly, our analysis accounted for a broader range of covariates than the prior study [24], including triglycerides, total cholesterol, LDL, HDL and the use of nonsteroidal anti‐inflammatory drugs (NSAIDs), all of which are key factors in the development and progression of CKD [25, 26].

Finally, previous studies have explored the prognostic implications of frailty in patients with CKD [27, 28], further supporting the relevance of frailty as an important clinical factor. These findings indirectly reinforce our results, suggesting that frailty is not only associated with CKD occurrence but may also contribute to its adverse outcomes.

### 4.2 Pathophysiology of the Frailty Phenotype

Some pathophysiological mechanisms may explain the relationship between frailty and CKD. Firstly, individuals experiencing fatigue frequently manifest disturbances in oxidative metabolism and an accrual of lactic acid [29], consequently contributing to the impairment of renal vasculature. Moreover, individuals experiencing fatigue frequently exhibit indicators such as muscle weakness or compromised endurance. These manifestations constitute some of the most prevalent and distressing symptoms encountered by patients with CKD [30]. Secondly, grip strength and physical exercise are linked to human musculature, playing a pivotal role in sustaining protein metabolism, mitigating inflammation, providing antioxidant defence and regulating glucose metabolism within the body [31]. These pathophysiological processes have been unequivocally established as pivotal contributors to the onset of CKD [32]. Thirdly, the current research indicates that the renin‐angiotensin system (RAS) plays a crucial role in the occurrence and development of CKD [33, 34]. RAS regulates various factors, including inflammation, oxidation, vascular regulation and mitochondrial dysfunction, which are relevant to frailty [35].

### 4.3 The Significance of Clinical and Public Health Implications

The present study could have important clinical significance. This research distinguishes itself not only by demonstrating the association between frailty and an increased risk of CKD but also by developing a novel model that incorporates frailty alongside traditional CKD risk factors and requires no laboratory tests. Age and sex are important determinants of CKD risk and are readily incorporated into a CKD risk model, although they are unchangeable. Diabetes, hypertension, obesity and smoking are modifiable risk factors that can be targeted to potentially reduce CKD incidence and progression. Diabetes and hypertension emerge as primary catalysts in the initiation of CKD [36]. Smoking and CKD are closely interconnected; a meta‐analysis found that compared with never‐smokers, the summary relative risks of incident CKD were 1.34 (95% CI 1.23–1.47) for current smokers [37]. Wang et al. in a meta‐analysis found a positive association between BMI and risks for kidney disease outcomes [38]. These indicators are easy to obtain on a daily basis, avoiding invasive testing, and are therefore clinically generalisable. Although relatively well differentiated and calibrated, previous models are difficult to generalise to daily life because of the indicators that can only be obtained with laboratory tests or even invasive procedures [11, 12]. Our model underwent internal validation, and its predictive performance was further confirmed using an external Chinese cohort, underscoring its potential applicability across diverse populations. Given its potential clinical significance, integrating frailty assessment into CKD prevention guidelines may help clinicians optimise risk stratification and implement early intervention strategies.

Our study may offer useful insights for public health policies related to CKD prevention. Firstly, routine surveillance of frailty status—an easy and feasible measure—could play a crucial role in CKD prevention. Implementing frailty screening programs at the community level may help identify high‐risk individuals early, enabling targeted interventions [39] to mitigate frailty and potentially lower CKD incidence. Second, the development of a prediction model based on key risk factors allows for a more quantitative assessment of CKD risk. Its simplicity and feasibility make it accessible for broad application, including home‐based assessments, reducing reliance on complex laboratory analyses. Thus, at the individual level, awareness of frailty status and CKD risk can encourage proactive health management. Individuals may benefit from adopting preventive measures, such as lifestyle modifications and regular health monitoring, to help improve frailty status and potentially lower their CKD risk. Individuals who experience frailty may consider seeking further evaluation at healthcare facilities, where more thorough screening can be conducted to assess their CKD risk and guide appropriate interventions. Both strategies contribute to primary prevention efforts, emphasising the need for early risk identification and behavioural changes to promote kidney health.

### 4.4 Limitations

Several study constraints warrant acknowledgment. First, our external validation was conducted in a relatively small sample size (*n* = 6139), which may affect the accuracy of the predictive performance. Second, as an observational study, our research cannot establish a causal relationship between frailty phenotype and the onset of CKD. Despite our preliminary findings on the association between frailty and CKD, current research has yet to definitively demonstrate evidence supporting the mitigation of CKD incidence through frailty amelioration. Two components—gait speed and weight loss—were based on self‐reported rather than objective measures due to the absence of standardised physical performance testing in the UK Biobank. Although these proxies have been widely adapted in previous studies [see Supplementary References], they may still introduce misclassification bias. Nonetheless, consistent associations across multiple sensitivity and subgroup analyses suggest this limitation did not materially affect our findings. Finally, although our joint analysis of frailty and PRS suggests that genetic background may modify the association between frailty and CKD incidence, the results were based on a large set of SNPs (258 SNPs), which could limit clinical utility.

### 4.5 Future Directions

Firstly, longitudinal studies tracking frailty progression over time are needed to better understand the dynamic relationship between frailty and CKD risk. Second, as an observational study, our findings cannot establish causality. Future interventional trials should explore whether reversing frailty—through targeted lifestyle modifications or clinical interventions—can reduce the incidence of CKD. Third, currently established methods for preventing CKD, such as the use of RAS inhibitors in diabetic patients, could be considered for implementation in frail individuals to evaluate their effectiveness in reducing the incidence of CKD. These efforts will not only clarify the intricate link between frailty and CKD but also pave the way for more personalised and effective clinical decision‐making to improve patient outcomes.

## 5 Conclusions

In summary, the present study identifies frailty as a significant risk factor for CKD incidence. After comprehensive adjustment for covariates and sensitivity analyses, we consistently demonstrate the independent role of frailty in CKD development. Moreover, by incorporating frailty as a key variable, we introduce a simple and reliable model that can be readily used by the public, including nonmedical personnel, to predict CKD risk in individuals without pre‐existing CKD.

## Funding

This study was supported by grants from the National Natural Science Foundation of China (82070237; 82271609; 82170457; 82371573).

## Ethics Statement

We declare that our study has been approved by the appropriate ethics committee and has therefore been performed in accordance with the ethical standards laid down in the 1964 Declaration of Helsinki and its later amendments. Informed consents were obtained from all participants, and the study was approved by the North West Multi‐Centre Research Ethics Committee (11/NW/0382). The present work was conducted under the approval and access to data provided by the UK Biobank, with application number 91090. The CHARLS was approved by the Ethical Review Committee at the Peking University. Written informed consent was obtained from each participant prior to initiating any study procedures.

## Conflicts of Interest

The authors declare no conflicts of interest.

## Supporting information


**Table S1:** Definition of frailty phenotype and cutoff points.
**Table S2:** ICD 10 codes for identification of new‐onset CKD.
**Table S3:** Covariate definitions in UKB.
**Table S4:** Covariate definitions in CHARLS.
**Table S5:** Definition and list of long‐term morbidities.
**Table S6:** Variants used to make the genetic risk score for eGFR (using creatinine in the CKD‐EPI equation).
**Table S7:** Risk of incident CKD according to frailty phenotype stratified by diabetes.
**Table S8:** Risk of incident CKD according to diabetes stratified by different levels of frailty phenotype.
**Table S9:** Risk of incident CKD according to frailty phenotype stratified by hypertension.
**Table S10:** Risk of incident CKD according to hypertension stratified by different levels of frailty phenotype.
**Table S11:** Associations of frailty phenotype with the incidence of new‐onset CKD without multiple imputation.
**Table S12:** Associations of frailty phenotype with the incidence of new‐onset CKD ≥ 2 years from the baseline.
**Table S13:** Competing risk of associations of frailty phenotype with the incidence of new‐onset CKD.
**Table S14:** Association between frailty phenotype and incident CKD in a population excluding baseline CKD defined by ICD codes.
**Table S15:** Individual components of frailty phenotype and their association with incident CKD in a population excluding baseline CKD defined by ICD codes.
**Table S16:** Associations of frailty phenotype with the incidence of new‐onset CKD after excluding participants with baseline eGFR < 75 mL/min/1.73 m2.
**Table S17:** Individual components of frailty phenotype and their association with incident CKD after excluding participants with baseline eGFR < 75 mL/min/1.73 m2.
**Table S18:** Risk of incident CKD according to frailty phenotype stratified by different levels of PRS.
**Table S19:** Risk of incident CKD according to PRS stratified by different levels of frailty phenotype.
**Table S20:** Univariable analysis of variables.
**Table S21:** Multivariable predictors of CKD.
**Table S22:** CKD risk scoring system.
**Table S23:** Characteristics among the UKB.
**Table S24:** Characteristics among the train group and test group in UKB.
**Table S25:** Characteristics among the CHARLS.
**Figure S1:** Participant selection for frailty phenotype and CKD cohort study.
**Figure S2:** Participant selection for new‐onset CKD predictive model in UKB and CHARLS.
**Figure S3:** Prevalence of individuals components of the frailty phenotype at baseline.
**Figure S4:** Age‐specific distribution of frailty.
**Figure S5:** Combined effect of frailty phenotype and diabetes on new‐onset CKD.
**Figure S6:** Combined effect of frailty phenotype and hypertension on new‐onset CKD.
**Figure S7:** Competing risks cumulative incidence curve of frailty phenotype and new‐onset CKD.
**Figure S8:** Prospective association between frailty points and incident CKD in a population excluding baseline CKD defined by ICD codes.
**igure S9:** Prospective association between frailty points and incident CKD in a population excluding participants with baseline eGFR < 75 mL/min/1.73 m2.
**Figure S10:** Nonlinear associations between age, BMI, frailty score and CKD.
**Figure S11:** Spearman coefficient correlation between CKD prediction factors.
**Figure S12:** Regression coefficient variation curve with Log (λ).
**Figure S13:** The variation of mean square error with log (λ) in Lasso regression.
**Figure S14:** Performance of the model in predicting the risk of CKD in the UKB training cohort at 5, 8 and 10 years. AUC, area under the curve.
**Figure S15:** Discriminative performance of the model in predicting CKD risk in the UKB internal validation at 5, 8 and 10 years, based on CKD definitions using ICD codes. AUC, area under the curve.
**Figure S16:** Calibration plots showing the agreement between predicted and observed risk of CKD in the UKB internal validation cohort, using ICD code–based definitions of CKD.
**Figure S17:** Discriminative performance of the model in predicting CKD risk in the UKB internal validation at 5, 8 and 10 years, excluding participants with baseline eGFR < 75 mL/min/1.73 m2 AUC, area under the curve.
**Figure S18:** Calibration plots showing the agreement between predicted and observed risk of CKD based on the CKD risk score in the UKB internal validation cohort, excluding participants with baseline eGFR < 75 mL/min/1.73 m2.
**Figure S19:** Calibration plots for observed and risk of CKD used CKD risk score in UKB internal validation cohort.
**Figure S20:** Performance of the CKD risk score in predicting the risk of CKD in the UKB training cohort and testing cohort at 5, 8 and 10 years. AUC, area under the curve.
**Figure S21:** Cumulative incidence of CKD according to CKD risk score in internal validation cohort of the UKB. Log‐rank tests p < 0.001.
**Figure S22:** Performance of the CKD model in predicting the risk of CKD in CHARLS. AUC, area under the curve.
**Figure S23:** Performance of the CKD risk score in predicting the risk of CKD in CHARLS. AUC, area under the curve.
**Figure S24:** Calibration plots for observed and risk of CKD in CHARLS.
**Figure S25:** Calibration plots for observed and risk of CKD used CKD risk score in CHARLS.

## Data Availability

The datasets generated and analysed during the current study are available in the https://www.ukbiobank.ac.uk/researchers/ and https://charls.pku.edu.cn/.
