# Supplementary material for: Longitudinal Study of Frailty Phenotype in Relation to Chronic Kidney Disease Incidence
Source: J Cachexia Sarcopenia Muscle. 2026 Apr 21;17(3):e70287. doi: 10.1002/jcsm.70287 (PMC13099170; doi:10.1002/jcsm.70287)
Supplement: Supplementary file 1 — Table S1: Definition of frailty phenotype and cutoff points. Table S2: ICD 10 codes for identification of new‐onset CKD. Table S3: Covariate definitions in UKB. Table S4: Covariate definitions in CHARLS. Table S5: Definition and list of long‐term morbidities. Table S6: Variants used to make the genetic risk score for eGFR (using creatinine in the CKD‐EPI equation). Table S7: Risk of incident CKD according to frailty phenotype stratified by diabetes. Table S8: Risk of incident CKD according to diabetes stratified by different levels of frailty phenotype. Table S9: Risk of incident CKD according to frailty phenotype stratified by hypertension. Table S10: Risk of incident CKD according to hypertension stratified by different levels of frailty phenotype. Table S11: Associations of frailty phenotype with the incidence of new‐onset CKD without multiple imputation. Table S12: Associations of frailty phenotype with the incidence of new‐onset CKD ≥ 2 years from the baseline. Table S13: Competing risk of associations of frailty phenotype with the incidence of new‐onset CKD. Table S14: Association between frailty phenotype and incident CKD in a population excluding baseline CKD defined by ICD codes. Table S15: Individual components of frailty phenotype and their association with incident CKD in a population excluding baseline CKD defined by ICD codes. Table S16: Associations of frailty phenotype with the incidence of new‐onset CKD after excluding participants with baseline eGFR < 75 mL/min/1.73 m2. Table S17: Individual components of frailty phenotype and their association with incident CKD after excluding participants with baseline eGFR < 75 mL/min/1.73 m2. Table S18: Risk of incident CKD according to frailty phenotype stratified by different levels of PRS. Table S19: Risk of incident CKD according to PRS stratified by different levels of frailty phenotype. Table S20: Univariable analysis of variables. Table S21: Multivariable predictors of CKD. Table S22: CKD risk scoring sy [file JCSM-17-e70287-s001.doc]

**SUPPLEMENTARY MATERIAL**

**Longitudinal Study of Frailty Phenotype in Relation to CKD Incidence**

Yong-Xiang Ruan, MD, Wen-Hao Liu, PhD, Jia-Man, Ou, BM, Da-Chuan Guo, MD, Qi Guo, PhD, Jing-Wei Gao PhD, Yang-Wei Cai, PhD, Mao-Xiong Wu, PhD, Xiao-Tian Liang, MD, Jie-Wen Cai, MD, Pin-Ming Liu, PhD, Jing-Feng Wang, PhD, Hai-Feng Zhang, PhD, Yang-Xin Chen, PhD

**Contents**

[**Supplementary Methods 5**](#__RefHeading___Toc213707539)

[**Frailty phenotype in CHARLS 5**](#__RefHeading___Toc213707540)

[**The assessment of covariates 5**](#__RefHeading___Toc213707541)

[**Statistical Analysis 6**](#__RefHeading___Toc213707542)

[**R Code for Statistical Analysis 8**](#__RefHeading___Toc213707543)

[**R packages used in analysis 15**](#__RefHeading___Toc213707544)

[**Table S1. Definition of frailty phenotype and cutoff points 17**](#__RefHeading___Toc213707545)

[**Table S2. ICD 10 codes for identification of new-onset CKD 18**](#__RefHeading___Toc213707546)

[**Table S3. Co-variate definitions in UKB 19**](#__RefHeading___Toc213707547)

[**Table S4. Co-variate definitions in CHARLS 20**](#__RefHeading___Toc213707548)

[**Table S5. Definition and list of long-term morbidities 21**](#__RefHeading___Toc213707549)

[**Table S6. Variants used to make the genetic risk score for eGFR (using creatinine in the CKD-EPI equation) 25**](#__RefHeading___Toc213707550)

[**Table S7. Risk of incident CKD according to frailty phenotype stratified by diabetes 32**](#__RefHeading___Toc213707551)

[**Table S8. Risk of incident CKD according to diabetes stratified by different levels of frailty phenotype 32**](#__RefHeading___Toc213707552)

[**Table S9. Risk of incident CKD according to frailty phenotype stratified by hypertension 33**](#__RefHeading___Toc213707553)

[**Table S10. Risk of incident CKD according to hypertension stratified by different levels of frailty phenotype 33**](#__RefHeading___Toc213707554)

[**Table S11. Associations of frailty phenotype with the incidence of new-onset CKD without multiple imputation 34**](#__RefHeading___Toc213707555)

[**Table S12. Associations of frailty phenotype with the incidence of new-onset CKD ≥2 years from the baseline 35**](#__RefHeading___Toc213707556)

[**Table S13. Competing risk of associations of frailty phenotype with the incidence of new-onset CKD 36**](#__RefHeading___Toc213707557)

[**Table S14. Association between frailty phenotype and incident CKD in a population excluding baseline CKD defined by ICD codes 37**](#__RefHeading___Toc213707558)

[**Table S15. Individual components of frailty phenotype and their association with incident CKD in a population excluding baseline CKD defined by ICD codes 37**](#__RefHeading___Toc213707559)

[**Table S16. Associations of frailty phenotype with the incidence of new-onset CKD after excluding participants with baseline eGFR <75 mL/min/1.73m² 38**](#__RefHeading___Toc213707560)

[**Table S17. Individual components of frailty phenotype and their association with incident CKD after excluding participants with baseline eGFR <75 mL/min/1.73m² 38**](#__RefHeading___Toc213707561)

[**Table S18. Risk of incident CKD according to frailty phenotype stratified by different levels of PRS 39**](#__RefHeading___Toc213707562)

[**Table S19. Risk of incident CKD according to PRS stratified by different levels of frailty phenotype 39**](#__RefHeading___Toc213707563)

[**Table S20. Univariable analysis of variables 40**](#__RefHeading___Toc213707564)

[**Table S21. Multivariable predictors of CKD 40**](#__RefHeading___Toc213707565)

[**Table S22. CKD risk scoring system 41**](#__RefHeading___Toc213707566)

[**Table S23. Characteristics among the UKB 42**](#__RefHeading___Toc213707567)

[**Table S24. Characteristics among the train group and test group in UKB 43**](#__RefHeading___Toc213707568)

[**Table S25. Characteristics among the CHARLS 44**](#__RefHeading___Toc213707569)

[**Figure S1. Participant selection for frailty phenotype and CKD cohort study 45**](#__RefHeading___Toc213707570)

[**Figure S2. Participant selection for new-onset CKD predictive model in UKB and CHARLS 46**](#__RefHeading___Toc213707571)

[**Figure S3. Prevalence of individuals components of the frailty phenotype at baseline 47**](#__RefHeading___Toc213707572)

[**Figure S4. Age-specific distribution of frailty 48**](#__RefHeading___Toc213707573)

[**Figure S5. Combined effect of frailty phenotype and diabetes on new-onset CKD 49**](#__RefHeading___Toc213707574)

[**Figure S6. Combined effect of frailty phenotype and hypertension on new-onset CKD 50**](#__RefHeading___Toc213707575)

[**Figure S7. Competing risks cumulative incidence curve of frailty phenotype and new-onset CKD 51**](#__RefHeading___Toc213707576)

[**Figure S8. Prospective association between frailty points and incident CKD in a population excluding baseline CKD defined by ICD codes 52**](#__RefHeading___Toc213707577)

[**Figure S9. Prospective association between frailty points and incident CKD in a population excluding participants with baseline eGFR <75 mL/min/1.73m^2^ 53**](#__RefHeading___Toc213707578)

[**Figure S10. Non-linear associations between age, BMI, frailty score and CKD 54**](#__RefHeading___Toc213707580)

[**Figure S11. Spearman coefficient correlation between CKD prediction factors 55**](#__RefHeading___Toc213707581)

[**Figure S12. Regression coefficient variation curve with Log (λ) 56**](#__RefHeading___Toc213707582)

[**Figure S13. The variation of mean square error with log (λ) in Lasso regression 57**](#__RefHeading___Toc213707583)

[**Figure S14. Performance of the model in predicting the risk of CKD in the UKB training cohort at 5, 8 and 10 years. AUC, area under the curve 58**](#__RefHeading___Toc213707584)

[**Figure S15. Discriminative performance of the model in predicting CKD risk in the UKB internal validation at 5, 8, and 10 years, based on CKD definitions using ICD codes. AUC: area under the curve 59**](#__RefHeading___Toc213707585)

[**Figure S16. Calibration plots showing the agreement between predicted and observed risk of CKD in the UKB internal validation cohort, using ICD code–based definitions of CKD 60**](#__RefHeading___Toc213707586)

[**Figure S17. Discriminative performance of the model in predicting CKD risk in the UKB internal validation at 5, 8, and 10 years, excluding participants with baseline eGFR <75 mL/min/1.73m² AUC: area under the curve 61**](#__RefHeading___Toc213707587)

[**Figure S18. Calibration plots showing the agreement between predicted and observed risk of CKD based on the CKD risk score in the UKB internal validation cohort, excluding participants with baseline eGFR <75 mL/min/1.73m² 62**](#__RefHeading___Toc213707589)

[**Figure S19. Calibration plots for observed and risk of CKD used CKD risk score in UKB internal validation cohort 63**](#__RefHeading___Toc213707590)

[**Figure S20. Performance of the CKD risk score in predicting the risk of CKD in the UKB training cohort and testing cohort at 5, 8 and 10 years. AUC, area under the curve 64**](#__RefHeading___Toc213707591)

[**Figure S21. Cumulative incidence of CKD according to CKD risk score in internal validation cohort of the UKB. Log-rank tests *p*＜0.001. 65**](#__RefHeading___Toc213707592)

[**Figure S22. Performance of the CKD model in predicting the risk of CKD in CHARLS. AUC, area under the curve 66**](#__RefHeading___Toc213707593)

[**Figure S23. Performance of the CKD risk score in predicting the risk of CKD in CHARLS. AUC, area under the curve 67**](#__RefHeading___Toc213707594)

[**Figure S24. Calibration plots for observed and risk of CKD in CHARLS 68**](#__RefHeading___Toc213707595)

[**Figure S25. Calibration plots for observed and risk of CKD used CKD risk score in CHARLS 69**](#__RefHeading___Toc213707596)

[**Supplementary References 70**](#__RefHeading___Toc213707597)

**Supplementary Methods**

**Frailty phenotype in CHARLS**

We modified frailty phenotype to adapt the data for use in CHARLS. Slowness was defined if gait speed, measured using the average of two trials of walk tests over a 2.5 m course, was at or below the 20^th^ percentile of the gender- and height-adjusted population distribution. The criteria for weakness were met when the maximum handgrip strength was below or equal to the 20^th^ percentile of the population distribution, after adjusting for gender and BMI. Exhaustion was defined when the answer to the two questions from the modified Centre for Epidemiological Studies-Depression (CES-D) scale (“I could not get going,” “I felt everything I did was an effort”) was “occasionally or a moderate amount of the time” or “most of the time.” Participants met the criteria for inactivity if they answered “no” to the question of “during a usual week, did you walk for at least 10 min continuously?” Respondents met the shrinking criteria when they currently had a BMI ≤ of 18.5 kg/m^2^ or self-reported loss of at least 5 kg in the previous year[1].

**We calculated the eGFR by the Chronic Kidney Disease Epidemiology Collaboration (CKD-EPI) equation as follows[2]:**

eGFR = 141 × [minimum (serum creatinine/κ, 1)α] × [maximum (serum creatinine/κ, 1)−1.209]×(0.993age)×1.018 [if female], where α is − 0.329 for females and − 0.411 for males and κ is 0.7 for females and 0.9 for males.

**The assessment of covariates**

Information about covariates was collected through a touch-screen questionnaire, including age, sex, ethnicity, self-reported average annual household income, education level, Townsend deprivation index and sleep duration. Alcohol intake was self-reported as never, special occasions only, 1 to 3× per month, once or twice per week, 3 to 4× per week, and daily or almost daily. Smoking status was categorized as never, previous, and current. Body mass index was calculated as weight in kilograms divided by squared height in meters (kg/m^2^). C-reactive protein was measured by immunoturbidimetric - high sensitivity analysis on a Beckman Coulter AU5800. Albumin was measured by BCG analysis on a Beckman Coulter AU5800. Total cholesterol was measured by the CHO-POD analysis, and high-density lipoprotein cholesterol was measured by enzyme immunoinhibition on a Beckman Coulter AU5800. Low-density lipoprotein cholesterol was measured by enzymatic protective selection analysis on a Beckman Coulter AU5800. Triglyceride was measured by GPO-POD analysis on a Beckman Coulter AU5800. HbA1c (glycated hemoglobin) was measured by HPLC analysis on a Bio-Rad VARIANT II Turbo. Medication use for diabetes, hypertension, hyperlipidemia, Nonsteroidal Anti-inflammatory Drugs (NSAIDS) were obtained from self-reports.

Prevalent diabetes and hypertension at baseline encompassed an evaluation of the self-reported diabetes diagnosis, prior hospital diagnosis before enrollment time, prescription of any glucose-lowering medication, blood glucose ≥7.0 mmol/l, and HbA1c ≥48 mmol/mol (≥6.5%) at baseline[3]. Hypertension was defined as systolic blood pressure (SBP) ≥ 140 mmHg, diastolic blood pressure (DBP) ≥ 90 mmHg on two occasions, self-reported antihypertensive treatment, or self-reported hypertension history, or International Classification of Diseases ICD-10 (I10-I15)[4].

| **Diagnosis** | **Field ID** | **Codes (if applicable)** |
| --- | --- | --- |
| Diabetes status at baseline | 41270 ICD10  41280 IDC10–date  20002 Self–reported  2443 Diabetes diagnosed by doctor  6153 Medication  6177 Medication  20003 Treatment/medication code  30750 HbA1c  30740 Glucose | E10–, E11–, E12–, E13–, E14–, G59.0, G63.2, H28.0, H36.0, M14.2, N08.3  1220;1221;1222;1223;1276;1468;1607  –  Insulin  Insulin  Medications for diabetes  ≥48.0 mmol/mol  ≥7.0 mmol/L |

**Statistical Analysis**

The UKB data were internally validated by using the set seed function to divide the study population into training (70%) and testing (30%) cohorts. CHARLS is used for external validation. First, we evaluated the correlation among various variables and univariable Cox regression was employed to identified independent predictors of CKD. Nonlinear association between age, BMI, frailty and outcome was investigated using restricted cubic splines fitted into Cox proportional hazard models. Next, statistically significant variables identified in the univariable Cox regression were included in the multivariable Cox regression. Following this, selected variables were submitted to a least absolute shrinkage and selection operator (LASSO) and ten-fold cross-validation was used for optimal shrinkage parameter selection. The lowest partial likelihood deviance was selected, which included seven (four categorical and three continuous) variables.

The final selected variables were incorporated into the Cox proportional hazards regression model, and regression coefficients (β) were calculated for each risk factor. Age was categorized into four groups with intervals of 10 years each (38-47, 48-57, 58-67, >68). BMI is classified according to WHO standards as <18.5 kg/m^2^, 18.5-24.9 kg/m^2^, 25-29.9 kg/m^2^, and ≥30 kg/m^2^.Participants were classified as frailty (≥3 scores) if they fulfilled three or more of the five criteria, prefrailty (1 to 2 scores) if they fulfilled one or two criteria, and those who did not encounter any criteria were considered to have non-frailty (0 scores). The median value within each group was chosen as the reference value, denoted as W_ij_. Set age 38-47, female gender, BMI <18.5 kg/m², absence of frailty, non-smoking, and no history of diabetes and hypertension as individual reference points. Then, calculate the distance D between each group of risk factors and the basic risk reference value WiREF, using the reference value W_ij_ for each group of risk factors. The calculation formula is D=(W_ij_-Wi_REF_)* β_i_. Next, we set an age increase of 5 years as 1 point, denoted as a constant B. The calculation formula is Points_ij_=D/B=(W_ij_-Wi_REF_)×β_i_/B, and finally round the calculated value to the nearest whole number, which is the corresponding score for the group. The method has been reported before[5-7].

Cox proportional hazards regression was performed to derive the median time to CKD and hazard ratio for each 1-point increment in the risk score with score zero as the reference. Risk scores were further stratified into those with a score of zero and risk tertiles as follows: score 0–5, score 5–10, and score 10–14.5, based on CKD probabilities from the regression model. Model-based individual risk of CKD was calculated using Kaplan–Meier survival curves according to risk groups.

The discriminative ability of the model was determined by the area under the curve (AUC) at 5, 8, 10 years and C‐statistic. Consistency between the predicted and actual risk of CKD was compared by drawing calibration curves, which was obtained from the bootstrapping method (500 replications) and Hosmer-Lemeshow goodness of fit test.

**R Code for Statistical Analysis**

#Categorical variables were converted into factor variables before model fitting:

m1$Frailty_Phenotype <- as.factor(m1$Frailty_Phenotype)

m1$Sex <- as.factor(m1$Sex)

m1$Ethnicity <- as.factor(m1$Ethnicity)

m1$Education <- as.factor(m1$Education)

m1$Income <- as.factor(m1$Income)

m1$Smoking_Status <- as.factor(m1$Smoking_Status)

m1$Alcohol_Intake <- as.factor(m1$Alcohol_Intake)

m1$Comorbidity <- as.factor(m1$Comorbidity)

m1$Medication_Use <- as.factor(m1$Medication_Use)

m1$NSAIDs_Use <- as.factor(m1$NSAIDs_Use)

#A multivariable Cox proportional hazards model was fitted as follows:

model_cox <- coxph(Surv(Followup_Years, CKD_Outcome) ~ Frailty_Phenotype + Sex + Age + Ethnicity + Education + Income + BMI + TDI + Smoking_Status + Sleep_Duration + Alcohol_Intake + Albumin + egfr + Triglycerides + Total_Cholesterol + CRP + LDL + HDL + HbA1c + Comorbidity + PRS + Medication_Use + NSAIDs_Use, x = TRUE, y = TRUE, data = m1)

# Fit the Kaplan-Meier survival model

surv_model <- survfit(Surv(time = m1$Followup_Years, event = m1$CKD_Outcome) ~ Frailty_Phenotype, data = m1)

# Plot the survival curves

ggsurvplot(surv_model,

risk.table = TRUE, # Add risk table

fun = "event", # Plot cumulative incidence (1 - survival probability)

conf.int = TRUE, # Show confidence interval

conf.int.style = "ribbon",

pval = TRUE, # Display log-rank test p-value

test.for.trend = FALSE,

legend.title = "Frailty Phenotype",

xlab = "Follow-up Time (Years)", # Label x-axis

break.x.by = 3, # Set x-axis breakpoints

data = m1)

# Fit Cox proportional hazards model with restricted cubic splines (RCS)

m3 <- cph(Surv(years, ICD10_ckd_f) ~ rcs(Frailty.points, 4) + Sex + Age + Ethnicity + Education + Income + BMI + TDI + Smoking_Status + Sleep_Duration + Alcohol_Intake + Albumin + egfr + Triglycerides + Total_Cholesterol + CRP + LDL + HDL + HbA1c + Comorbidity + PRS + Medication_Use + NSAIDs_Use, x = TRUE, y = TRUE, data = m1)

# Perform ANOVA to assess the overall contribution of RCS terms

anova(m3)

# Define data distribution for prediction

dd <- datadist(m1)

options(datadist = 'dd')

# Generate predicted hazard ratios (HRs) across frailty points

HR <- Predict(m3, Frailty.points, fun = exp, ref.zero = TRUE)

View(HR)

P1<-ggplot(HR)

P2<-ggplot()+geom_line(data=HR,aes(Frailty.points,yhat),linetype="solid",size=1,alpha = 0.7,colour="pink")+geom_ribbon(data=HR, aes(Frailty.points,ymin = lower, ymax = upper),alpha = 0.5,fill="pink")

P2<-P2+theme_classic()+geom_hline(yintercept=1, linetype=2,size=0.5)+

geom_vline(xintercept=0,linetype=2,size=0.5)+geom_vline(xintercept=1,linetype=2,size=0.5)+geom_vline(xintercept=3,linetype=2,size=0.5)+labs(x="Frailty.points", y="HR (95%CI)")

# Fine-Gray Model for Frailty Phenotype

# Create model matrix for categorical frailty phenotype (excluding intercept)

cov <- model.matrix(~m1$Frailty.phenotype.1)[,-1]

# Fit Fine-Gray competing risks model

fit <- crr(m1$years, m1$outcome, cov, failcode = 1, cencode = 0)

# Fit Fine-Gray model using frailty points as a continuous variable

fit3 <- crr(m1$years, m1$outcome, m1$Frailty.points, failcode = 1, cencode = 0)

# Select relevant covariates

multvar <- m1[, c("Frailty.points", "egfr", "Sex", "Age", "Ethnicity",

"Education", "BMI", "TDI", "Income", "smoking", "sleep",

"alcohol.intake", "alb", "TG", "TC", "CRP", "LDL", "HDL",

"Comorbidity", "PRS", "NSAIDs_Use", "HbA1c",

"Medication_Use")]

# Attach multivariable dataset for ease of reference

attach(multvar)

# Create design matrix for the Fine-Gray model (excluding intercept)

cov <- model.matrix(~ Frailty.points + egfr + Sex + Age + Ethnicity +

Education + Income + BMI + TDI + smoking + sleep +

alcohol.intake + alb + TG + TC + CRP + LDL + HDL +

HbA1c + Comorbidity + PRS +

Medication_Use + NSAIDs_Use)[,-1]

# Fit Fine-Gray competing risks model with multiple covariates

fit <- crr(m1$years, m1$outcome, cov, failcode = 1, cencode = 0)

# Select relevant covariates for the Fine-Gray model

multvar = m1[, c("Frailty.phenotype.1", "egfr", "Sex", "Age", "Ethnicity", "Education", "BMI", "TDI", "Income", "smoking", "sleep", "alcohol.intake", "alb", "TG", "TC", "CRP", "LDL", "HDL", "Comorbidity", "PRS", "NSAIDs_Use", "HbA1c", "Medication_Use")]

# Attach the multivariable dataset

attach(multvar)

# Create design matrix for the Fine-Gray model (excluding intercept)

cov = model.matrix(~Frailty.phenotype.1 + egfr + Sex + Age + Ethnicity + Education + BMI + TDI + Income + smoking + sleep + alcohol.intake + alb + TG + TC + CRP + LDL + HDL + Comorbidity + PRS + NSAIDs_Use + HbA1c + Medication_Use)[,-1]

# Fit Fine-Gray competing risks model

fit <- crr(m1$years, m1$outcome, cov, failcode = 1, cencode = 0)

#Model Validation

set.seed(123) # Set seed for reproducibility

train_index <- sample(1:nrow(m1), nrow(m1) * 0.7) # 70% training, 30% testing

train_data <- m1[train_index, ]

test_data <- m1[-train_index, ]

# Convert categorical variables to factors in the training dataset

train_data$sex <- as.factor(train_data$sex)

train_data$smoking <- as.factor(train_data$smoking)

train_data$frailty.phenotype <- as.factor(train_data$frailty.phenotype)

train_data$base_diabete <- as.factor(train_data$base_diabete)

train_data$base_hypertension <- as.factor(train_data$base_hypertension)

# Perform univariate Cox regression for selected variables

modelcox1 <- coxph(Surv(ICD10_ckd_f_time, ICD10_ckd_f) ~ sex, data = train_data)

modelcox2 <- coxph(Surv(ICD10_ckd_f_time, ICD10_ckd_f) ~ age, data = train_data)

modelcox3 <- coxph(Surv(ICD10_ckd_f_time, ICD10_ckd_f) ~ BMI, data = train_data)

modelcox4 <- coxph(Surv(ICD10_ckd_f_time, ICD10_ckd_f) ~ smoking, data = train_data)

modelcox5 <- coxph(Surv(ICD10_ckd_f_time, ICD10_ckd_f) ~ frailty.phenotype, data = train_data)

modelcox6 <- coxph(Surv(ICD10_ckd_f_time, ICD10_ckd_f) ~ base_diabete, data = train_data)

modelcox7 <- coxph(Surv(ICD10_ckd_f_time, ICD10_ckd_f) ~ base_hypertension, data = train_data)

modelcox8 <- coxph(Surv(ICD10_ckd_f_time, ICD10_ckd_f) ~ sex + age + BMI + smoking + frailty.phenotype + base_diabete + base_hypertension, data = train_data)

# Construct matrix for LASSO

x <- as.matrix(train_data[, 2:8]) # Select predictors from columns 2 to 8

y <- Surv(train_data$ICD10_ckd_f_time, train_data$ICD10_ckd_f == 1) # Define survival outcome

set.seed(123)

lasso_model <- glmnet(x, y, family = "cox", alpha = 1) # Fit LASSO model

plot(lasso_model, xvar = "lambda", label = TRUE) # Plot LASSO coefficient path

# Cross-validation for optimal lambda

fitCV <- cv.glmnet(x, y, family = "cox", type.measure = "deviance", alpha = 1, nfolds = 10)

plot(fitCV)

fitCV$lambda.1se

coef(fitCV, s = "lambda.1se")

fitCV$lambda.min

coef(fitCV, s = "lambda.min")

# Convert categorical variables to factors in the test dataset

test_data$sex <- as.factor(test_data$sex)

test_data$smoking <- as.factor(test_data$smoking)

test_data$frailty.phenotype <- as.factor(test_data$frailty.phenotype)

test_data$base_diabete <- as.factor(test_data$base_diabete)

test_data$base_hypertension <- as.factor(test_data$base_hypertension)

# Perform Cox regression on test data

f1 <- coxph(Surv(ICD10_ckd_f_time, ICD10_ckd_f) ~ sex + age + BMI + smoking + frailty.phenotype + base_diabete + base_hypertension, data = test_data)

# Compute C-index for internal validation

dd <- datadist(test_data)

options(datadist = "dd")

f2 <- cph(Surv(ICD10_ckd_f_time, ICD10_ckd_f) ~ sex + age + BMI + smoking + frailty.phenotype + base_diabete + base_hypertension, data = test_data)

summary(f2)

validate(f2, method = "boot", B = 1000, dxy = TRUE)

# Time-dependent ROC analysis

predicted_values <- predict(f2, newdata = test_data, type = "lp")

tROC <- timeROC(T = test_data$ICD10_ckd_f_time, delta = test_data$ICD10_ckd_f, marker = predicted_values, cause = 1, times = c(5, 8, 10), ROC = TRUE)

plot(tROC, time = 5, col = "red", lwd = 2)

plot(tROC, time = 8, col = "green", add = TRUE, lwd = 2)

plot(tROC, time = 10, col = "blue", add = TRUE, lwd = 2)

pdf(file = "calibration.pdf", width = 6, height = 6)

# 5-year calibration

f <- cph(Surv(ICD10_ckd_f_time, ICD10_ckd_f) ~ sex + age + BMI + smoking + frailty.phenotype + base_diabete + base_hypertension, data = test_data, time.inc = 5)

cal <- calibrate(f, cmethod = "KM", method = "boot", u = 5, m = (nrow(test_data) / 3), B = 500)

plot(cal, xlim = c(0.95, 1.0), ylim = c(0.95, 1.0), xlab = "Nomogram-predicted OS (%)", ylab = "Observed OS (%)",lwd = 3, col = "Firebrick2")

# 8-year calibration

f <- cph(Surv(ICD10_ckd_f_time, ICD10_ckd_f) ~ sex + age + BMI + smoking + frailty.phenotype + base_diabete + base_hypertension, data = test_data, time.inc = 8)

cal <- calibrate(f, cmethod = "KM", method = "boot", u = 8, m = (nrow(test_data) / 3), B = 500)

plot(cal, xlim = c(0.95, 1.0), ylim = c(0.95, 1.0), col = "MediumSeaGreen", add = TRUE)

# 10-year calibration

f <- cph(Surv(ICD10_ckd_f_time, ICD10_ckd_f) ~ sex + age + BMI + smoking + frailty.phenotype + base_diabete + base_hypertension, data = test_data, time.inc = 10)

cal <- calibrate(f, cmethod = "KM", method = "boot", u = 10, m = (nrow(test_data) / 3), B = 500)

plot(cal, xlim = c(0.95, 1.0), ylim = c(0.95, 1.0), col = "NavyBlue", add = TRUE)

legend('bottomright', c('5-year', '8-year', '10-year'), col = c("Firebrick3", "MediumSeaGreen", "NavyBlue"), lwd = 3, bty = 'n')

dev.off()

**R packages used in analysis**

**dplyr:** Used for data manipulation, including filtering, summarizing, grouping, and transforming data.

**ggplot2:** Used for creating high-quality visualizations, including survival curves, histograms, and forest plots.

**rms:** Used for regression modeling, including Cox proportional hazards regression, restricted cubic splines (RCS), and model calibration.

**survival:** Used for survival analysis, including Cox regression, Kaplan-Meier estimation, and competing risks modeling.

**survminer:** Used for visualizing survival analysis results, such as survival curves and risk tables.

**forestploter:** Used for generating forest plots to visualize hazard ratios and confidence intervals from regression models.

**grid:** Used for arranging multiple plots and customizing graphical layouts.

**cmprsk:** Used for competing risks analysis, including the Fine-Gray model.

**mice:** Used for multiple imputation to handle missing data.

**glmnet:** Used for fitting Lasso and Elastic Net regression models for feature selection.

**lattice:** Used for creating multi-panel and complex visualizations.

**Formula:** Used for specifying statistical models using formula syntax.

**foreign:** Used for reading and writing data from SAS, SPSS, and Stata.

**timeROC:** Used for performing time-dependent ROC analysis to evaluate model performance over time.

**pROC:** Used for creating and analyzing ROC curves and calculating the AUC.

**References:**

1. Fan L, Hou XY, Liu Y, Chen S, Wang Q, Du W. Catastrophic Health Expenditure Associated With Frailty in Community-Dwelling Chinese Older Adults: A Prospective Cohort Analysis. Front Public Health. 2021;9:718910. doi:10.3389/fpubh.2021.718910

2. Levey AS, Stevens LA, Schmid CH, Zhang Y, Castro III AF, Feldman HI, et al. A new equation to estimate glomerular filtration rate. Annals of internal medicine. 2009;150:604-12.

3. Cai Y-W, Zhang H-F, Gao J-W, Cai Z-X, Cai J-W, Gao Q-Y, et al. Serum albumin and risk of incident diabetes and diabetic microvascular complications in the UK Biobank cohort. Diabetes & Metabolism. 2023;49:101472.

4. He P, Ye Z, Liu M, Li H, Zhang Y, Zhou C, et al. Association of handgrip strength and/or walking pace with incident chronic kidney disease: A UK biobank observational study. J Cachexia Sarcopenia Muscle. 2023;14:805-14. doi:10.1002/jcsm.13180

5. Sullivan LM, Massaro JM, D'Agostino Sr RB. Presentation of multivariate data for clinical use: The Framingham Study risk score functions. Statistics in medicine. 2004;23:1631-60.

6. Kivipelto M, Ngandu T, Laatikainen T, Winblad B, Soininen H, Tuomilehto J. Risk score for the prediction of dementia risk in 20 years among middle aged people: a longitudinal, population-based study. The Lancet Neurology. 2006;5:735-41.

7. D'Agostino RB, Grundy S, Sullivan LM, Wilson P, Group CRP. Validation of the Framingham coronary heart disease prediction scores: results of a multiple ethnic groups investigation. Jama. 2001;286:180-7.

| Table S1. Definition of frailty phenotype and cutoff points | | |
| --- | --- | --- |
| **Components** | **Criteria** | **Field IDs** |
| **Weight loss** | Self-reported: “Compared with one year ago, has your weight changed?”  Response: yes, lost weight = 1; other = 0;  Do not know/Prefer not to answer = missing data. | 2306 |
| **Exhaustion** | Self-reported: “Over the past two weeks, how often have you felt tired or had little energy?”  Response: more than half the days or nearly every day = 1; other =0;  Do not know/Prefer not to answer = missing data. | 2080 |
| **Low physical activity** | Quintiles of sex- and age-specific levels of total MET minutes per week derived from IPAQ.  The lowest 20% of total MET minutes per week = 1; other = 0;  No response/ Prefer not to answer = missing data. | 31,  21022,  22037,  22038,  22039  22039 |
| **Slow gait speed** | Self-reported: “How would you describe your usual walking pace?”  Response: slow = 1; other = 0;  Do not know/Prefer not to answer = missing data. | 924 |
| **Low grip strength** | Measured grip strength expressed in kg by sex- and BMI- adjusted,  cut-off points,  Cut-off points:  Men  If BMI ≤24.0 kg/m^2^ & grip strength ≤29 kg  If BMI 24.1 to 26.0 kg/m^2^ & grip strength ≤30 kg  If BMI 26.1 to 28.0 kg/m^2^ & grip strength ≤30 kg  If BMI >28.0 kg/m^2^ & grip strength ≤32 kg  Women  If BMI ≤23.0 kg/m^2^ & grip strength ≤17 kg  If BMI 23.1 to 26.0 kg/m^2^ & grip strength ≤17.3 kg  If BMI 26.1 to 29.0 kg/m^2^ & grip strength ≤18 kg  If BMI >29.0 kg/m^2^ & grip strength ≤21 kg | 31,  21001,  46, 47 |

| **Table S2. ICD 10 codes for identification of new-onset CKD** | | |
| --- | --- | --- |
|  | Description |  |
| Renal Failure/RRT | Chronic renal failure Renal  Renal Osteodystrophy  Renal tubulo-interstitial disorders in transplant rejection  Mechanical complication of vascular dialysis catheter  Kidney transplant failure and rejection  Care involving dialysis  Kidney transplant status  Dependence on renal dialysis  Misadventures to patients during kidney dialysis or other perfusion  Abnormal reaction of the patient, or of later complication during kidney dialysis | N18  N25.0  N16.5  T82.4  T86.1  Z49  Z940  Z992  Y60.2, Y61.2, Y62.2  Y841 |
| Renal complication of HTN/DM | DM with renal complication  Hypertensive renal disease  Hypertensive heart and renal disease | E102, E112, E122, E132, E142  I12  1131,1132,1139 |
| Glomerular, tubulo-interstitial disease | Recurrent and persistent haematuria  Chronic nephritic syndrome  Nephrotic syndrome  Unspecified nephritic syndrome  Isolated proteinuria with specified morphological lesion  Glomerular disorders in diseases classified elsewhere  Chronic tubulo-interstitial nephritis | N020~N027  N03  N04  N05  N06  N08  N11 |
| Other functional/structural abnormality | Unspecified contracted kidney  Small kidney of unknown cause  Ischaemia and infarction of kidney  Abnormal results of kidney function studies | N26  N27  N28.0  R94.4 |
| Congenital kidney disease | Family history of disorders of the kidney and ureter  Hereditary nephropathy, not elsewhere classified  Renal agenesis and other reduction defect of kidney  Cystic kidney disease  Other congenital malformations of kidney | Z84.1  N07  Q60  Q611~Q615  Q63 |

| **Table S3. Co-variate definitions in UKB** | | |
| --- | --- | --- |
| **Co-variate** | **Definition** | **UK Biobank field ID** (if applicable) |
| Age at recruitment | Continuous | 21022 |
| Sex | Female/Male | 31 |
| Townsend deprivation index at recruitment | Continuous | 22189 |
| Ethnic background | White, Mixed, Asian or Asian British, Black or Black British | 21000 |
| Qualifications | College or University degree, A levels/AS levels or equivalent, O levels/GCSEs or equivalent, CSEs or equivalent, NVQ Or HND or HNC or equivalent, Other professional qualifications eg: nursing, teaching | 6138 |
| Smoking status | Never, previous, current | 20116 |
| Alcohol consumption | Daily or almost daily, three or four times a week, once or twice a week, one to three times a month, special occasions only, never. | 1558 |
| Average total household income before tax | Greater than 100,000, 52,000 to 100,000, 31,000 to 51,999, 18,000 to 30,999, Less than 18,000 | 738 |
| Body mass index | Continuous, kg/m^2^ | 21001 |
| Sleep duration | Continuous, hours/day | 1160 |
| Albumin, C-reactive protein creatinine, Glycated haemoglobin (HbA1c), Creatinine, HDL cholesterol, LDL direct, Cholesterol, Triglycerides | Continuous | 30600, 30710, 30750, 30700  30760, 30780, 30690, 30870 |
| Medication for cholesterol, blood pressure or diabetes/ Medication for pain relief, constipation, heartburn | Cholesterol lowering medication, Blood pressure medication, insulin/ Aspirin, Ibuprofen (e.g. Nurofen), Paracetamol,Ranitdine (e-g- Zantac), Omeprazole (e.g. Zanprol), Laxatives (e.g. Dulcolax, Senokot) | 6177, 6154 |

| **Table S4. Co-variate definitions in CHARLS** | |
| --- | --- |
| Birth Year | ba002_1 |
| Sex | Rgender |
| BMI, kg/m^2^ | qi002: Height  ql002: Weight Measurement |
| Smoking, n (%) | da059 : Smoke or Not  da061 : Still Smoking or Not |
| Frailty phenotype, n (%) | **Slowness**  qg002 : Walking Speed Time-1  qg003 : Walking Speed Time-2  **weakness**  qc003 : Left Hand-1kg  qc004 : Right Hand-1kg  qc005 : Left Hand-2kg  qc006 : Right Hand-2kg  **Exhaustion**  dc018 : Could Not Get Going  dc012 : Felt Everything I Did Was An Eﬀort  **Inactivity**  da051 3 :Y/N Walking At Least 10 Minutes Continuously  **shrinking**  da047 : Any Weigh Change |
| Sleep time, hours/day | da049: Average Hours for One Night Sleeping Time During the Past  Month |
| Diabetes, n (%) | da0073: Diabetes or High Blood Sugar  da014s1 : Take Chinese Traditional Medicine for Diabetes  da014s2 : Take Western Morden Medicine for Diabetes  da014s3 : Taking Insulin Injections for Diabetes  da014s4 : None of the Above for Diabetes  bl_glu: Glucose (mg/dl)  bl_hbalc: Glycated Hemoglobin (%) |
| Hypertension, n (%) | da0071: Hypertension  da0081: Do You Konw If You Have Hypertension  da011s1: Take Chinese Traditional Medicine for Hypertension  da011s2: Take Western Morden Medicine for Hypertension  da011s3: None of the Above for Hypertension |
| CKD | bl_crea: Creatinine (mg/dl) |

|  | | | |
| --- | --- | --- | --- |
| Table S5. Definition and list of long-term morbidities | | | |
|  | **Morbidity grouping*** | **Conditions included** | **Code** |
| **1** | **Hypertension** | Hypertension | 1065 |
|  |  | Essential hypertension | 1072 |
| **2** | **Coronary heart disease** | Heart attack/MI | 1075 |
|  |  | Angina | 1074 |
| **3** | **Diabetes** | Diabetic nephropathy | 1607 |
|  |  | Diabetic neuropathy/ulcers | 1468 |
|  |  | Diabetes | 1220 |
|  |  | Type 1 diabetes | 1222 |
|  |  | Type 2 diabetes | 1223 |
|  |  | Diabetic eye disease | 1276 |
| **4** | **Stroke/TIA** | Stroke | 1081 |
|  |  | TIA | 1082 |
|  |  | Subarachnoid haemorrhage | 1086 |
|  |  | Brain haemorrhage | 1491 |
|  |  | Ischaemic stroke | 1583 |
| **5** | **Atrial fibrillation** | Atrial fibrillation | 1471 |
| **6** | **Heart failure** | Cardiomyopathy | 1079 |
|  |  | Hypertrophic cardiomyopathy | 1588 |
|  |  | Heart failure/pulmonary oedema | 1076 |
| **7** | **Peripheral vascular disease** | Peripheral vascular disease | 1067 |
|  |  | Leg claudication/intermittent claudication | 1087 |
| **8** | **COPD** | COPD/Chronic obstructive pulmonary disease | 1112 |
|  |  | Emphysema/Chronic bronchitis | 1113 |
|  |  | Emphysema | 1472 |
| **9** | **Asthma** | Asthma | 1111 |
| **10** | **Bronchiectasis** | Bronchiectasis | 1114 |
| **11** | **Cancer*** | “yes”/”no” to “have you ever had cancer?” |  |
| **12** | **Dyspepsia** | Gastro-oesophageal reflux (GORD) | 1138 |
|  |  | Oesophagitis/Barrett’s oesophagus | 1139 |
|  |  | Gastric stomach ulcers | 1142 |
|  |  | Gastric erosions/gastritis | 1143 |
|  |  | Duodenal ulcer | 1457 |
|  |  | Dyspepsia/indigestion | 1510 |
|  |  | Hiatus hernia | 1474 |
|  |  | Helicobacter pylori | 1442 |
| **13** | **Diverticular disease** | Diverticular disease/diverticulitis | 1458 |
| **14** | **Irritable bowel syndrome** | Irritable bowel syndrome | 1154 |
| **15** | **Chronic liver disease** | Oesophageal varices | 1141 |
|  |  | Non infective hepatitis | 1157 |
|  |  | Liver failure/cirrhosis | 1158 |
|  |  | Primary biliary cirrhosis | 1506 |
| **16** | **Inflammatory bowel disease** | Inflammatory bowel disease | 1461 |
|  |  | Crohn’s disease | 1462 |
|  |  | Ulcerative colitis | 1463 |
| **17** | **Constipation** | Constipation | 1599 |
| **18** | **Viral hepatitis** | Hepatitis B | 1579 |
|  |  | Hepatitis C | 1580 |
|  |  | Hepatitis D | 1581 |
| **19** | **Depression** | Depression | 1286 |
|  |  | Postnatal depression | 1531 |
| **20** | **Anxiety** | Anxiety/panic attacks | 1287 |
|  |  | Nervous breakdown | 1288 |
|  |  | Post-traumatic stress disorder | 1469 |
|  |  | Obsessive compulsive disorder | 1615 |
|  |  | Stress | 1614 |
|  |  | Insomnia | 1616 |
|  |  | Psychological/psychiatric problem | 1243 |
| **21** | **Schizophrenia/Bipolar affective disorder** | Schizophrenia | 1289 |
|  | **Bipolar** | Mania | 1291 |
|  |  | Bipolar disorder | 1291 |
|  |  | Manic depression | 1291 |
| **22** | **Connective tissue diseases** | Myositis/myopathy | 1322 |
|  |  | Systemic lupus erythematosus/SLE | 1381 |
|  |  | Connective tissue disorder | 1373 |
|  |  | Sjogren’s syndrome. sicca syndrome | 1382 |
|  |  | Dermato-polymyositis | 1383 |
|  |  | Scleroderma /systemic sclerosis | 1384 |
|  |  | Rheumatoid arthritis | 1464 |
|  |  | Psoriatic arthropathy | 1477 |
|  |  | Dermatomyositis | 1480 |
|  |  | Polymyositis | 1481 |
|  |  | Polymyalgia rheumatica | 1377 |
| **23** | **Painful conditions** | Back pain | 1534 |
|  |  | Joint pain | 1537 |
|  |  | Headaches (not migraine) | 1436 |
|  |  | Sciatica | 1476 |
|  |  | Plantar fasciitis | 1540 |
|  |  | Carpal tunnel syndrome | 1541 |
|  |  | Fibromyalgia | 1542 |
|  |  | Arthritis | 1538 |
|  |  | Shingles | 1573 |
|  |  | Disc problem | 1532 |
|  |  | Prolapsed disc/slipped disc | 1312 |
|  |  | Spine arthritis/spondylitis | 1311 |
|  |  | Ankylosing spondylitis | 1313 |
|  |  | Back problem | 1294 |
|  |  | Osteoarthritis | 1465 |
|  |  | Gout | 1466 |
|  |  | Cervical spondylosis | 1478 |
|  |  | Trigeminal neuralgia | 1523 |
|  |  | Disc degeneration | 1533 |
|  |  | Trapped nerve/compressed nerve | 1257 |
| **24** | **Osteoporosis** | Osteoporosis | 1309 |
| **25** | **Thyroid disorders** | Thyroid problem (not cancer） | 1224 |
|  |  | Hyperthroidism/thyrotoxicosis | 1225 |
|  |  | Hypothyroidism/myxoedema | 1226 |
|  |  | Grave’s disease | 1522 |
|  |  | Thyroid goitre | 1610 |
|  |  | Thyroiditis | 1428 |
| **26** | **Alcohol problems** | Alcohol dependency | 1408 |
|  |  | Alcoholic liver disease/alcoholic cirrhosis | 1604 |
| **27** | **Chronic kidney disease** | Polycystic kidney | 1427 |
|  |  | Diabetic nephropathy | 1607 |
|  |  | Renal/kidney failure | 1192 |
|  |  | Renal failure requiring dialysis | 1193 |
|  |  | Renal failure not requiring dialysis | 1194 |
|  |  | Kidney nephropathy | 1519 |
|  |  | Immunoglobulin A (IgA) nephropathy | 1520 |
| **28** | **Prostate disorders** | Prostate problem (not cancer) | 1207 |
|  |  | Enlarged prostate | 1396 |
|  |  | Benign prostatic hypertrophy | 1516 |
| **29** | **Glaucoma** | Glaucoma | 1277 |
| **30** | **Epilepsy** | Epilepsy | 1264 |
| **31** | **Dementia** | Dementia/Alzheimer/cognitive impairment | 1263 |
| **32** | **Psoriasis or eczema** | Eczema/dermatitis | 1452 |
|  |  | Psoriasis | 1453 |
| **33** | **Migraine** | Migraine | 1265 |
| **34** | **Chronic sinusitis** | Chronic sinusitis | 1416 |
| **35** | **Anorexia or bulimia** | Anorexia, bulimia/other eating disorder | 1470 |
| **36** | **Parkinson’s disease** | Parkinson’s disease | 1262 |
| **37** | **Multiple sclerosis** | Multiple sclerosis | 1261 |
| **38** | **Chronic fatigue syndrome** | Chronic fatigue syndrome | 1482 |
| **39** | **Endometriosis** | Endometriosis | 1402 |
| **40** | **Meniere disease** | Meniere disease | 1421 |
| **41** | **Pernicious anaemia** | Pernicious anaemia | 1331 |
| **42** | **Polycystic ovaries** | Polycystic ovaries | 1350 |
| *Self-report lifetime diagnosis by doctor recorded by interview (UK Biobank data field ID: 20002), except cancer diagnosis which was reported by touch-screen questionnaire. The list of disease groupings was based on Barnett *et al* (2012). In the present study, we did not include patients with chronic kidney disease at baseline. | | | |

| **Table S6. Variants used to make the genetic risk score for eGFR (using creatinine in the CKD-EPI equation)** | | | | | |
| --- | --- | --- | --- | --- | --- |
| RSID | Chromosome and base pair | Effect allele | Non-effect allele | Beta | EAF |
| rs74748843 | 1:10730910 | T | C | -0.0048 | 0.0707 |
| rs10159261 | 1:15912987 | T | G | -0.0034 | 0.3599 |
| rs12061708 | 1:18809916 | A | G | -0.0026 | 0.2936 |
| rs2749153 | 1:23699340 | A | G | -0.0033 | 0.6894 |
| rs659437 | 1:46037394 | T | C | -0.0027 | 0.2184 |
| rs11211257 | 1:46581933 | A | G | 0.0027 | 0.819 |
| rs688540 | 1:48002447 | A | G | -0.003 | 0.8673 |
| rs17413465 | 1:55718708 | A | C | 0.0025 | 0.1824 |
| rs1757915 | 1:56615809 | A | G | 0.0021 | 0.3254 |
| rs7536433 | 1:78023173 | T | C | 0.0021 | 0.2632 |
| rs679843 | 1:78707493 | T | C | 0.0021 | 0.334 |
| rs1887252 | 1:82957871 | C | G | -0.0019 | 0.6173 |
| rs7543734 | 1:94050911 | C | G | 0.0031 | 0.2056 |
| rs11166440 | 1:100808363 | A | G | 0.002 | 0.6026 |
| rs10857788 | 1:110012289 | A | G | 0.003 | 0.7001 |
| rs12736457 | 1:113258293 | C | G | 0.0054 | 0.8711 |
| rs3118119 | 1:150159616 | T | C | 0.003 | 0.1799 |
| rs267738 | 1:150940625 | T | G | -0.0048 | 0.7985 |
| rs4971100 | 1:155155731 | A | G | 0.002 | 0.4821 |
| rs3845534 | 1:163738950 | A | G | -0.0019 | 0.5336 |
| rs4656220 | 1:170649277 | T | C | 0.002 | 0.4224 |
| rs1011731 | 1:172346548 | A | G | -0.0019 | 0.5961 |
| rs3795503 | 1:180905694 | T | C | 0.002 | 0.3598 |
| rs78444298 | 1:184672098 | A | G | -0.0105 | 0.0185 |
| rs78329830 | 1:186769572 | A | G | -0.0054 | 0.9601 |
| rs3850625 | 1:201016296 | A | G | 0.0046 | 0.1159 |
| rs75625374 | 1:208039431 | C | G | 0.0045 | 0.0582 |
| rs7535253 | 1:214744893 | T | C | 0.0021 | 0.2683 |
| rs2577134 | 1:220224321 | T | C | 0.002 | 0.6773 |
| rs61830291 | 1:221001142 | A | C | -0.0036 | 0.8991 |
| rs417237 | 1:228532195 | T | G | 0.0018 | 0.5671 |
| rs2490391 | 1:243469669 | A | C | -0.0024 | 0.4324 |
| rs3791221 | 2:226933 | A | G | 0.0022 | 0.6714 |
| rs807624 | 2:15782471 | T | G | 0.0032 | 0.4216 |
| rs4491726 | 2:18676276 | A | G | 0.0032 | 0.6934 |
| rs780093 | 2:27742603 | T | C | 0.0044 | 0.4171 |
| rs2301343 | 2:40680149 | T | G | -0.0023 | 0.7621 |
| rs10865189 | 2:43433257 | C | G | 0.0024 | 0.5126 |
| rs2971880 | 2:54885640 | A | T | -0.0024 | 0.3728 |
| rs10197255 | 2:67874553 | A | T | 0.0018 | 0.4045 |
| rs6546869 | 2:73895765 | A | G | 0.0059 | 0.2289 |
| rs11123169 | 2:113967075 | T | C | 0.0025 | 0.6769 |
| rs17050272 | 2:121306440 | A | G | -0.0022 | 0.4351 |
| rs11694902 | 2:121988884 | A | G | 0.0041 | 0.1379 |
| rs7425436 | 2:148759656 | A | G | 0.0024 | 0.6458 |
| rs4664475 | 2:152387553 | T | C | -0.002 | 0.378 |
| rs35472707 | 2:169995581 | T | C | -0.0073 | 0.0501 |
| rs187355703 | 2:176993583 | C | G | 0.01 | 0.9741 |
| rs35284526 | 2:178121524 | A | C | 0.0029 | 0.3237 |
| rs4666821 | 2:183077254 | T | G | 0.002 | 0.5295 |
| rs60980181 | 2:188168567 | A | T | -0.0027 | 0.165 |
| rs1047891 | 2:211540507 | A | C | -0.0065 | 0.2927 |
| rs1548945 | 2:217665788 | T | C | 0.0036 | 0.4389 |
| rs1050816 | 2:220358198 | T | C | 0.0026 | 0.333 |
| rs35669853 | 2:227287718 | A | G | 0.0024 | 0.1823 |
| rs13003198 | 2:234257105 | T | C | 0.0018 | 0.3693 |
| rs795009 | 3:12208671 | T | G | 0.002 | 0.7306 |
| rs6778731 | 3:13947504 | T | C | -0.0017 | 0.589 |
| rs6779998 | 3:30749965 | A | G | -0.0017 | 0.5174 |
| rs11914389 | 3:38527215 | T | C | 0.003 | 0.4815 |
| rs7651407 | 3:48443816 | T | C | 0.0025 | 0.4445 |
| rs4625 | 3:49572140 | A | G | -0.0023 | 0.6992 |
| rs2581820 | 3:53020544 | A | G | 0.0021 | 0.2864 |
| rs3774726 | 3:63974477 | T | C | -0.0021 | 0.363 |
| rs2289746 | 3:105455955 | T | C | -0.0019 | 0.4096 |
| rs9868185 | 3:121657593 | A | G | 0.0026 | 0.5016 |
| rs10934754 | 3:125906237 | T | C | 0.002 | 0.6039 |
| rs35320690 | 3:135932494 | T | C | -0.0025 | 0.7258 |
| rs9828976 | 3:136536835 | C | G | -0.0024 | 0.7558 |
| rs7624084 | 3:141093285 | T | C | 0.0017 | 0.5767 |
| rs1397764 | 3:141750810 | A | G | 0.0043 | 0.274 |
| rs76272256 | 3:168888112 | T | C | 0.0024 | 0.2426 |
| rs56065557 | 3:185354216 | C | G | -0.0029 | 0.3244 |
| rs11919484 | 3:186432839 | T | G | -0.0026 | 0.3165 |
| rs9823161 | 3:193811168 | A | G | 0.0022 | 0.577 |
| rs75501914 | 4:3449781 | A | G | 0.0039 | 0.0941 |
| rs3775932 | 4:10090930 | A | C | -0.0018 | 0.5077 |
| rs16874073 | 4:23743962 | T | C | -0.0045 | 0.9474 |
| rs4864890 | 4:52686513 | T | C | -0.0023 | 0.3064 |
| rs28817415 | 4:77401452 | T | C | -0.0073 | 0.4037 |
| rs12509595 | 4:81182554 | T | C | -0.0035 | 0.7001 |
| rs223471 | 4:103698786 | C | G | 0.0028 | 0.3408 |
| rs71606723 | 4:115498457 | A | T | 0.0025 | 0.7716 |
| rs13159523 | 5:676962 | A | G | -0.0024 | 0.4942 |
| rs13157326 | 5:34504277 | A | G | -0.0027 | 0.4493 |
| rs1362800 | 5:39378115 | T | C | -0.0049 | 0.3832 |
| rs495237 | 5:39950266 | T | G | 0.0027 | 0.2487 |
| rs11746506 | 5:44812566 | T | C | 0.0017 | 0.4088 |
| rs12520984 | 5:52787358 | C | G | 0.0019 | 0.3224 |
| rs79760705 | 5:53298716 | T | G | 0.0056 | 0.1076 |
| rs72759880 | 5:67750213 | T | G | -0.0056 | 0.1079 |
| rs2010352 | 5:68656327 | A | G | -0.0018 | 0.4534 |
| rs3797537 | 5:78322650 | A | G | 0.0019 | 0.7284 |
| rs12777 | 5:131671662 | C | G | 0.005 | 0.9586 |
| rs12163971 | 5:132226669 | A | C | -0.0029 | 0.1573 |
| rs11743174 | 5:148524820 | T | C | 0.0019 | 0.6671 |
| rs3812036 | 5:176813404 | T | C | -0.0065 | 0.2581 |
| rs11755724 | 6:7118990 | A | G | 0.0027 | 0.3721 |
| rs3765502 | 6:24354045 | T | C | 0.0024 | 0.7916 |
| rs144100226 | 6:34180297 | T | C | 0.0059 | 0.0367 |
| rs13200335 | 6:41690823 | A | C | 0.0024 | 0.4626 |
| rs77915916 | 6:43287722 | A | T | 0.0046 | 0.917 |
| rs881858 | 6:43806609 | A | G | -0.0054 | 0.7138 |
| rs720989 | 6:44765535 | T | G | 0.0021 | 0.7879 |
| rs12212034 | 6:51492862 | T | C | -0.0018 | 0.3743 |
| rs6458868 | 6:52630153 | T | C | -0.002 | 0.6741 |
| rs3925003 | 6:55422618 | T | C | -0.0018 | 0.5804 |
| rs72912510 | 6:90118764 | A | G | -0.0024 | 0.1958 |
| rs1857859 | 6:100894587 | A | G | 0.0019 | 0.3107 |
| rs1268168 | 6:109008158 | A | G | 0.0024 | 0.3375 |
| rs7740107 | 6:130374461 | A | T | 0.0027 | 0.7445 |
| rs9375818 | 6:131882078 | A | G | -0.0031 | 0.2545 |
| rs3822939 | 6:133849789 | A | G | -0.0025 | 0.4222 |
| rs9397738 | 6:154986664 | A | G | 0.0027 | 0.838 |
| rs12207180 | 6:160633107 | A | T | -0.0085 | 0.113 |
| rs62435145 | 7:1286567 | T | G | -0.006 | 0.5913 |
| rs6968554 | 7:17287106 | A | G | -0.0019 | 0.4256 |
| rs3750081 | 7:32930876 | T | G | -0.0022 | 0.5931 |
| rs700753 | 7:46753684 | C | G | 0.0031 | 0.3166 |
| rs55773927 | 7:65337902 | T | C | 0.0019 | 0.41 |
| rs801193 | 7:66030612 | T | G | -0.002 | 0.5758 |
| rs41301394 | 7:75612803 | T | C | 0.0023 | 0.3176 |
| rs6973656 | 7:77422583 | A | G | 0.0035 | 0.6359 |
| rs35154268 | 7:127505755 | A | C | -0.0022 | 0.7187 |
| rs3757387 | 7:128576086 | T | C | 0.003 | 0.5895 |
| rs62491533 | 7:129564134 | T | C | -0.0027 | 0.8097 |
| rs10254101 | 7:151415536 | T | C | -0.0068 | 0.2768 |
| rs12671694 | 7:155665959 | T | C | 0.0025 | 0.5376 |
| rs868822 | 7:156252939 | T | G | 0.0029 | 0.3331 |
| rs2980423 | 8:8142575 | T | C | -0.0023 | 0.4891 |
| rs1533059 | 8:8684953 | A | G | 0.0025 | 0.5142 |
| rs35353426 | 8:9297246 | T | C | -0.0026 | 0.2916 |
| rs7832708 | 8:10190040 | T | C | 0.0022 | 0.4947 |
| rs11783418 | 8:10841858 | A | G | -0.002 | 0.4997 |
| rs10098664 | 8:11417493 | T | C | -0.0021 | 0.4859 |
| rs34861762 | 8:23748420 | T | C | -0.0043 | 0.3863 |
| rs10102889 | 8:32435620 | C | G | -0.0036 | 0.509 |
| rs2976178 | 8:87332552 | C | G | -0.0025 | 0.6717 |
| rs2954017 | 8:126476873 | T | C | 0.0024 | 0.4575 |
| rs12377027 | 9:20554583 | A | G | -0.0026 | 0.8182 |
| rs13287724 | 9:33169034 | A | T | -0.003 | 0.8913 |
| rs544169 | 9:33956791 | A | G | 0.0022 | 0.7305 |
| rs2039424 | 9:71432174 | A | G | 0.0044 | 0.6356 |
| rs1321917 | 9:119324929 | C | G | -0.0023 | 0.4364 |
| rs7024579 | 9:139100413 | T | C | 0.0023 | 0.2889 |
| rs28404308 | 9:140103272 | A | T | 0.0024 | 0.6437 |
| rs80282103 | 10:899071 | A | T | 0.0078 | 0.9143 |
| rs6481598 | 10:29781798 | C | G | 0.0024 | 0.7934 |
| rs7072591 | 10:35150364 | A | G | 0.0019 | 0.555 |
| rs10821905 | 10:52646093 | A | G | 0.0037 | 0.1845 |
| rs10821944 | 10:63785089 | T | G | 0.002 | 0.7045 |
| rs7475348 | 10:69965177 | T | C | 0.0031 | 0.4595 |
| rs12240572 | 10:75016365 | A | T | -0.0032 | 0.1671 |
| rs816850 | 10:79252446 | C | G | -0.002 | 0.2532 |
| rs9420446 | 10:88880689 | T | C | 0.0023 | 0.2679 |
| rs2068888 | 10:94839642 | A | G | -0.0024 | 0.4907 |
| rs4918943 | 10:97278922 | A | G | -0.0022 | 0.2185 |
| rs284859 | 10:104573017 | T | G | 0.0026 | 0.2072 |
| rs1536225 | 10:105202318 | T | G | -0.0021 | 0.6328 |
| rs1055256 | 10:126446592 | A | G | 0.0025 | 0.4235 |
| rs11564722 | 11:2178330 | T | C | 0.0033 | 0.311 |
| rs63934 | 11:2789062 | A | G | 0.0041 | 0.8265 |
| rs1541937 | 11:5578558 | A | C | -0.0029 | 0.6395 |
| rs963837 | 11:30749090 | T | C | -0.0057 | 0.5728 |
| rs6484504 | 11:31424823 | T | C | -0.0026 | 0.3053 |
| rs61897431 | 11:47427667 | T | C | 0.0029 | 0.6472 |
| rs7127946 | 11:48250675 | T | C | 0.0023 | 0.6948 |
| rs2727040 | 11:49057603 | T | C | -0.0026 | 0.2382 |
| rs1813937 | 11:50468801 | T | C | 0.0022 | 0.7185 |
| rs1783827 | 11:57409538 | A | G | -0.002 | 0.6021 |
| rs948493 | 11:65552154 | T | C | -0.0033 | 0.3306 |
| rs3892895 | 11:68884755 | A | G | -0.0023 | 0.3993 |
| rs11237450 | 11:78023356 | A | C | 0.0032 | 0.254 |
| rs6589750 | 11:119326726 | A | G | 0.002 | 0.6315 |
| rs10790452 | 11:121584931 | T | C | 0.002 | 0.7369 |
| rs11062167 | 12:364739 | A | G | -0.0039 | 0.4834 |
| rs632887 | 12:3392351 | A | G | 0.0032 | 0.6157 |
| rs4238020 | 12:4616642 | T | C | 0.0029 | 0.8703 |
| rs117113238 | 12:12209203 | A | G | 0.0039 | 0.0939 |
| rs10846157 | 12:15325031 | A | C | -0.0034 | 0.7602 |
| rs2634675 | 12:48740855 | A | G | 0.0025 | 0.52 |
| rs12313306 | 12:57751854 | T | C | 0.0029 | 0.229 |
| rs1275609 | 12:76271183 | A | G | 0.0024 | 0.3862 |
| rs41284816 | 13:50655989 | T | G | -0.0078 | 0.0262 |
| rs500830 | 13:72348768 | T | C | 0.0029 | 0.4603 |
| rs72683923 | 14:50735947 | T | C | -0.0074 | 0.9791 |
| rs6574652 | 14:81870100 | T | C | -0.0017 | 0.5016 |
| rs1028455 | 14:88829975 | A | T | 0.002 | 0.3273 |
| rs17184313 | 14:93102251 | T | C | -0.0029 | 0.1744 |
| rs61993680 | 14:100752644 | A | C | -0.0019 | 0.6082 |
| rs12913015 | 15:39305443 | T | C | 0.0027 | 0.4148 |
| rs6492982 | 15:41399951 | T | C | -0.0033 | 0.5569 |
| rs1145077 | 15:45683795 | T | G | -0.0085 | 0.4206 |
| rs690428 | 15:53950578 | A | C | -0.0039 | 0.6653 |
| rs1994887 | 15:57793765 | A | C | -0.002 | 0.257 |
| rs956006 | 15:62808539 | T | C | 0.0019 | 0.3309 |
| rs11071738 | 15:63580155 | T | C | -0.0025 | 0.5726 |
| rs11071939 | 15:67463391 | T | C | -0.0039 | 0.9286 |
| rs351237 | 15:74477239 | A | G | -0.0018 | 0.62 |
| rs2472297 | 15:75027880 | T | C | 0.0039 | 0.2567 |
| rs4886696 | 15:75664570 | A | T | -0.0032 | 0.333 |
| rs4886755 | 15:76298132 | A | G | 0.0041 | 0.4971 |
| rs166906 | 15:76802175 | T | C | 0.0033 | 0.0902 |
| rs17507300 | 15:83722059 | A | G | 0.0024 | 0.8319 |
| rs59646751 | 15:99276521 | T | G | -0.0023 | 0.3043 |
| rs438339 | 16:2003425 | T | C | 0.0035 | 0.8818 |
| rs1635404 | 16:3747042 | T | G | -0.0025 | 0.7073 |
| rs193538 | 16:16127916 | T | G | -0.002 | 0.6993 |
| rs77924615 | 16:20392332 | A | G | 0.0098 | 0.2033 |
| rs9932625 | 16:51735746 | A | G | -0.003 | 0.2566 |
| rs7203398 | 16:53189672 | A | C | 0.0025 | 0.7412 |
| rs7185391 | 16:68323115 | T | G | -0.0027 | 0.2735 |
| rs62050038 | 16:69802865 | A | T | 0.0028 | 0.8257 |
| rs62053077 | 16:71643669 | T | G | -0.0021 | 0.4289 |
| rs1858800 | 16:73024276 | T | C | 0.002 | 0.3219 |
| rs28581385 | 16:79942679 | A | T | -0.0028 | 0.8358 |
| rs154656 | 16:89708003 | A | T | -0.003 | 0.4269 |
| rs28735420 | 17:12139964 | T | G | 0.0039 | 0.9209 |
| rs2349648 | 17:17017267 | T | G | -0.0017 | 0.3387 |
| rs9891340 | 17:17543846 | T | C | 0.0024 | 0.5517 |
| rs2440165 | 17:19428719 | T | C | 0.004 | 0.6364 |
| rs2411192 | 17:34882998 | A | T | -0.0024 | 0.596 |
| rs4794813 | 17:37670994 | A | T | 0.0055 | 0.2439 |
| rs227731 | 17:54773238 | T | G | 0.0018 | 0.5672 |
| rs35662455 | 17:56755223 | C | G | 0.003 | 0.8855 |
| rs9903801 | 17:58915261 | C | G | 0.0047 | 0.1554 |
| rs9895661 | 17:59456589 | T | C | 0.0069 | 0.7223 |
| rs8866 | 17:65373979 | C | G | -0.0018 | 0.622 |
| rs883541 | 17:66449122 | A | G | -0.0022 | 0.7176 |
| rs1719934 | 18:5585158 | A | G | 0.0026 | 0.5752 |
| rs16942751 | 18:24393213 | A | C | -0.0029 | 0.1821 |
| rs4940525 | 18:59354616 | T | C | 0.0025 | 0.2603 |
| rs2974751 | 19:13053034 | A | C | 0.0018 | 0.376 |
| rs8101667 | 19:33402419 | T | C | 0.0044 | 0.3933 |
| rs7251730 | 19:36997147 | T | C | 0.0024 | 0.3452 |
| rs78241494 | 19:37649748 | T | C | -0.003 | 0.6965 |
| rs113445505 | 19:38157969 | T | C | 0.0037 | 0.3564 |
| rs281380 | 19:49214470 | T | C | -0.0021 | 0.5628 |
| rs34647824 | 19:50138143 | A | C | -0.0021 | 0.7386 |
| rs62187537 | 20:1333060 | T | C | 0.0039 | 0.0684 |
| rs1509117 | 20:8303120 | A | T | 0.0024 | 0.3018 |
| rs1041606 | 20:14677788 | T | C | -0.0021 | 0.2319 |
| rs6087579 | 20:32985155 | A | G | -0.0028 | 0.4769 |
| rs2273684 | 20:33529766 | T | G | 0.0032 | 0.5756 |
| rs17216707 | 20:52732362 | T | C | -0.0051 | 0.8134 |
| rs2235826 | 20:56143169 | A | T | -0.003 | 0.7932 |
| rs1407040 | 20:57472174 | T | C | 0.0018 | 0.6756 |
| rs35636653 | 20:60858758 | T | C | 0.0022 | 0.3369 |
| rs72629024 | 20:62152519 | C | G | 0.0035 | 0.6634 |
| rs4408777 | 20:62706105 | A | G | -0.0021 | 0.5139 |
| rs2823139 | 21:16576783 | A | G | -0.0026 | 0.3274 |
| rs2834317 | 21:35356706 | A | G | -0.0035 | 0.1425 |
| rs2244237 | 21:37818141 | T | G | 0.0027 | 0.2191 |
| rs131263 | 22:30133045 | T | C | 0.0024 | 0.4584 |
| rs80576 | 22:36539804 | A | G | -0.0028 | 0.1592 |
| rs4820324 | 22:38599857 | C | G | -0.0023 | 0.5902 |
| rs112880707 | 22:40884662 | T | C | 0.0052 | 0.1713 |
| rs738527 | 22:43112961 | T | C | 0.0032 | 0.2917 |

| | Table S7. Risk of incident CKD according to frailty phenotype stratified by diabetes | | | | | | | --- | --- | --- | --- | --- | --- | | Diabetes at baseline | Non-frailty | Prefrailty | | Frailty | | |  | HR (95 % CI) | HR (95 % CI) | P value | HR (95 % CI) | *P* value | | Yes | 1.00 (Ref.) | 1.134 (0.992-1.297) | 0.066 | 1.572 (1.314-1.881) | <0.001 | | NO | 1.00 (Ref.) | 1.141 (1.084-1.201) | <0.001 | 1.603 (1.451-1.772) | <0.001 | |
| --- | --- | --- | --- | --- | --- | --- | --- | --- | --- | --- | --- | --- | --- | --- | --- | --- | --- | --- | --- | --- | --- | --- | --- | --- | --- | --- | --- | --- | --- | --- |

The results were adjusted for age, sex, ethnicity, education, Townsend deprivation index, average household income, BMI, smoking status, alcohol frequency intake, sleep time, C-reaction protein, eGFR, albumin, high density lipoprotein, low density lipoprotein, triglyceride, total cholesterol, HbA1c, the number of long-term morbidities, history of medication for cholesterol lowering, insulin, antihypertensive and NSAIDS, PRS. Ref indicates reference.

| Table S8. Risk of incident CKD according to diabetes stratified by different levels of frailty phenotype | | | | | | |
| --- | --- | --- | --- | --- | --- | --- |
|  | Non-frailty | | Prefrailty | | Frailty | |
| Diabetes at baseline | HR (95 % CI) | P value | HR (95 % CI) | P value | HR (95 % CI) | *P* value |
| NO | 1.00 (Ref.) |  | 1.00 (Ref.) |  | 1.00 (Ref.) |  |
| Yes | 1.186 (1.018-1.383) | 0.029 | 1.288 (1.154-1.438) | <0.001 | 1.423 (1.159-1.746) | <0.001 |

The results were adjusted for age, sex, ethnicity, education, Townsend deprivation index, average household income, BMI, smoking status, alcohol frequency intake, sleep time, C-reaction protein, eGFR, albumin, high density lipoprotein, low density lipoprotein, triglyceride, total cholesterol, HbA1c, the number of long-term morbidities, history of medication for cholesterol lowering, insulin, antihypertensive and NSAIDS, PRS. Ref indicates reference.

| Table S9. Risk of incident CKD according to frailty phenotype stratified by hypertension | | | | | |
| --- | --- | --- | --- | --- | --- |
| Hypertension at baseline | Non-frailty | Prefrailty | | Frailty | |
|  | HR (95 % CI) | HR (95 % CI) | P value | HR (95 % CI) | *P* value |
| Yes | 1.00 (Ref.) | 1.127 (1.062-1.196) | <0.001 | 1.689 (1.527-1.869) | <0.001 |
| NO | 1.00 (Ref.) | 1.184 (1.093-1.283) | <0.001 | 1.449 (1.229-1.707) | <0.001 |

The results were adjusted for age, sex, ethnicity, education, Townsend deprivation index, average household income, BMI, smoking status, alcohol frequency intake, sleep time, C-reaction protein, eGFR, albumin, high density lipoprotein, low density lipoprotein, triglyceride, total cholesterol, HbA1c, the number of long-term morbidities, history of medication for cholesterol lowering, insulin, antihypertensive and NSAIDS, PRS. Ref indicates reference.

| Table S10. Risk of incident CKD according to hypertension stratified by different levels of frailty phenotype | | | | | | |
| --- | --- | --- | --- | --- | --- | --- |
|  | Non-frailty | | Prefrailty | | Frailty | |
| Hypertension at baseline | HR (95 % CI) | P value | HR (95 % CI) | P value | HR (95 % CI) | *P* value |
| NO | 1.00 (Ref.) |  | 1.00 (Ref.) |  | 1.00 (Ref.) |  |
| Yes | 1.289 (1.191-1.395) | <0.001 | 1.230 (1.146-1.320) | <0.001 | 1.597 (1.342-1.900) | <0.001 |

The results were adjusted for age, sex, ethnicity, education, Townsend deprivation index, average household income, BMI, smoking status, alcohol frequency intake, sleep time, C-reaction protein, eGFR, albumin, high density lipoprotein, low density lipoprotein, triglyceride, total cholesterol, HbA1c, the number of long-term morbidities, history of medication for cholesterol lowering, insulin, antihypertensive and NSAIDS, PRS. Ref indicates reference.

| Table S11. Associations of frailty phenotype with the incidence of new-onset CKD without multiple imputation | | | | | |
| --- | --- | --- | --- | --- | --- |
|  | Non-frailty | Prefrailty | Frailty | P for Trend | Per 1-point increase |
| Total case | 1978 | 2251 | 368 |  |  |
| Total simple size | 56943 | 46480 | 3278 |  |  |
| Unadjusted model, HR (95%CI) | 1.00 (Ref.) | 1.432 (1.348-1.522) | 3.732 (3.339-4.172) | <0.001 | 1.456 (1.414-1.501) |
| Multivariate model, HR (95%CI) | 1.00 (Ref.) | 1.123 (1.055-1.195) | 1.531 (1.356-1.728) | <0.001 | 1.129 (1.093-1.167) |

The multivariate model adjusted for age, sex, ethnicity, education, Townsend deprivation index, average household income, BMI, smoking status, alcohol frequency intake, sleep time, C-reaction, eGFR, protein, albumin, high density lipoprotein, low density lipoprotein, triglyceride, total cholesterol, HbA1c, the number of long-term morbidities, history of medication for cholesterol lowering, insulin, antihypertensive and NSAIDS, PRS. Ref indicates reference.

| Table S12. Associations of frailty phenotype with the incidence of new-onset CKD ≥2 years from the baseline | | | | | |
| --- | --- | --- | --- | --- | --- |
|  | Non-frailty | Prefrailty | Frailty | P for Trend | Per 1-point increase |
| Total case | 3050 | 3905 | 742 |  |  |
| Total simple size | 108867 | 96344 | 8110 |  |  |
| Unadjusted model, HR (95%CI) | 1.00 (Ref.) | 1.475 (1.407-1.547) | 3.567 (3.292-3.865) | <0.001 | 1.447 (1.416-1.479) |
| Multivariate model, HR (95%CI) | 1.00 (Ref.) | 1.131 (1.077-1.187) | 1.525 (1.396-1.666) | <0.001 | 1.130 (1.103-1.157) |

The multivariate model adjusted for age, sex, ethnicity, education, Townsend deprivation index, average household income, BMI, smoking status, alcohol frequency intake, sleep time, C-reaction, eGFR, protein, albumin, high density lipoprotein, low density lipoprotein, triglyceride, total cholesterol, HbA1c, the number of long-term morbidities, history of medication for cholesterol lowering, insulin, antihypertensive and NSAIDS, PRS. Ref indicates reference.

| **Table S13. Competing risk of associations of frailty phenotype with the incidence of new-onset CKD** | | | | | |
| --- | --- | --- | --- | --- | --- |
|  | Non-frailty | Prefrailty | Frailty | P for Trend | Per 1-point increase |
| Total case | 3184 | 4095 | 800 |  |  |
| Total simple size | 109290 | 96941 | 8271 |  |  |
| Unadjusted model, HR (95%CI) | 1.00 (Ref.) | 1.470 (1.400-1.540) | 3.480 (3.22-3.77) | <0.001 | 1.430 (1.400-1.470) |
| Multivariate model, HR (95%CI) | 1.00 (Ref.) | 1.132 (1.079-1.187) | 1.519 (1.392- 1.657) | <0.001 | 1.125 (1.098-1.152) |

The multivariate model adjusted for age, sex, ethnicity, education, Townsend deprivation index, average household income, BMI, smoking status, alcohol frequency intake, sleep time, C-reaction, eGFR, protein, albumin, high density lipoprotein, low density lipoprotein, triglyceride, total cholesterol, HbA1c, the number of long-term morbidities, history of medication for cholesterol lowering, insulin, antihypertensive and NSAIDS, PRS. Ref indicates reference.

|  | | | | | |
| --- | --- | --- | --- | --- | --- |
| **Table S14. Association between frailty phenotype and incident CKD in a population excluding baseline CKD defined by ICD codes** | | | | | |
|  | Non-frailty | Prefrailty | Frailty | P for Trend | Per 1-point increase |
| Total case | 3791 | 5220 | 1080 |  |  |
| Total simple size | 110186 | 97493 | 8081 |  |  |
| Unadjusted model, HR (95%CI) | 1.00 (Ref.) | 1.565 (1.501-1.632) | 3.984 (3.724-4.263) | <0.001 | 1.502 (1.474-1.530) |
| Multivariate model, HR (95%CI) | 1.00 (Ref.) | 1.176 (1.126-1.228) | 1.626 (1.509- 1.752) | <0.001 | 1.143 (1.120-1.167) |

The multivariate model adjusted for age, sex, ethnicity, education, Townsend deprivation index, average household income, BMI, smoking status, alcohol frequency intake, sleep time, C-reaction, eGFR, protein, albumin, high density lipoprotein, low density lipoprotein, triglyceride, total cholesterol, HbA1c, the number of long-term morbidities, history of medication for cholesterol lowering, insulin, antihypertensive and NSAIDS, PRS. Ref indicates reference.

|  | | | | |
| --- | --- | --- | --- | --- |
| **Table S15. Individual components of frailty phenotype and their association with incident CKD** **in a population excluding baseline CKD defined by ICD codes** | | | | |
| Characteristic | HR (95%CI) | *P* value | HR (95%CI) | *P* value |
| Frailty component | Unadjusted model |  | Multivariate model |  |
| Weight loss | 1.502 (1.474-1.530) | <0.001 | 1.100 (1.044-1.158) | <0.001 |
| Exhaustion | 1.541 (1.461-1.626) | <0.001 | 1.163 (1.099-1.231) | <0.001 |
| Low physical activity | 1.318 (1.260-1.379) | <0.001 | 1.059 (1.010-1.110) | 0.019 |
| Slow gait speed | 3.412 (3.240-3.594) | <0.001 | 1.362 (1.283-1.446) | <0.001 |
| Low grip strength | 2.102 (2.014-2.195) | <0.001 | 1.135 (1.084-1.188) | <0.001 |

The multivariate model adjusted for age, sex, ethnicity, education, Townsend deprivation index, average household income, BMI, smoking status, alcohol frequency intake, sleep time, eGFR, C-reaction protein, albumin, high density lipoprotein, low density lipoprotein, triglyceride, total cholesterol, HbA1c, the number of long-term morbidities, history of medication for cholesterol lowering, insulin, antihypertensive and NSAIDS, PRS and five individual components when these were not the exposure.

| **Table S16.** **Associations of frailty phenotype with the incidence of new-onset CKD after excluding participants with baseline eGFR <75 mL/min/1.73m²** | | | | | |
| --- | --- | --- | --- | --- | --- |
|  | Non-frailty | Prefrailty | Frailty | P for Trend | Per 1-point increase |
| Total case | 1513 | 1903 | 352 |  |  |
| Total simple size | 56939 | 49409 | 3808 |  |  |
| Unadjusted model, HR (95%CI) | 1.00 (Ref.) | 1.478 (1.381-1.581) | 3.816 (3.398-4.285) | <0.001 | 1.455 (1.424-1.486) |
| Multivariate model, HR (95%CI) | 1.00 (Ref.) | 1.145 (1.068-1.228) | 1.629 (1.433-1.852) | <0.001 | 1.142 (1.116-1.170) |

The multivariate model adjusted for age, sex, ethnicity, education, Townsend deprivation index, average household income, BMI, smoking status, alcohol frequency intake, sleep time, eGFR, C-reaction protein, albumin, high density lipoprotein, low density lipoprotein, triglyceride, total cholesterol, HbA1c, the number of long-term morbidities, history of medication for cholesterol lowering, insulin, antihypertensive and NSAIDS, PRS. Ref indicates reference.

| **Table S17.** **Individual components of frailty phenotype and their association with incident CKD after excluding participants with baseline eGFR <75 mL/min/1.73m²** | | | | |
| --- | --- | --- | --- | --- |
| Characteristic | HR (95%CI) | P value | HR (95%CI) | P value |
| Frailty component | Unadjusted model |  | Multivariate model |  |
| Weight loss | 1.191 (1.094-1.295) | <0.001 | 1.101 (1.011-1.199) | 0.028 |
| Exhaustion | 1.448 (1.324-1.585) | <0.001 | 1.138 (1.034-1.253) | 0.008 |
| Low physical activity | 1.253 (1.162-1.352) | <0.001 | 1.062 (0.981-1.148) | 0.136 |
| Slow gait speed | 3.298 (3.015-3.608) | <0.001 | 1.364 (1.231-1.512) | <0.001 |
| Low grip strength | 2.074 (1.930-2.229) | <0.001 | 1.127 (1.043-1.217) | 0.002 |

The multivariate model adjusted for age, sex, ethnicity, education, Townsend deprivation index, average household income, BMI, smoking status, alcohol frequency intake, sleep time, eGFR, C-reaction protein, albumin, high density lipoprotein, low density lipoprotein, triglyceride, total cholesterol, HbA1c, the number of long-term morbidities, history of medication for cholesterol lowering, insulin, antihypertensive and NSAIDS, PRS and five individual components when these were not the exposure.

| Table S18. Risk of incident CKD according to frailty phenotype stratified by different levels of PRS | | | | | |
| --- | --- | --- | --- | --- | --- |
| PRS | Non-frailty | Prefrailty | | Frailty | |
|  | HR (95 % CI) | HR (95 % CI) | P value | HR (95 % CI) | *P* value |
| Low | 1.00 (Ref.) | 1.152 (1.054-1.260) | 0.002 | 1.657 (1.413-1.944) | <0.001 |
| Moderate | 1.00 (Ref.) | 1.088 (1.001-1.183) | 0.048 | 1.531 (1.319-1.777) | <0.001 |
| High | 1.00 (Ref.) | 1.184 (1.096-1.279) | <0.001 | 1.653 (1.439-1.898) | <0.001 |

The results were adjusted for age, sex, ethnicity, education, Townsend deprivation index, average household income, BMI, smoking status, alcohol frequency intake, sleep time, C-reaction protein, eGFR, albumin, high density lipoprotein, low density lipoprotein, triglyceride, total cholesterol, HbA1c, the number of long-term morbidities, history of medication for cholesterol lowering, insulin, antihypertensive and NSAIDS. Ref indicates reference.

The results were adjusted for age, sex, ethnicity, education, Townsend deprivation index, average household income, BMI, smoking status, alcohol frequency intake, sleep time, C-reaction protein, eGFR, albumin, high density lipoprotein, low density lipoprotein, triglyceride, total cholesterol, HbA1c, the number of long-term morbidities, history of medication for cholesterol lowering, insulin, antihypertensive and NSAIDS. Ref indicates reference.

| Table S19. Risk of incident CKD according to PRS stratified by different levels of frailty phenotype | | | | | | |
| --- | --- | --- | --- | --- | --- | --- |
|  | Non-frailty | | Prefrailty | | Frailty | |
| PRS | HR (95 % CI) | P value | HR (95 % CI) | P value | HR (95 % CI) | *P* value |
| Low | 1.00 (Ref.) |  | 1.00 (Ref.) |  | 1.00 (Ref.) |  |
| Moderate | 1.103 (1.009-1.205) | 0.031 | 1.058 (0.978-1.144) | 0.161 | 1.092 (0.913-1.306) | 0.334 |
| High | 1.215 (1.114-1.325) | <0.001 | 1.250 (1.159-1.348) | <0.001 | 1.245 (1.047-1.481) | 0.013 |

| Table S20. Univariable analysis of variables | | |
| --- | --- | --- |
|  | HR (95%Cl) | P |
| Age | 1.107 (1.104-1.111) | <0.001 |
| Sex | 1.344 (1.29-1.4) | <0.001 |
| BMI | 1.072 (1.068-1.076) | <0.001 |
| Smoking | 1.156 (1.083-1.234) | <0.001 |
| Frailty score | 1.441 (1.409-1.474) | <0.001 |
| Sleep time | 1.008 (0.990-1.027) | 0.394 |
| hypertension | 2.327 (2.273-2.476) | <0.001 |
| Diabetes | 3.338 (3.159-3.572) | <0.001 |

| Table S21. Multivariable predictors of CKD | | |
| --- | --- | --- |
|  | HR (95%Cl) | P |
| Age | 1.100 (1.096-1.104) | <0.001 |
| Sex | 1.228 (1.178-1.280) | <0.001 |
| BMI | 1.046 (1.042-1.051) | <0.001 |
| Smoking | 1.386 (1.298-1.480) | <0.001 |
| Frailty score | 1.310 (1.278-1.342) | <0.001 |
| hypertension | 1.399 (1.337-1.463) | <0.001 |
| Diabetes | 1.900 (1.793-2.013) | <0.001 |

| Table S22. CKD risk scoring system | | | | | |
| --- | --- | --- | --- | --- | --- |
| Risk factor | Categories | Reference value (W_ij_) | β_i_ | β_i_ (W_ij_-Wi_REF_) | Points_ij_ = β_i_ (W_ij_-Wi_REF_)/B |
|  |  |  | 0.095234 |  |  |
| Age | 38-47 | **42.5=W_1REF_** |  | 0 | 0 |
|  | 48-57 | 52.5 |  | 0.95234 | 2 |
|  | 58-67 | 62.5 |  | 1.90468 | 4 |
|  | >67 | 70.5 |  | 2.666552 | 6 |
|  |  |  | 0.205050 |  |  |
| Sex | female | **0=W_2REF_** |  | 0 | 0 |
|  | male | 1 |  | 0.205050 | 0.5 |
|  |  |  | 0.045277 |  |  |
| BMI | <18.5 | **16.78=W_3REF_** |  | 0 | 0 |
|  | 18.5-24.9 | 21.7 |  | 0.22276284 | 0.5 |
|  | 25-29.9 | 27.5 |  | 0.48536944 | 1 |
|  | ≥30 | 46.8 |  | 2.116913593 | 3 |
|  |  |  | 0.326374 |  |  |
| Smoking | No | **0=W_4REF_** |  | 0 | 0 |
|  | Current smoking | 1 |  | 0.326374 | 1 |
|  |  |  | 0.269830 |  |  |
| Frailty score | Non-frailty 0 | **0=W_5REF_** |  | 0 | 0 |
|  | Prefrailty 1-2 | 1.5 |  | 0.404745 | 1 |
|  | Frailty 3-5 | 4 |  | 1.07932 | 2 |
|  |  |  | 0.641639 |  |  |
| Diabetes | No | **0=W_6REF_** |  | 0 | 0 |
|  | Yes | 1 |  | 0.641639 | 1 |
|  |  |  | 0.335481 |  |  |
| Hypertension | No | **0=W_7REF_** |  | 0 | 0 |
|  | Yes | 1 |  | 0.335481 | 1 |

The constant B, with each increase of 5 units in age, scores 1 point. The constant B is calculated as 5×0.095234 = 0.47617.

| Table S23. Characteristics among the UKB | | | | |
| --- | --- | --- | --- | --- |
|  | Total | CKD | No CKD | P |
|  | 352,203 | 13,262 | 338,941 |  |
| Age, years | 57 (49-63) | 63 (58-66) | 57 (49-63) | <0.001 |
| Sex |  |  |  | <0.001 |
| Female, n (%) | 185405 (52.6) | 6030 (45.5) | 179375 (52.9) |  |
| BMI, kg/m^2^ | 26.52 (24.01-29.53) | 28.10 (25.34-31.43) | 26.46 (23.97-29.45) | <0.001 |
| Smoking, n (%) |  |  |  | <0.001 |
| Current smoking | 34845 (9.9) | 1456 (11.0) | 33389 (9.9) |  |
| Frailty phenotype, n (%) |  |  |  | <0.001 |
| Non-frailty | 203920 | 6244 (47.1) | 197676 (58.3) |  |
| Prefrailty | 140634 | 6335 (47.8) | 134299 (39.6) |  |
| Frailty | 7649 | 683 (5.2) | 6966 (2.1) |  |
| Frailty score | 0 (0-1) | 1 (0-1) | 0(0-0) | <0.001 |
| Sleep time, hours/day | 7 (7-8) | 7 (6-8) | 7 (7-8) | 0.002 |
| Diabetes, n (%) | 20985 (6.0) | 2154 (16.2) | 18831 (5.6) | <0.001 |
| Hypertension, n (%) | 159647 (45.3) | 8682 (65.5) | 150965 (44.5) | <0.001 |
| Time to CKD, years |  | 9.04 (12.21-13.62) |  |  |

Data are mean (SD), median (IQR), or n (%).

Abbreviations: BMI, body mass index; SD: Standard Deviation; IQR, interquartile range.

| Table S24. Characteristics among the train group and test group in UKB | | | |
| --- | --- | --- | --- |
|  | Train group | Internal validation group | P |
|  | 246,542 | 105,661 |  |
| Age, years | 57 (49-63) | 57 (49-63) | 0.270 |
| Sex |  |  | 0.526 |
| Female, n (%) | 129870 (52.7) | 55535 (52.6) |  |
| BMI, kg/m^2^ | 26.5 (24-29.52) | 27.17 (24.05-29.55) | 0.007 |
| Smoking, n (%) |  |  | 0.820 |
| Current smoking | 24411 (9.9) | 10434 (9.9) |  |
| Frailty phenotype, n (%) |  |  | 0.023 |
| Non-frailty | 143101 (58) | 60819 (57.6) |  |
| Prefrailty | 98079 (39.8) | 42555 (40.3) |  |
| Frailty | 5362 (2.2) | 2287 (2.2) |  |
| Frailty score | 0(0-1) | 0(0-1) | 0.017 |
| Sleep time, hours/day | 7 (7-8) | 7 (7-8) | 0.836 |
| Diabetes, n (%) | 14660 (5.9) | 6325 (6.0) | 0.648 |
| Hypertension, n (%) | 111788 (45.3) | 47859 (45.3) | 0.793 |
| Time to CKD, years | 12.88 (12.14-13.60) | 12.88 (12.13-13.60) | 0.883 |

| Table S25. Characteristics among the CHARLS | | | | |
| --- | --- | --- | --- | --- |
|  | Total | CKD | No CKD | P |
|  | 6,139 | 253 | 5,886 |  |
| Age, years | 58 (52-64) | 67 (60-72) | 58 (51-64) | <0.001 |
| Sex |  |  |  | 0.612 |
| Female, n (%) | 3371 (54.9) | 135 (53.4) | 3236 (55.0) |  |
| BMI, kg/m^2^ | 23.28 (21.06-25.94) | 23.60 (20.78-26.14) | 23.27 (21.08-25.93) | 0.578 |
| Smoking, n (%) |  |  |  | 0.634 |
| Current smoking | 2339 (38.1) | 100 (39.5) | 2239 (38.0) |  |
| Frailty phenotype, n (%) |  |  |  | 0.426 |
| Non-frailty | 437 (7.1) | 13 (5.1) | 424 (7.2) |  |
| Prefrailty | 4351 (73.8) | 193 (76.3) | 4338 (73.7) |  |
| Frailty | 1171 (19.1) | 47 (18.6) | 1124 (19.1) |  |
| Frailty score | 2 (1-2) | 2 (1-2) | 2(1-2) | 0.086 |
| Diabetes, n (%) | 958 (15.6) | 55 (21.7) | 903 (15.3) | 0.006 |
| Hypertension, n (%) | 1547 (25.2) | 105 (41.5) | 1442 (24.5) | <0.001 |

Data are mean (SD), median (IQR), or n (%).

Abbreviations: BMI, body mass index; SD: Standard Deviation; IQR, interquartile range.

**Figure S1. Participant selection for frailty phenotype and CKD cohort study**

**
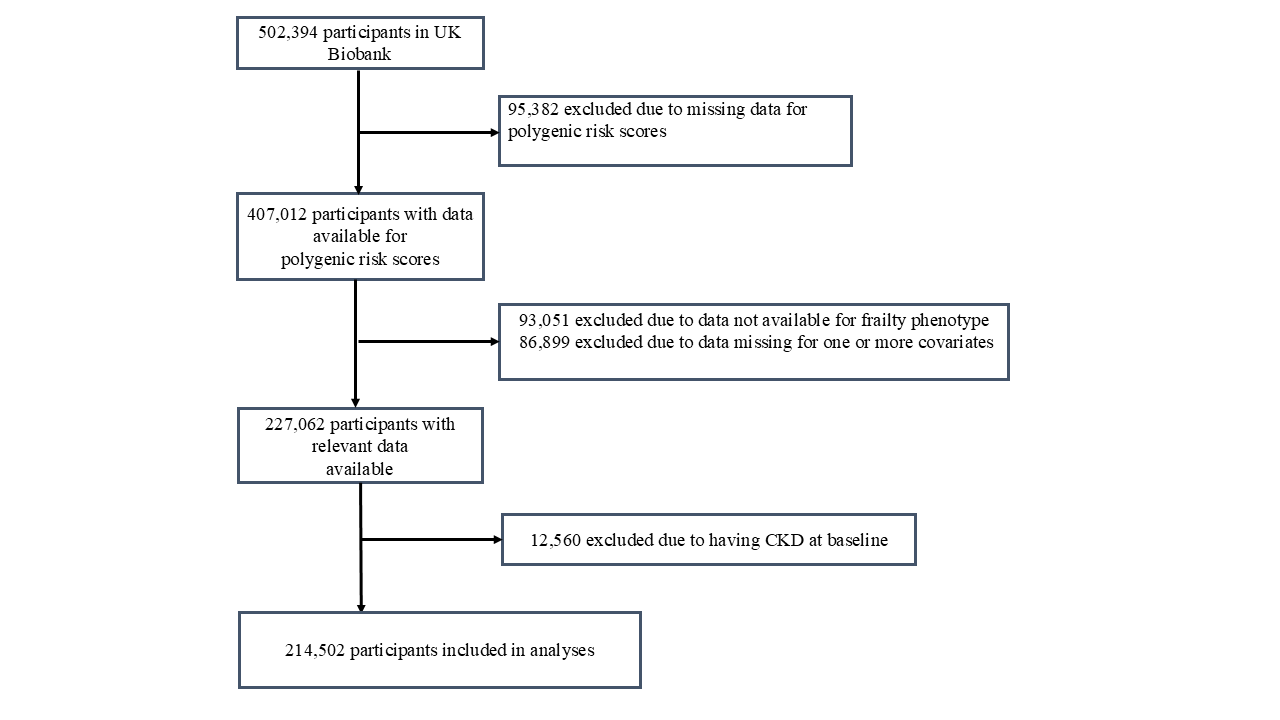
**

**Figure S2. Participant selection for new-onset CKD predictive model in UKB and CHARLS**

**
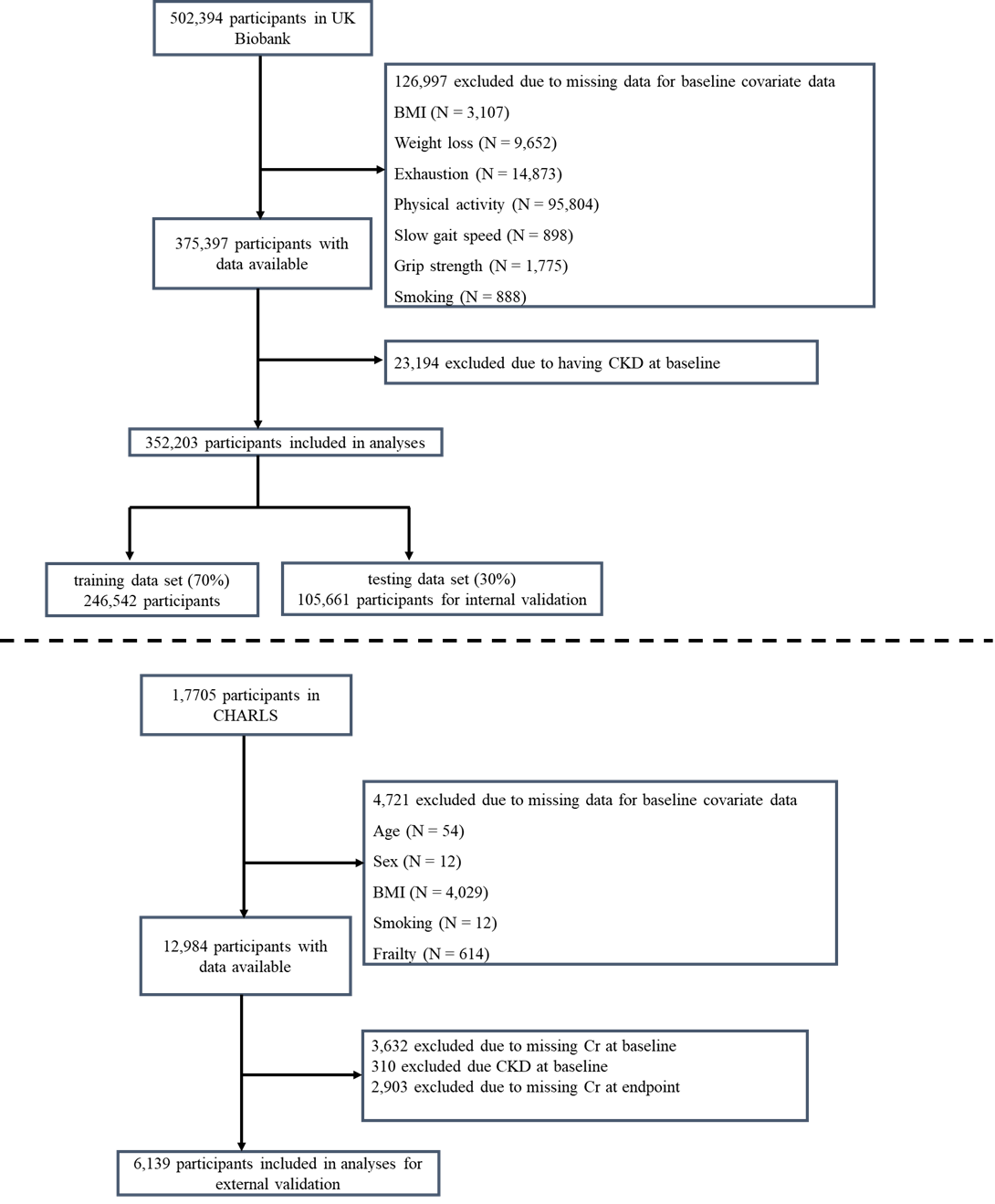
**

**Figure S3. Prevalence of individuals components of the frailty phenotype at baseline**

**
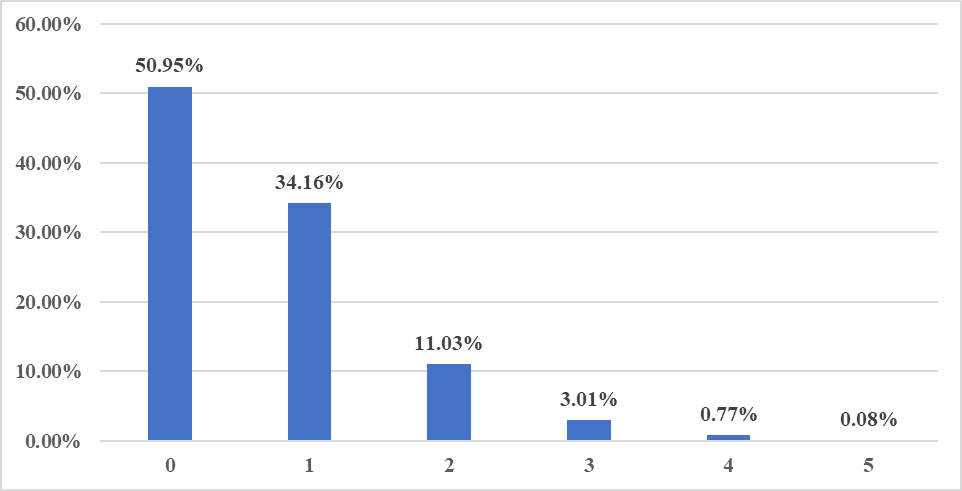
**

**Figure S4.
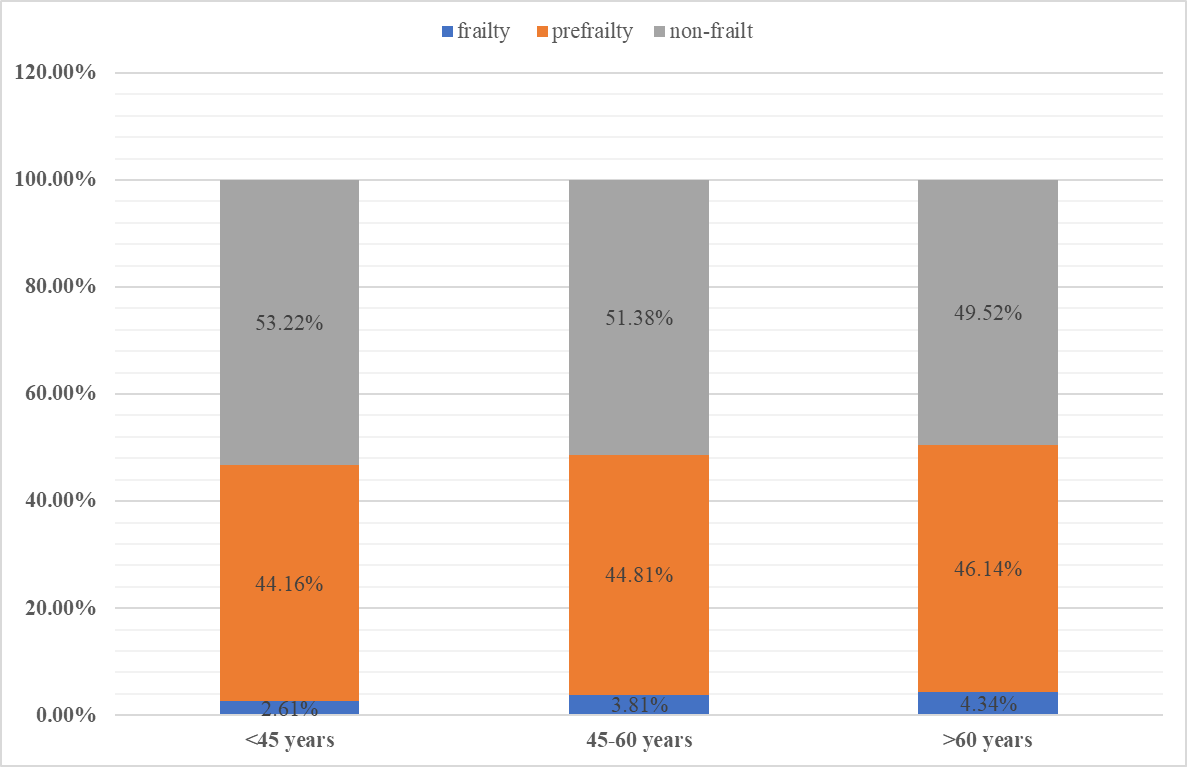
 Age-specific distribution of frailty**


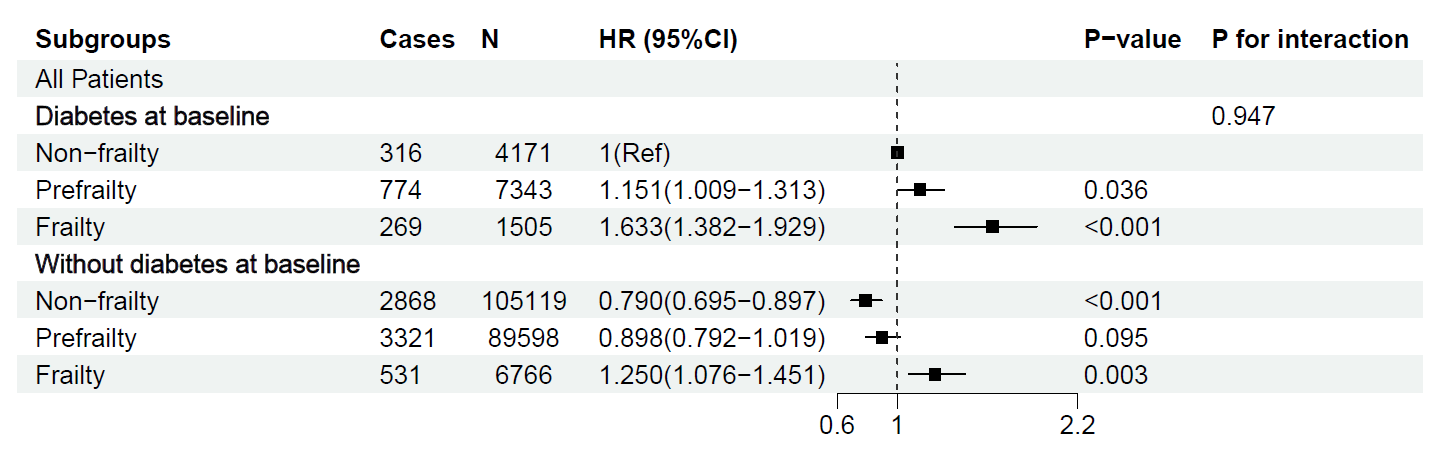
**Figure S5. Combined effect of frailty phenotype and diabetes on new-onset CKD**

The multivariate model adjusted for age, sex, ethnicity, education, Townsend deprivation index, average household income, BMI, smoking status, alcohol frequency intake, sleep time, C-reaction protein, eGFR, albumin, high density lipoprotein, low density lipoprotein, triglyceride, total cholesterol, HbA1c, the number of long-term morbidities, history of medication for cholesterol lowering, insulin, antihypertensive and NSAIDS, PRS. Ref indicates reference.

**Figure S6. Combined effect of frailty phenotype and hypertension on new-onset CKD**

**
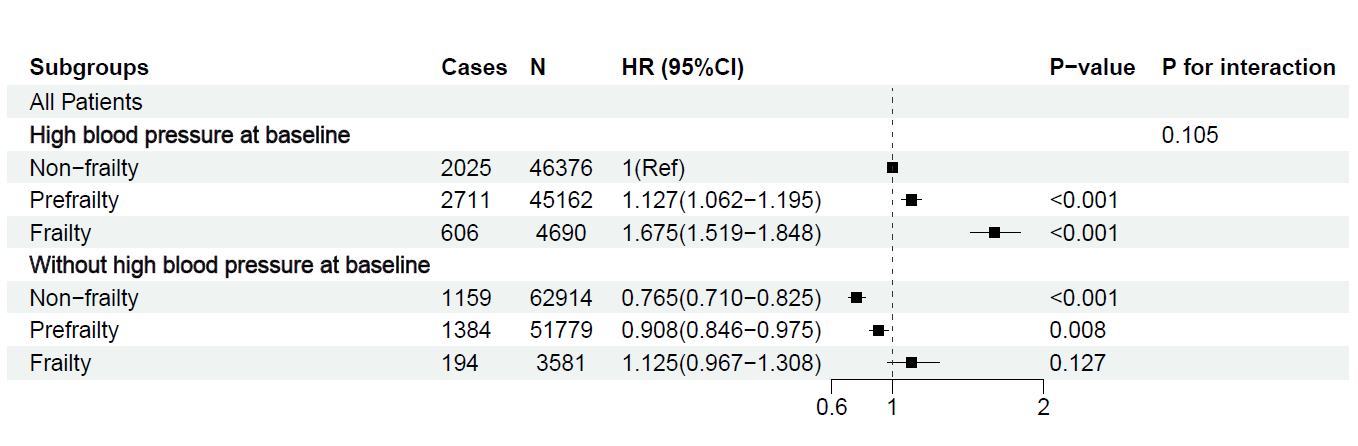
**

The multivariate model adjusted for age, sex, ethnicity, education, Townsend deprivation index, average household income, BMI, smoking status, alcohol frequency intake, sleep time, C-reaction protein, eGFR, albumin, high density lipoprotein, low density lipoprotein, triglyceride, total cholesterol, HbA1c, the number of long-term morbidities, history of medication for cholesterol lowering, insulin, antihypertensive and NSAIDS, PRS. Ref indicates reference.

**Figure S7. Competing risks cumulative incidence curve of frailty phenotype and new-onset CKD**


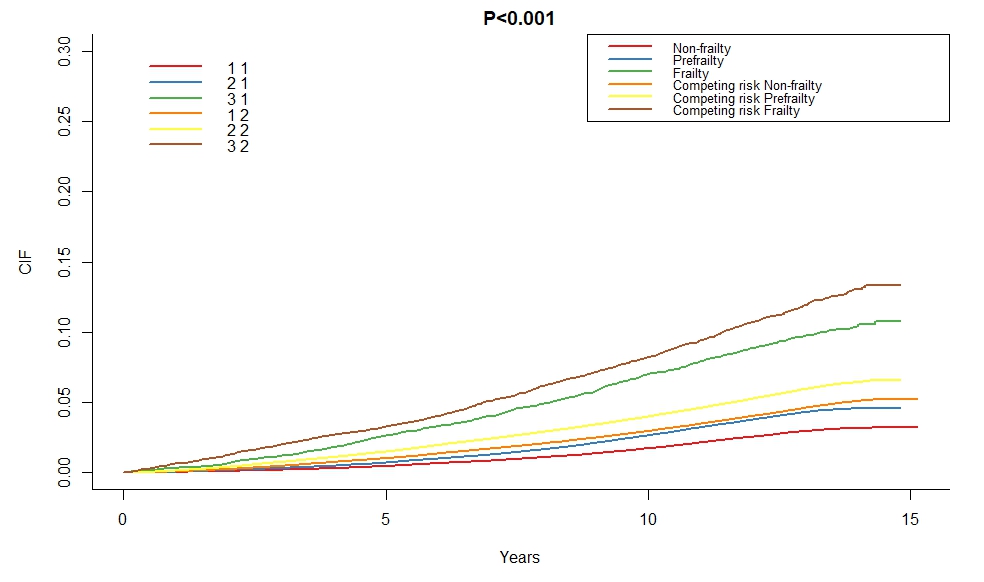


**Figure S8. Prospective association between frailty points and incident CKD in a population excluding baseline CKD defined by ICD codes**


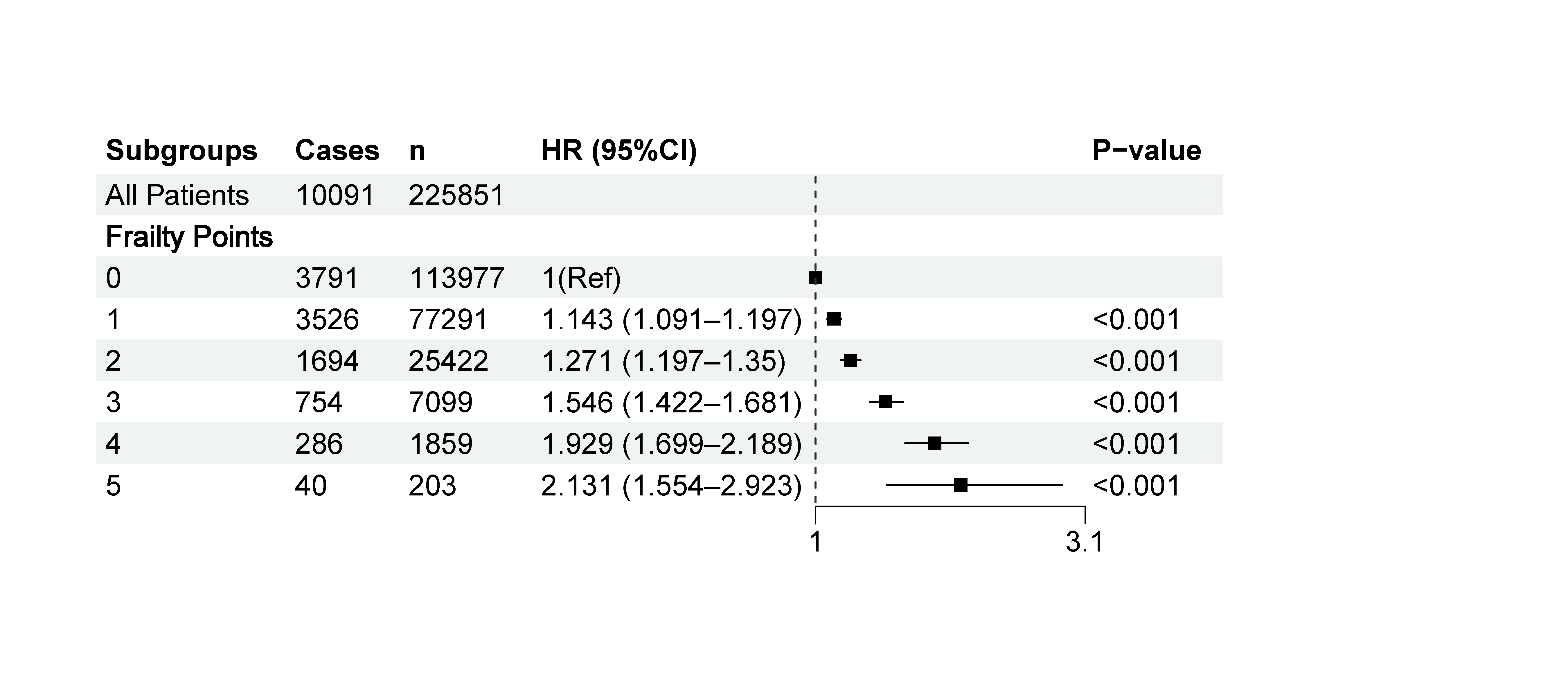
The multivariate model adjusted for age, sex, ethnicity, education, Townsend deprivation index, average household income, BMI, smoking status, alcohol frequency intake, sleep time, eGFR, C-reaction protein, albumin, high density lipoprotein, low density lipoprotein, triglyceride, total cholesterol, HbA1c, the number of long-term morbidities, history of medication for cholesterol lowering, insulin, antihypertensive and NSAIDS, PRS.

**Figure S9.** **Prospective association between frailty points and incident CKD in a population excluding participants with baseline eGFR <75 mL/min/1.73m^2^**


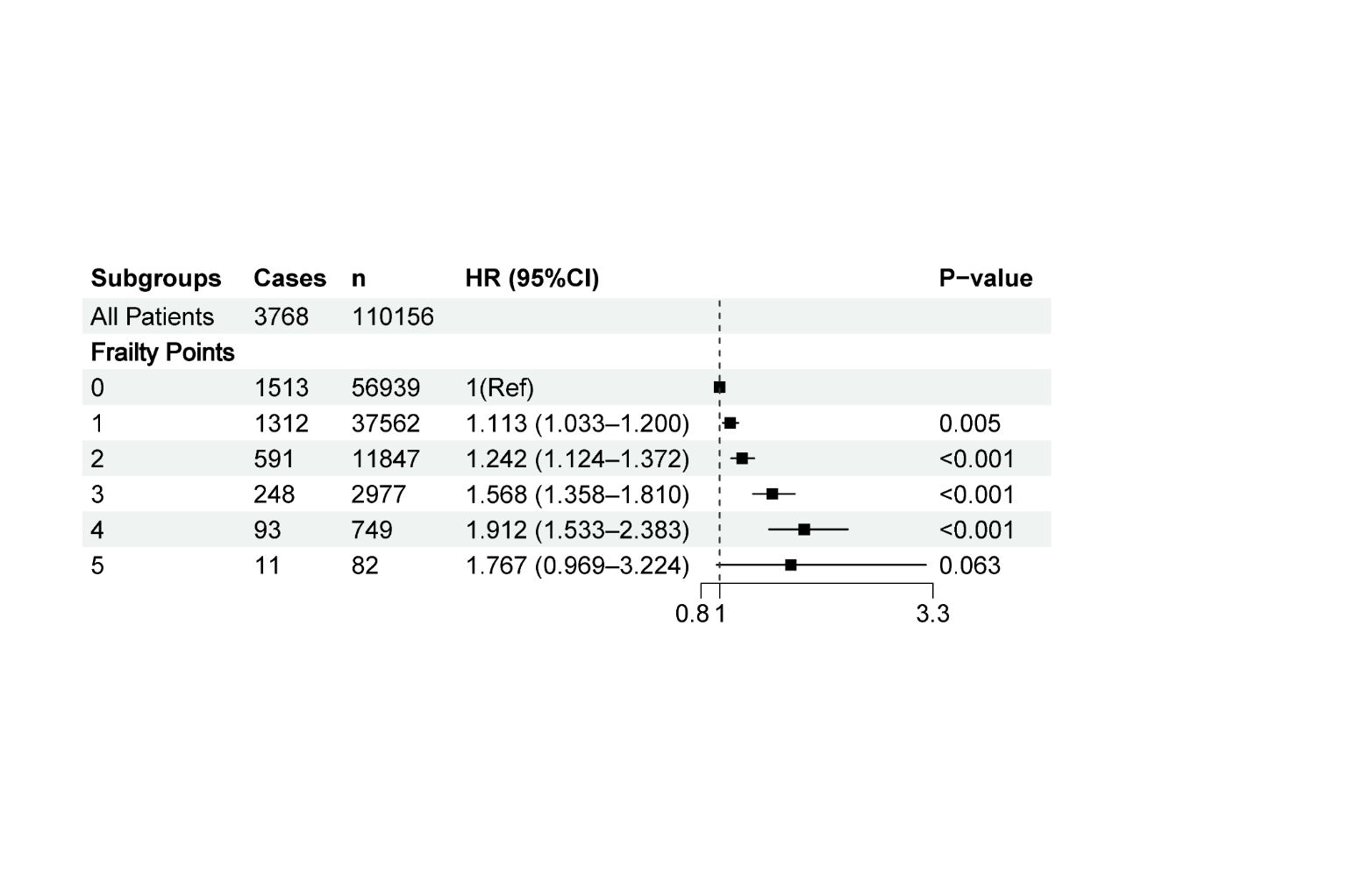


The multivariate model adjusted for age, sex, ethnicity, education, Townsend deprivation index, average household income, BMI, smoking status, alcohol frequency intake, sleep time, eGFR, C-reaction protein, albumin, high density lipoprotein, low density lipoprotein, triglyceride, total cholesterol, HbA1c, the number of long-term morbidities, history of medication for cholesterol lowering, insulin, antihypertensive and NSAIDS, PRS.

**Figure S10. Non-linear associations between age, BMI, frailty score and CKD**


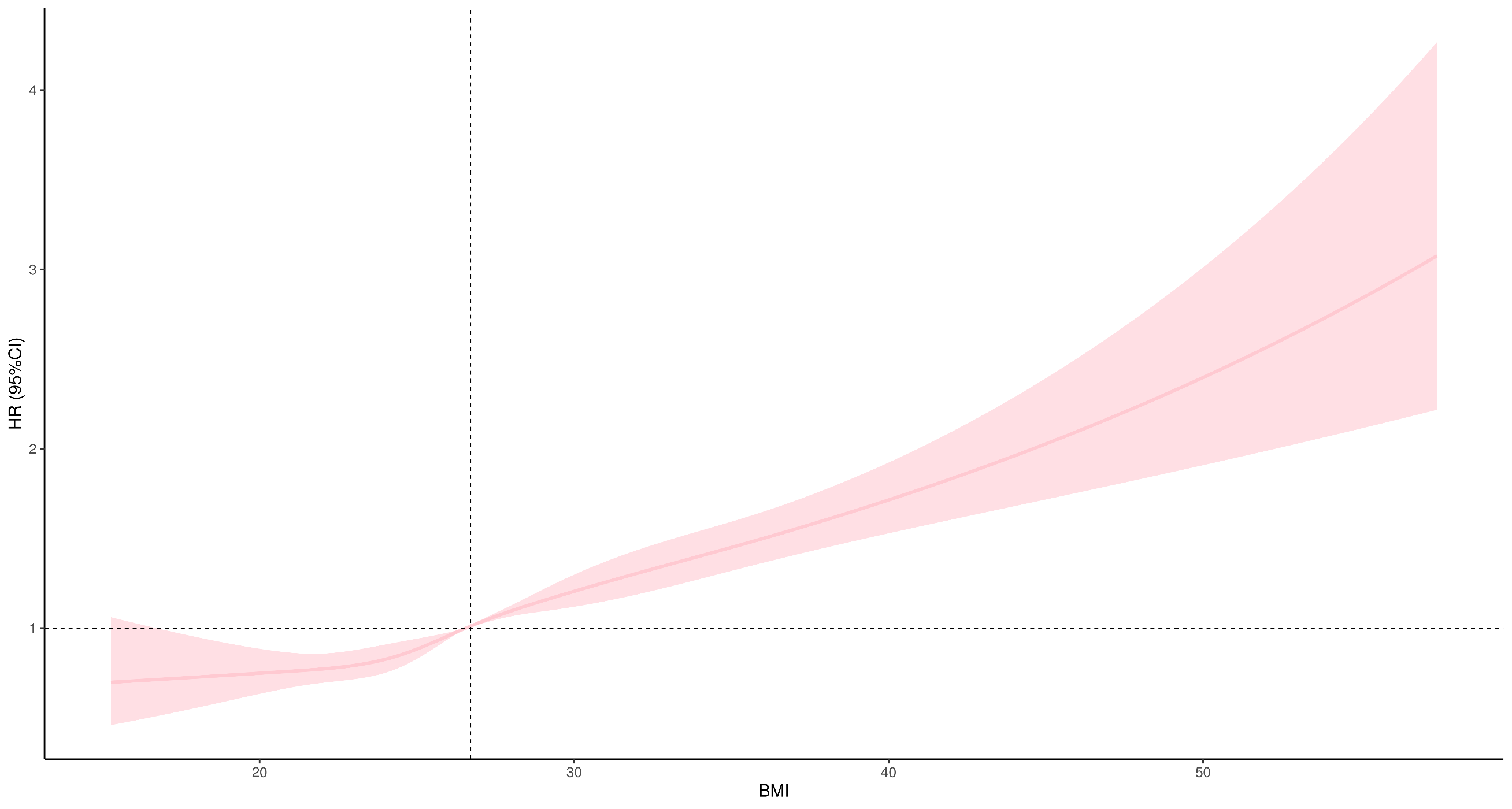

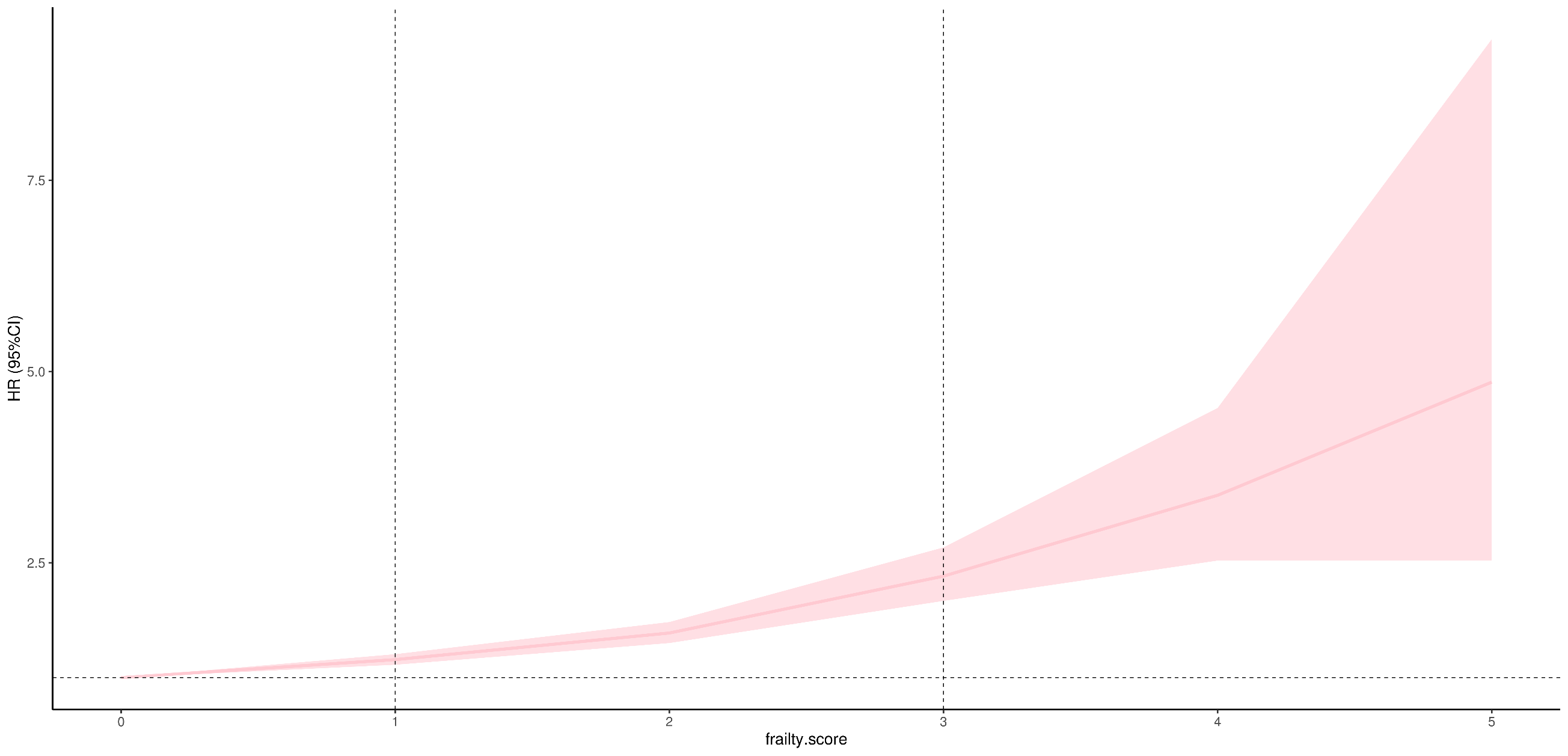

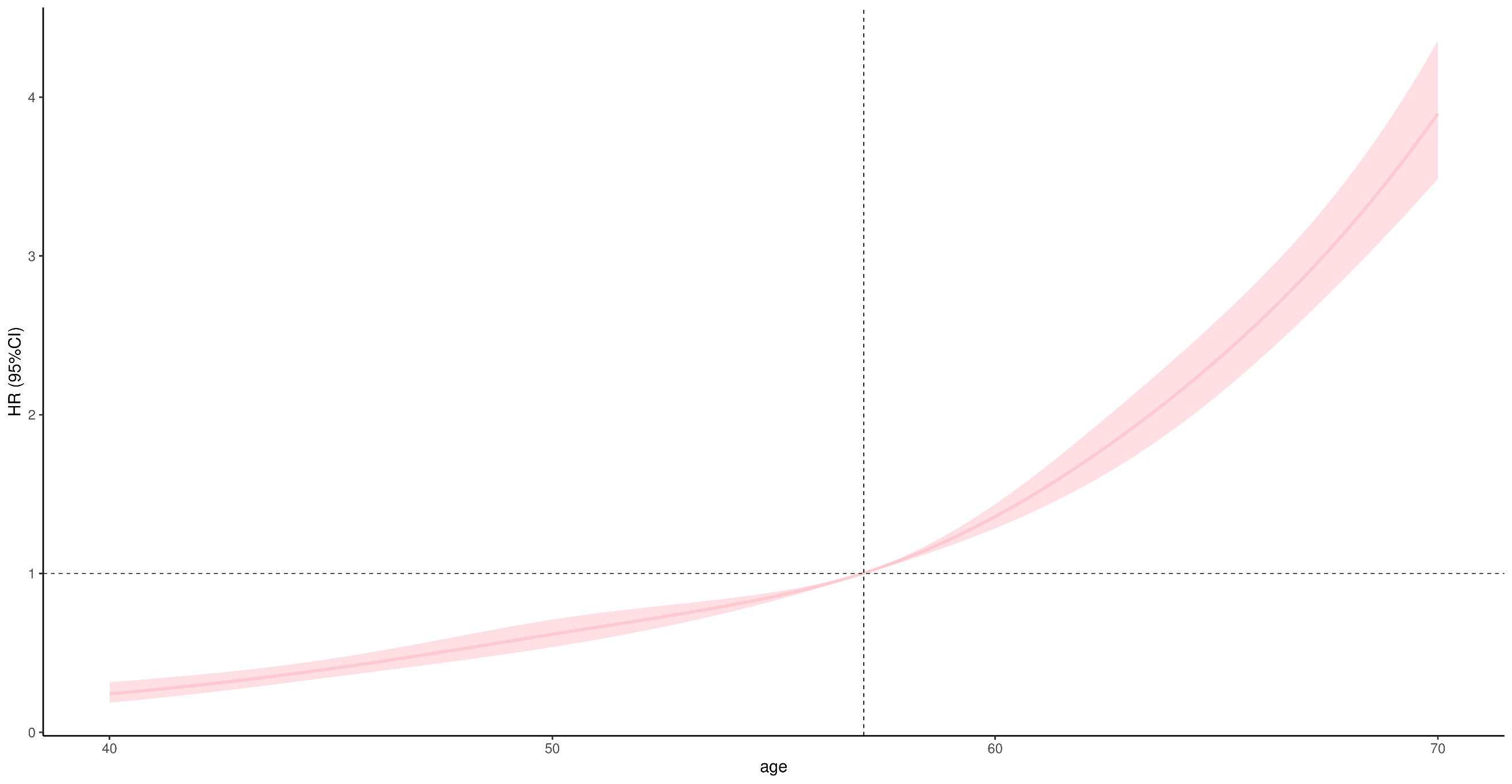


Results were derived from Cox proportional hazard models, co-variables used for adjustment were age, sex, BMI, smoking, frailty score, history of diabetes, history of hypertension. Age: p _non-linear_ = 11.31, p = 0.0101. BMI: p _non-linear_ = 6.94, p = 0.0738. Frailty score: p _non-linear_ = 5.63, p = 0.0599.


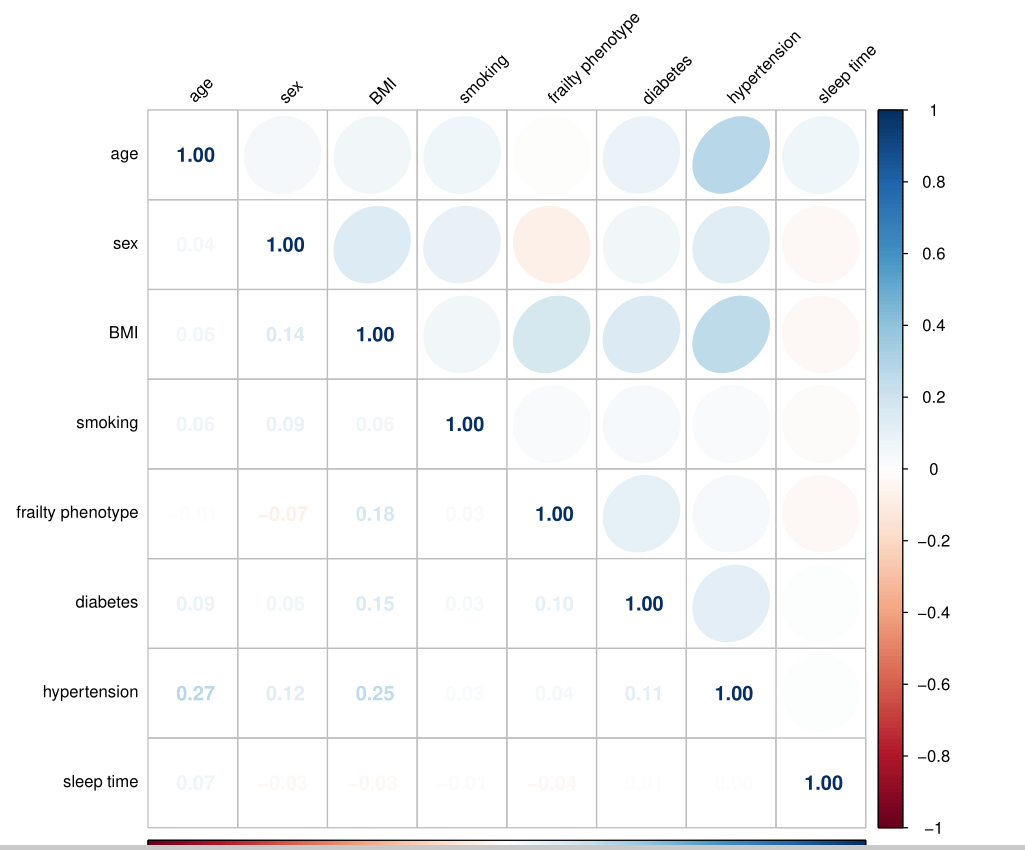
**Figure S11. Spearman coefficient correlation between CKD prediction factors**

**Figure S12. Regression coefficient variation curve with Log (λ)**

**
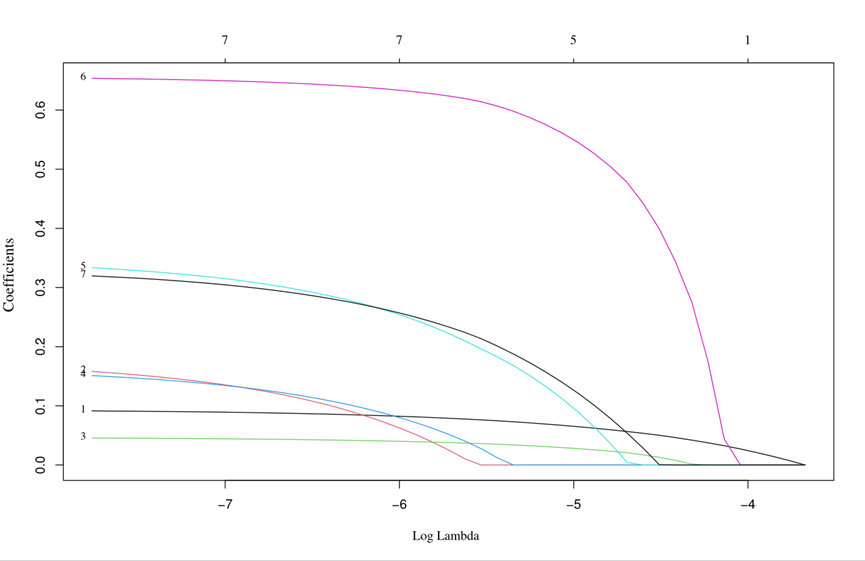
**

**Figure S13. The variation of mean square error with log (λ) in Lasso regression**


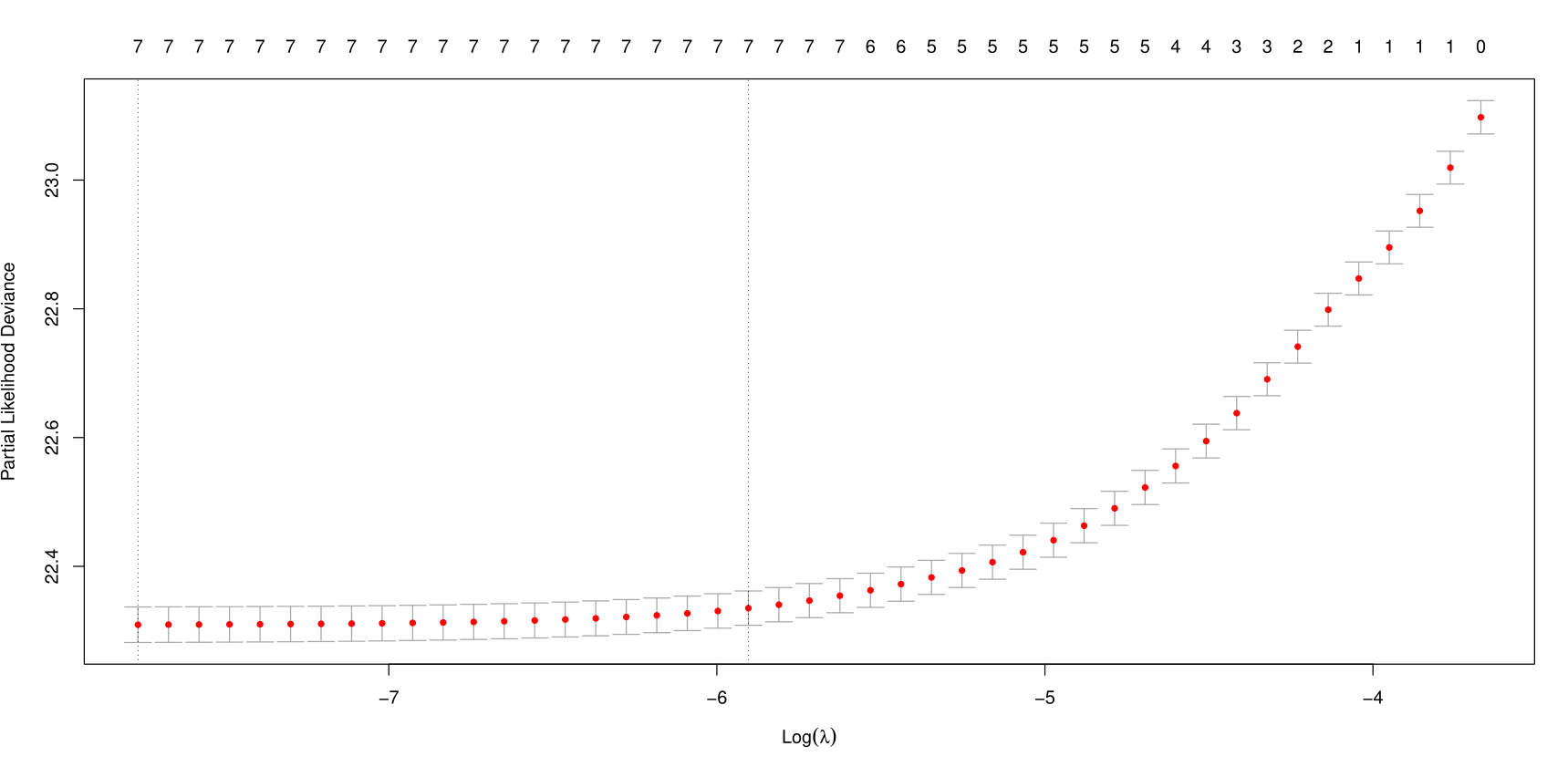


**Figure S14. Performance of the model in predicting the risk of CKD in the UKB training cohort at 5, 8 and 10 years. AUC, area under the curve**

**
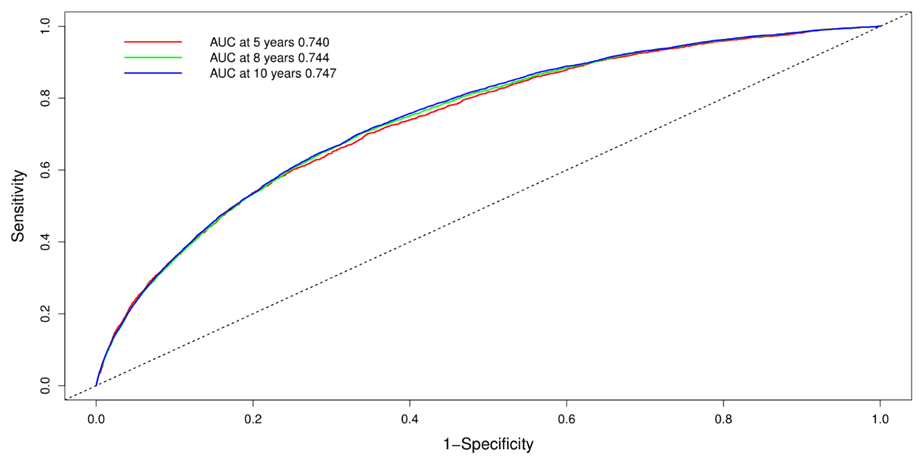
**

**Figure S15. Discriminative performance of the model in predicting CKD risk in the UKB internal validation at 5, 8, and 10 years, based on CKD definitions using ICD codes. AUC: area under the curve**


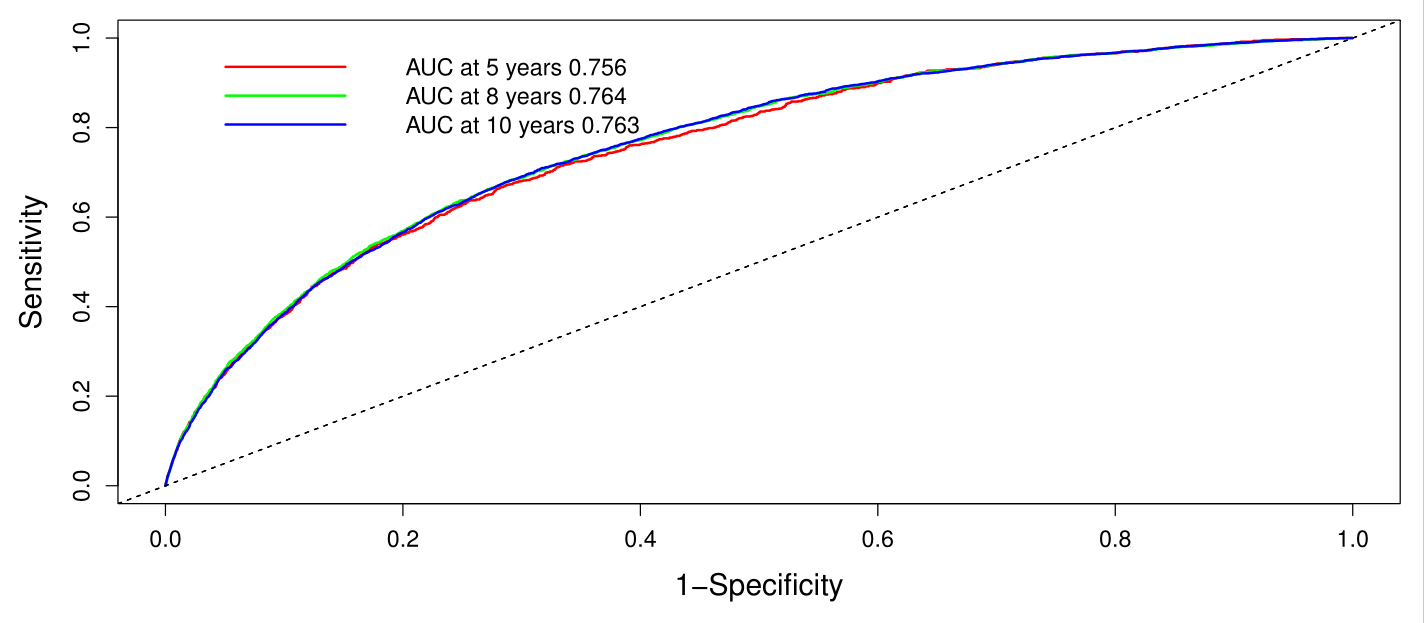


**Figure S16. Calibration plots showing the agreement between predicted and observed risk of CKD in the UKB internal validation cohort, using ICD code–based definitions of CKD**

**
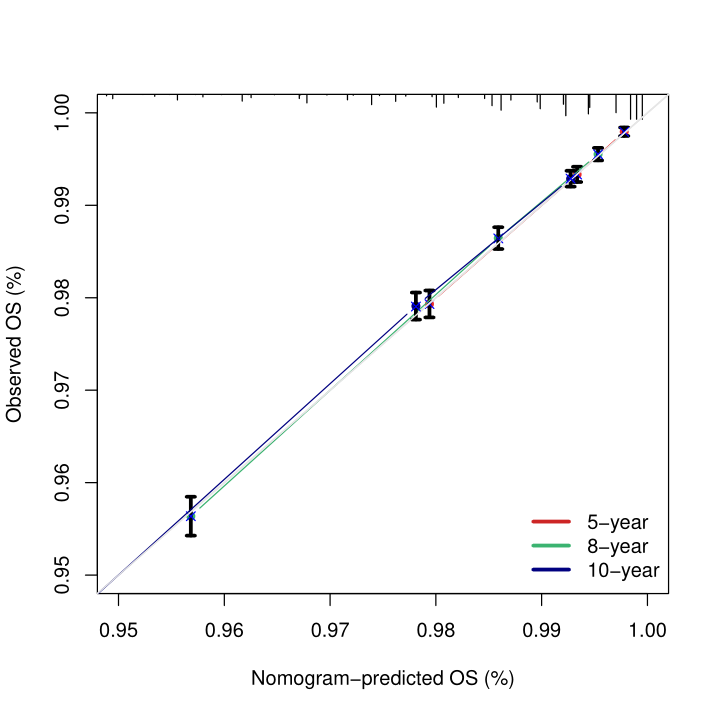
**

**Figure S17. Discriminative performance of the model in predicting CKD risk in the UKB internal validation at 5, 8, and 10 years, excluding participants with baseline eGFR <****75 mL/min/1.73m² AUC: area under the curve**

**
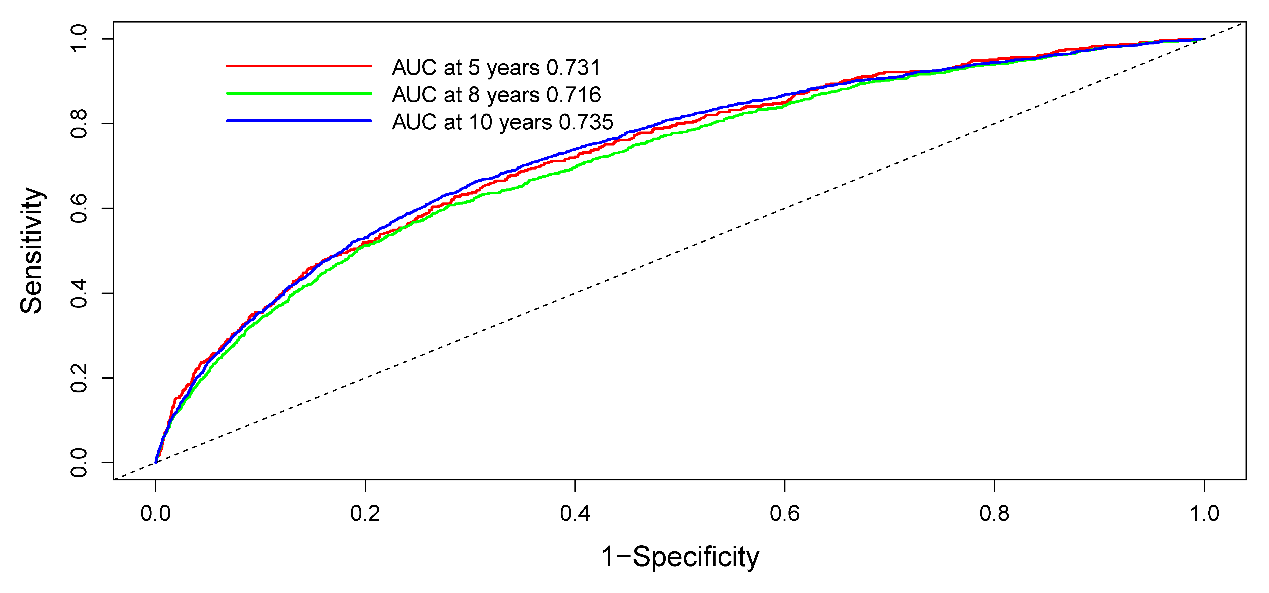
**


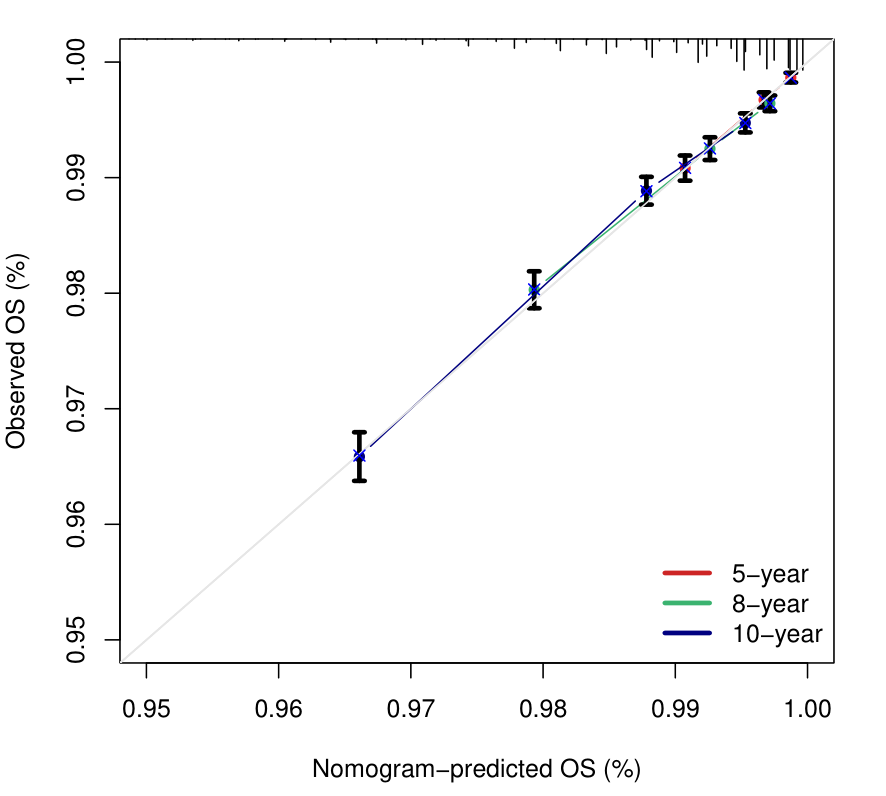
**Figure S18. Calibration plots showing the agreement between predicted and observed risk of CKD based on the CKD risk score in the UKB internal validation cohort, excluding participants with baseline eGFR <75 mL/min/1.73m²**

**Figure S19. Calibration plots for observed and risk of CKD used CKD risk score in UKB internal validation cohort**


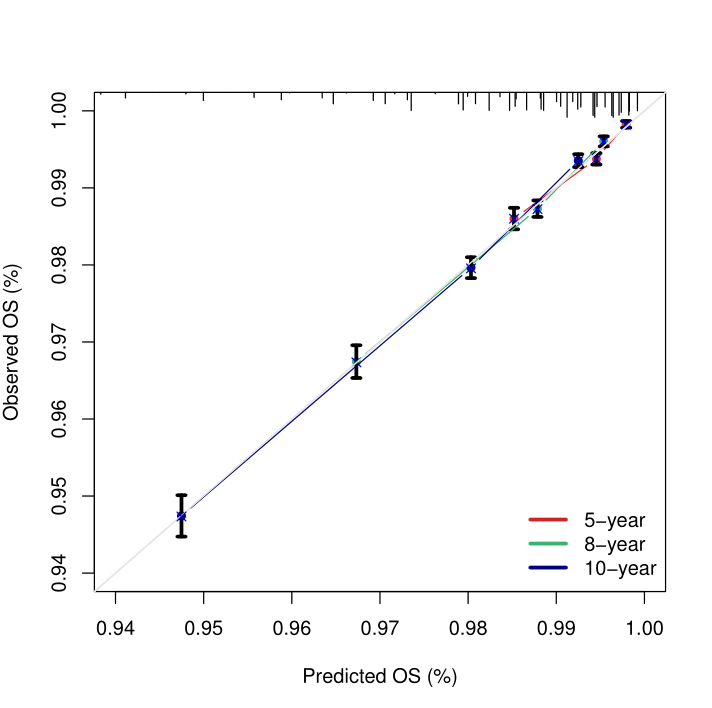


**Figure S20.** **Performance of the CKD risk score in predicting the risk of CKD in the UKB training cohort and testing cohort at 5, 8 and 10 years. AUC, area under the curve**


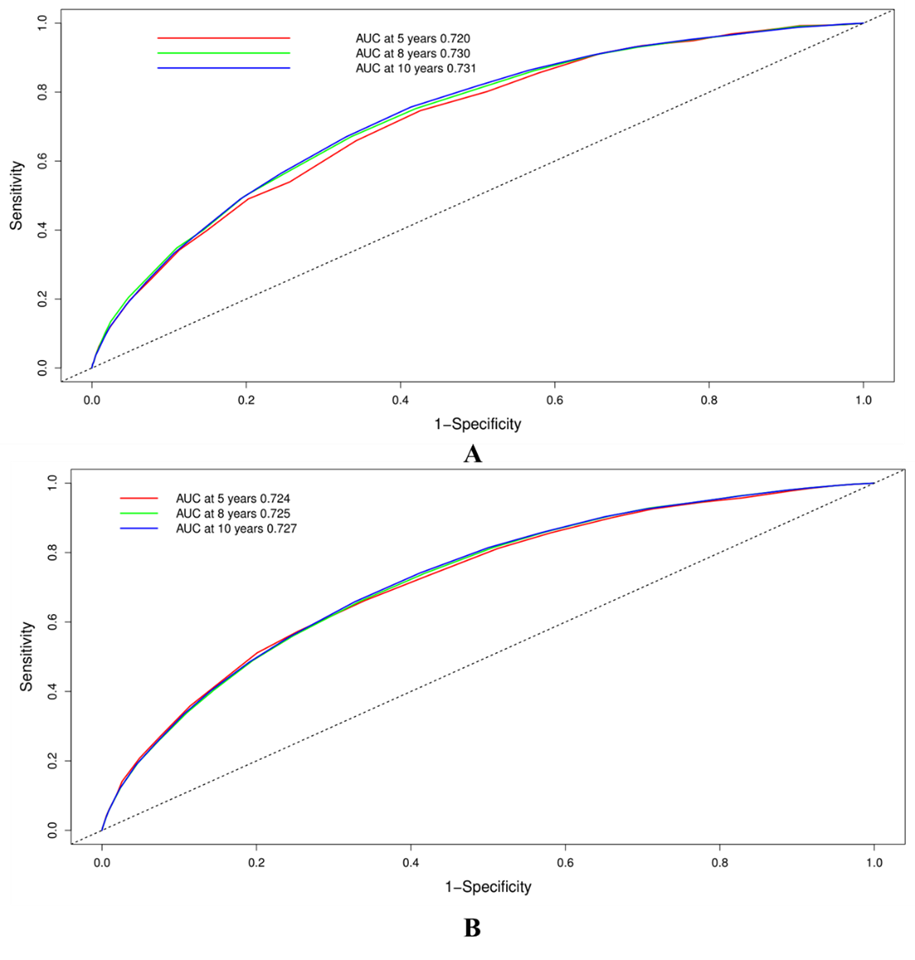


**A: Performance of the CKD risk score in predicting the risk of CKD in the UKB internal validation cohort**

**B: Performance of the CKD risk score in predicting the risk of CKD in the UKB training cohort**

**Figure S21. Cumulative incidence of CKD according to CKD risk score in internal validation cohort of the UKB. Log-rank tests *p*＜0.001.**

**
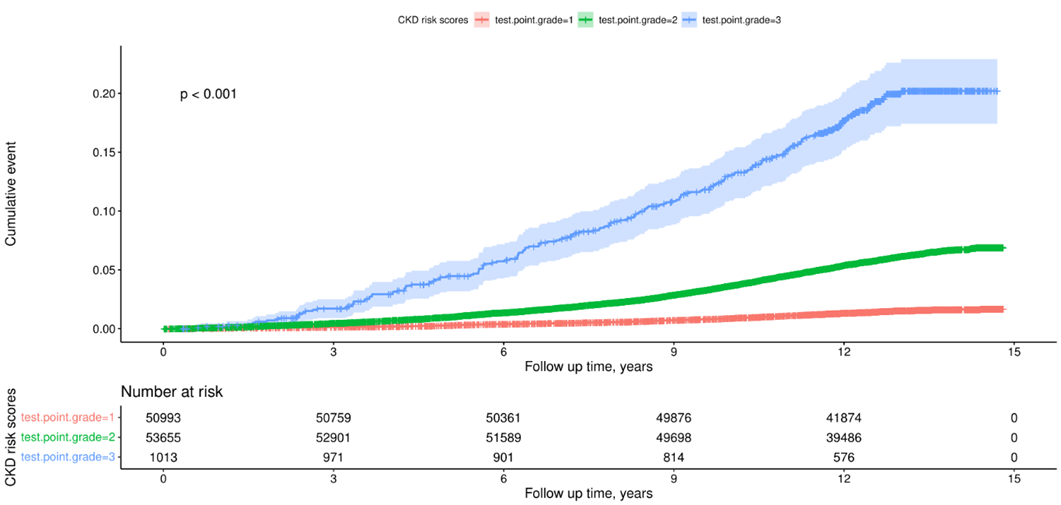
**


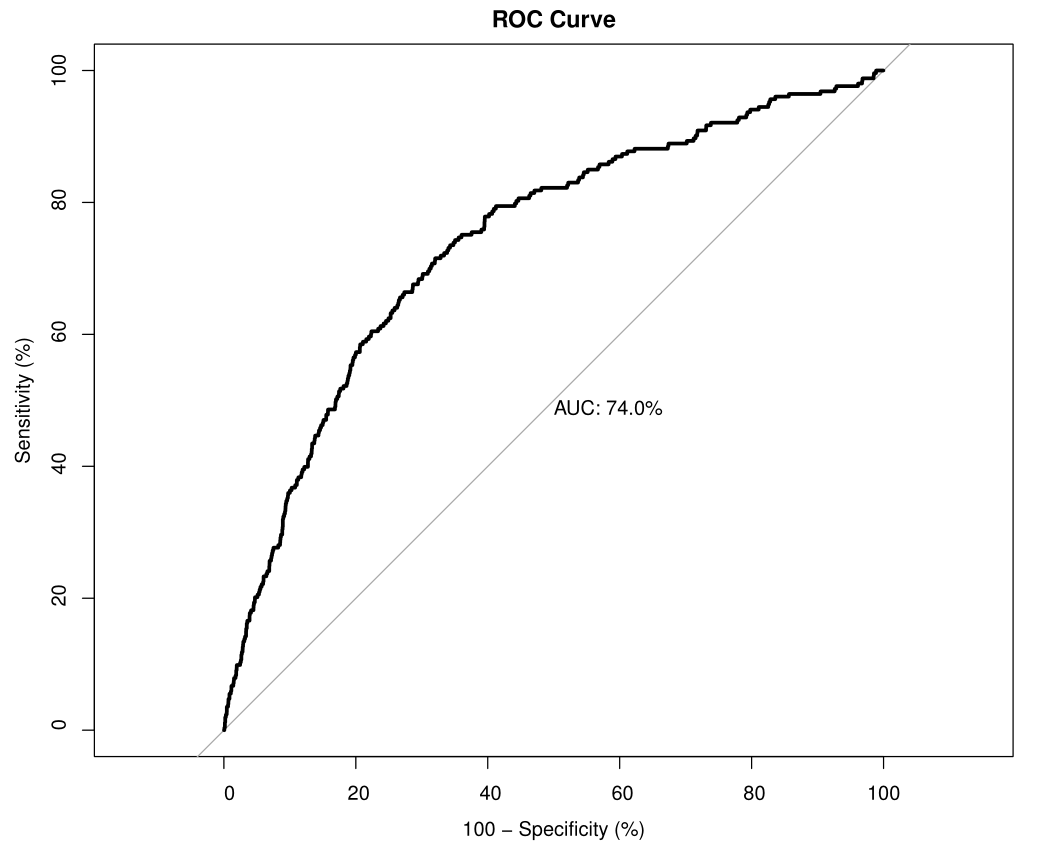
**Figure S22. Performance of the CKD model in predicting the risk of CKD in CHARLS. AUC, area under the curve**

**Figure S23. Performance of the CKD risk score in predicting the risk of CKD in CHARLS. AUC, area under the curve**


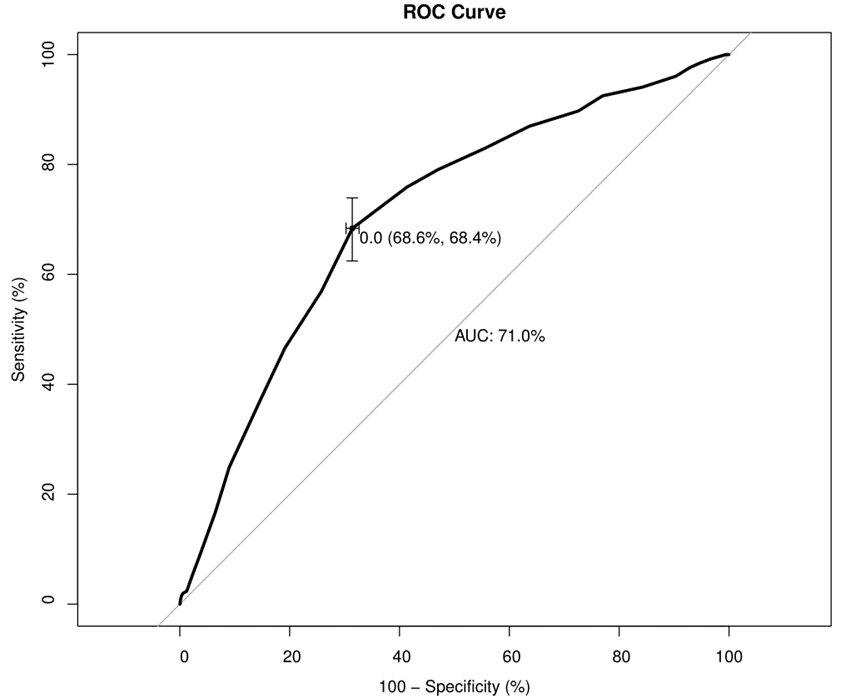


**Figure S24.
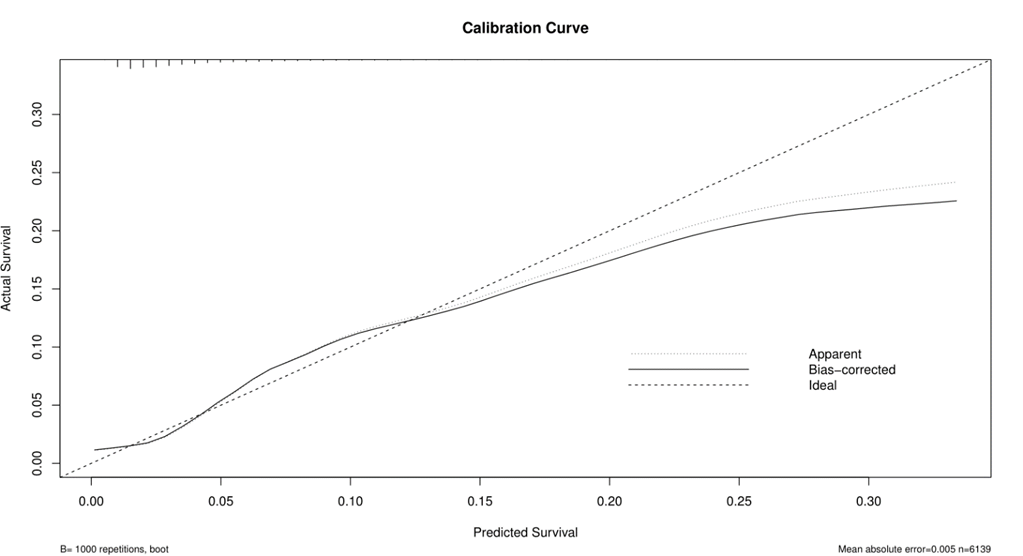
 Calibration plots for observed and risk of CKD in CHARLS**


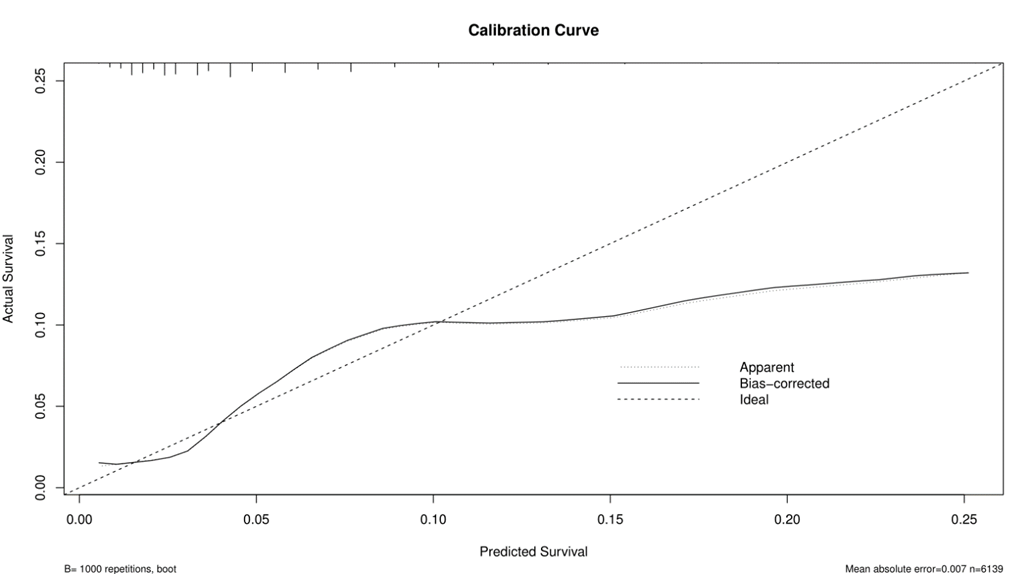
**Figure S25. Calibration plots for observed and risk of CKD used CKD risk score in CHARLS**

**Supplementary References**

**S1. Petermann-Rocha F, Lyall DM, Gray SR, Esteban-Cornejo I, Quinn TJ, Ho FK, et al. Associations between physical frailty and dementia incidence: a prospective study from UK Biobank. The Lancet Healthy Longevity. 2020;1:e58-e68.**

**S2. Wu S, Yang Z, Liu S, Zhang Q, Zhang S, Zhu S. Frailty status and risk of irritable bowel syndrome in middle-aged and older adults: A large-scale prospective cohort study. EClinical Medicine. 2023;56:**

**S3. Wu Y, Xiong T, Tan X, Chen L. Frailty and risk of microvascular complications in patients with type 2 diabetes: a population-based cohort study. BMC medicine. 2022;20:1-13.**

**S4.** **Hanlon P, Nicholl BI, Jani BD, Lee D, McQueenie R, Mair FS. Frailty and pre-frailty in middle-aged and older adults and its association with multimorbidity and mortality: a prospective analysis of 493 737 UK Biobank participants. Lancet Public Health. 2018;3:e323-e32. doi:10.1016/s2468-2667(18)30091-4**
